# Evaluation using latent class models of the diagnostic performances of three ELISA tests commercialized for the serological diagnosis of *Coxiella burnetii* infection in domestic ruminants

**DOI:** 10.1186/s13567-021-00926-w

**Published:** 2021-04-14

**Authors:** Thibaut Lurier, Elodie Rousset, Patrick Gasqui, Carole Sala, Clément Claustre, David Abrial, Philippe Dufour, Renée de Crémoux, Kristel Gache, Marie Laure Delignette-Muller, Florence Ayral, Elsa Jourdain

**Affiliations:** 1 UMR EPIA, Université Clermont Auvergne, INRAE,VetAgro Sup, route de Theix, 63122 Saint-Genès-Champanelle, France; 2 Usc 1233 RS2GP, Université de Lyon, INRAE,VetAgro Sup, 1 avenue bourgelat, 69280 Marcy l’Etoile, France; 3 UMR EPIA, Université de Lyon, INRAE, VetAgro Sup, 1 avenue bourgelat, 69280 Marcy l’Etoile, France; 4 Animal Q Fever Unit, Epidemiology and Support To Surveillance Unit, French Agency for Food, Environmental and Occupational Health & Safety (ANSES), Sophia Antipolis Laboratory, Sophia Antipolis, France; 5 Epidemiology and Support To Surveillance Unit, French Agency for Food, Environmental and Occupational Health & Safety (ANSES), University of Lyon-ANSES Lyon, Lyon, France; 6 French Livestock Institute, Ruminant Health Management Joint Unit, Paris, France; 7 GDS France (National Animal Health Farmers’ Organization), Paris, France; 8 UMR 5558, Laboratoire de Biométrie Et Biologie Evolutive, Université de Lyon, Université Lyon 1, VetAgro Sup, CNRS, 69622 Villeurbanne, France

**Keywords:** Q fever, Latent class model, Bayesian, Diagnostic accuracy, Herd sensitivity, Conditional dependence, Cattle, Sheep, Goats

## Abstract

ELISA methods are the diagnostic tools recommended for the serological diagnosis of *Coxiella burnetii* infection in ruminants but their respective diagnostic performances are difficult to assess because of the absence of a gold standard. This study focused on three commercial ELISA tests with the following objectives (1) assess their sensitivity and specificity in sheep, goats and cattle, (2) assess the between- and within-herd seroprevalence distribution in these species, accounting for diagnostic errors, and (3) estimate optimal sample sizes considering sensitivity and specificity at herd level. We comparatively tested 1413 cattle, 1474 goat and 1432 sheep serum samples collected in France. We analyzed the cross-classified test results with a hierarchical zero-inflated beta-binomial latent class model considering each herd as a population and conditional dependence as a fixed effect. Potential biases and coverage probabilities of the model were assessed by simulation. Conditional dependence for truly seropositive animals was high in all species for two of the three ELISA methods. Specificity estimates were high, ranging from 94.8% [92.1; 97.8] to 99.2% [98.5; 99.7], whereas sensitivity estimates were generally low, ranging from 39.3 [30.7; 47.0] to 90.5% [83.3; 93.8]. Between- and within-herd seroprevalence estimates varied greatly among geographic areas and herds. Overall, goats showed higher within-herd seroprevalence levels than sheep and cattle. The optimal sample size maximizing both herd sensitivity and herd specificity varied from 3 to at least 20 animals depending on the test and ruminant species. This study provides better interpretation of three widely used commercial ELISA tests and will make it possible to optimize their implementation in future studies. The methodology developed may likewise be applied to other human or animal diseases.

**Supplementary Information:**

The online version contains supplementary material available at 10.1186/s13567-021-00926-w.

## Introduction

Q fever is a zoonotic disease, caused by the bacterium *Coxiella burnetii*, responsible annually for many infections in humans worldwide, although precise statistics are lacking. As examples, 193 and 794 confirmed human clinical cases were reported in the United States in 2017 [1] and in Europe in 2018, respectively [2]. Although most Q fever infections remain asymptomatic, the disease may be highly debilitating in certain risk groups and lead to chronic disease. Moreover, Q fever outbreaks occur regularly and involve substantial numbers of people. The record was observed in the Netherlands between 2007 and 2010 with more than 4000 human cases reported [3].

In domestic ruminants, infected animals are often asymptomatic, but infection may cause late abortion, stillbirth, premature delivery or delivery of weak offspring. The European Food Safety Authority (EFSA) reported that 11% of tested goats and sheep, and 7.6% of tested cattle were positive for Q fever in 2018 in Europe [4]. However, the results obtained in different countries through various studies are difficult to compare because of differences in targeted populations, sampling strategies, types of samples analyzed, and diagnostic tests performed [4]. Harmonization of data collection is critical in order to obtain a more accurate picture of the situation and of its evolution over time.

As an example, in France, the latest study on the seroprevalence of *C. burnetii* infection in domestic ruminants was carried out from 2012 to 2015 in ten administrative departments on a total of 10 040 cows, 7776 ewes and 5246 goats sampled from 731 cattle, 522 sheep and 349 goat herds, respectively [5]. The between-herd seroprevalence levels were assessed at 36.0% for cattle, 55.7% for sheep, and 61.0% for goat herds [5]; these estimates were made assuming a sensitivity (Se) and a specificity (Sp) of 100% for the ELISA test used because reliable information about its diagnostic performances was lacking. This assumption may have led to over- or under- estimating the seroprevalence because false-positive and false-negative animals were not accounted for: knowledge of diagnostic test characteristics is crucial to build accurate monitoring systems [6].

To date, no gold standard (i.e. a test with a Se and a Sp equal to 100%) is available for the serological diagnosis of *C. burnetii* infection in animals [7]. Absence of a gold standard limits the assessment of diagnostic test characteristics on a representative sample of the target population, i.e., the population in which the tests are performed in practice, because individual true status of seropositivity or seronegativity is unknown. As a consequence, several studies have investigated the agreement between methods used for the serological diagnosis of *C. burnetii* infection without assessing their respective Se and Sp [8–14]. In the absence of a gold standard test, latent class models (LCMs) [15] can be useful to assess the Se and Sp of multiple diagnostic tests without knowing the true status of the tested individuals. These models are based on the evaluation of the cross-classified test results of multiple binary tests in multiple populations. The quantitative measures of semi-quantitative tests such as ELISAs are generally included in these models as binary variables, considering the positivity threshold provided by manufacturers. LCMs rely on the hypothesis that diagnostic tests measure a common unobserved latent status, for example “the presence or absence of *C. burnetii*-specific antibodies in a serum sample”. In addition, the development of free software environments such as WinBUGS [16] or JAGS [17] facilitates the use of these models in the assessment of diagnostic tests for animal and human diseases [18, 19], including *C. burnetii* infection in domestic ruminants [20–24].

There are currently three commercially available ELISA tests to detect the presence of *C. burnetii*-specific antibodies in domestic ruminants. Because the antigens and conjugates used in these tests are not the same, the respective diagnostic performances are expected to vary depending on the test considered and the animal species. Five previous studies have used LCMs to investigate the diagnostic performances of these tests [21–24], considering either the three ELISA tests [20], or two of them [21, 24], or one ELISA test and another method such as indirect immunofluorescence assay (IFA) or complement fixation test (CFT) [22, 23]). In these studies, conditional dependence between tests was neglected [20, 24] or estimated at low values [21–24]. However, the assumption of conditional independence between tests is generally not satisfied when the considered tests rely on the same biological process. For example, diagnostic errors of two different ELISA methods detecting antibodies against *C. burnetii* are likely to be dependent for both truly seropositive and truly seronegative animals. In fact, an individual with a low level of circulating antibodies against *C. burnetii* will probably be false-negative with both ELISA methods; similarly, a false-positive result, due to the exposure of the animal to a bacterium presenting antigens that cross-react with *C. burnetii*, will probably occur with both serological tests.

Many approaches have been proposed to model conditional dependence between tests [25–30] but they are all confronted with identifiability issues. Moreover, models with different conditional dependence structures might lead to different Se and Sp estimates when applied to the same data [31, 32]. Fixed effect models, which take into account excess probabilities of having a concordant result between each pair of tests, for truly seropositive and truly seronegative individuals, respectively are the most commonly used [18, 29]. However, when more than two tests are considered, these models may be biased, particularly in the case of higher than pairwise conditional dependence structure [28, 33]. In 2017, Wang et al. [33] proposed a new fixed effect model, which made it possible to assess conditional dependence between multiple diagnostic tests (more than two), without restricting only to pairwise conditional dependence.

In this manuscript, we applied this method to model conditional dependence between the three ELISA tests routinely used to assess the serological status of *C. burnetii* infection in domestic ruminants in France. The objectives of the study were to (1) assess the respective Se and Sp of the tests in sheep, goats and cattle, (2) assess the between- and within-herd seroprevalence distribution in these species while accounting for diagnostic errors, and (3) estimate optimal sample sizes considering Se and Sp at the herd level.

## Materials and methods

### Materials

Serum samples originated from the broader epidemiological study (referred to as the “original survey” in the following text) carried out between 2012 and 2015 (for details, see Gache et al. [5]); briefly, 11 to 15 parous females were sampled from a total of 731 cattle, 522 sheep and 349 goat herds, randomly selected in each species and administrative department of France, and excluding herds vaccinated against Q fever. During this study, the national reference laboratory for Q fever (NRL, ANSES, Sophia Antipolis) asked each department for their first 150 serum samples in each species, including complete series (i.e. all the serum samples from the same herd). Overall, serum samples from 1413 cows from 106 herds, 1474 goats from 103 herds, and 1432 sheep from 99 herds were considered (Table 1). As only parous females were sampled, we defined the herd size as the number of parous females in the herd. For goat and sheep herds, it was given by the farmer and recorded by the veterinarian in charge of collecting blood samples. For cattle, the number of parous females at the sampling date was directly retrieved from the French National Cattle Identification Database (BDNI). When the herd size was missing (which was the case for two cattle herds, 34 goat herds, and 33 sheep herds), we imputed this number with the median herd size in the corresponding species.

**Table 1:** **Number of serum samples analyzed per herd and department**

Species	Number	Department										Total
		A	B	C	D	E	F	G	H	I	J	
Cattle	Herds	10	12	11	13	12	12	10	12	13	1	106
	Animals	143	157	150	181	155	161	155	150	152	9	1413
Goats	Herds	11	11	12	12	11	9	11	1	12	13	103
	Animals	154	161	201	175	152	134	146	11	153	187	1474
Sheep	Herds	11	11	10	10	11	11	11	10	11	3	99
	Animals	165	162	149	145	155	157	161	146	156	36	1432

All serum samples were analyzed by the NRL with the three commercial ELISA tests available in France. To our knowledge, all three tests detect immunoglobulin G (IgG) and use antigens based on a mix of *C. burnetii* phase 1 and phase 2 variants. However, there are differences regarding the *C. burnetii* strains used for antigen preparation, the processes of strain production and purification, and the nature of the conjugate (see Table 2 for details). Because all three tests are based on the same biological principle, they are likely to be conditionally dependent, i.e. to simultaneously result in diagnostic errors (for instance if cross-reacting antibodies are present in the serum of an animal, or if only few antibodies are present).

**Table 2:** **Characteristics of the three ELISA tests used in the study**

Name used in the current study	Test 1	Test 2	Test 3
Commercial name	IDEXX Q fever Ab test	LSIVetTM Ruminant Q fever Serum^a^	ID.Vet ID Screen® Q fever indirect multi-species
Manufacturer	IDEXX	LSI Life Technologies^a^	IDvet^b^
Kit batches used in the current study	D401, E121	ELISACOXLS 020, ELISACOXLS 021, ELISACOXLS 023, ELISACOXLS 024	565 747
Strain used for antigen production	Isolated from *Dermacentor andersoni* ticks (Nine Mile reference strain)	Isolated from an ewe	Isolated from a cow
Conjugate	Secondary antibodies biding to ruminant IgG	Protein G (biding to IgG of diverse mammalian species)	Protein G (biding to IgG of diverse mammalian species)
Interpretation rules according to the manufacturer (ODR: optical density ratio)	ODR < 30% Negative 30% < ODR < 40% Doubtful 40% < ODR Positive	ODR < 40% Negative 40% < ODR < 100% Positive + 100% < ODR < 200% Positive + + 200% < ODR Positive + + +	ODR < 40% Negative 40% < ODR < 50% Doubtful 50% < ODR < 80% Positive 80% < ODR Strong positive

^a^Test 2 is currently commercialized by Themofisher Scientific under the commercial name PrioCHECK™ Ruminant Q Fever Ab Plate Kit. ^b^IDVet is now named Innovative Diagnostics.

The assays were performed within an ISO/IEC 17025 accredited quality control system. In particular, the reproducibility and trueness of the results were assessed by including, in each test, two internal positive reference serum samples that displayed results close to the respective positivity cut-offs set by the test manufacturers (SCE1/2011-12, ANSES, Sophia Antipolis) [34].

The results, expressed in optical density, were transformed into optical density ratios (ODRs) according to the manufacturers’ instructions following formula (1) for tests 1 and 2, and formula (2) for test 3.

1 $$ODR_{serum} = \frac{{OD_{serum} - OD_{NC} }}{{OD_{PC} - OD_{NC} }}$$

2 $$ODR_{serum} = \frac{{OD_{serum} }}{{OD_{PC} }}$$

where $ODR_{serum}$ is the optical density ratio of the tested serum, $OD_{serum}$ is the optical density of the tested serum, and $OD_{NC}$ and $OD_{PC}$ are the optical density of the negative and positive controls included in the test, respectively.

We interpreted the results as positive or negative according to the positivity threshold provided by the manufacturer and considering a serum positive when the manufacturer included a doubtful interpretation (tests 1 and 3, Table 2). The details of the cross-classified test results compiled by department in each species are provided in the Additional file 1: Appendix A.

### Statistical analysis

In this section, we describe the model according to the STARD BLCM guidelines [35]. The same model was independently run for each ruminant species. Computations were performed using JAGS software via the R package rjags [17] (a corresponding R script is provided in the.zip file of the Additional file 2).

**Figure 1:**
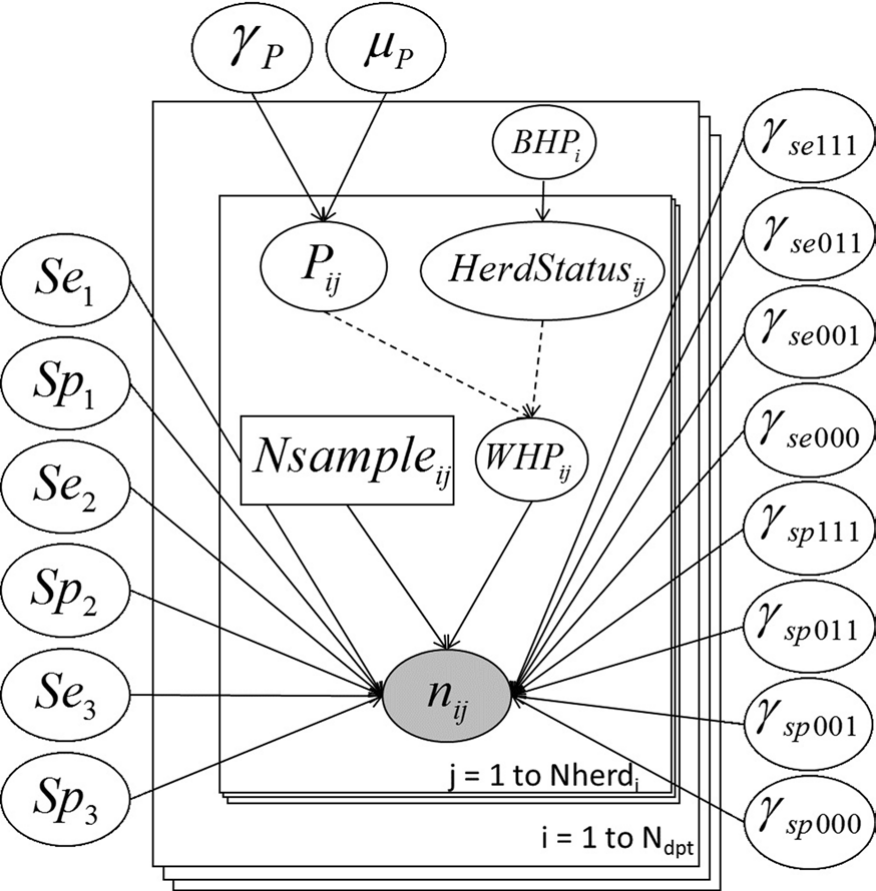
**Directed Acyclic Graph of the latent class model.** Every node is, if necessary, indexed by the department number ($i \in \left[ {1;N_{Dpt} } \right]$) and the herd number ($j \in \left[ {1;Nherd_{i} } \right]$). Plain arrows represent stochastic links and dotted arrows represent deterministic links. Observed data (grey oval) include $n_{ij}$, which is a vector of eight dimensions corresponding to the number of animals in each of the eight combinations of the three tests results. Measured covariables include $Nsample_{ij}$ the number of animals sampled in the $j^{th}$ herd of the $i^{th}$ department. Latent variables (white ovals) include the within-herd prevalence ($WHP_{ij}$), the herd latent status of each herd ($Herdstatus_{ij}$) and $P_{ij}$ the conditional true prevalence (in positive herds only). Unknown parameters (white ovals) include the Se and Sp values of the three ELISA tests, the conditional dependence terms (modelled according to Wang et al. [33]), the between-herd prevalence ($BHP_{i}$) in each department, and the hyper-parameters of the within-herd prevalence beta distribution ($\gamma_{P}$ and $\mu_{P}$).

We adapted the “three tests one population” LCM described by Wang et al. [33] to build a “three tests multiple populations” LCM, with each herd being considered a population. Thereby, we were able to model the variability of the within-herd seroprevalence ($WHP$) using a hierarchical zero-inflated beta-binomial distribution [36–38]. The directed acyclic graph of the model is presented in Figure 1.

### Latent status and hierarchical structure of the model

The individual latent status of an animal was defined as “the presence or absence of *C. burnetii*-specific antibodies in a serum sample”*,* later referred to as truly seropositive or truly seronegative. The within-herd seroprevalence, noted $WHP_{ij}$, was defined as the proportion of animals having a positive latent status (i.e. the proportion of truly seropositive animals) in the $j$ ^th^ herd from the $i$ th department. The herd latent status (noted $HerdStatus_{ij}$) is a binary variable defined as ‘negative’ (equal to zero) if the within-herd seroprevalence is null and as ‘positive’ (equal to 1) otherwise. The proportion of herds with a positive latent status in the i^th^ department was defined as the between-herd seroprevalence (noted $BHP_{i}$). We assumed that $WHP_{ij}$ followed a zero-inflated beta distribution conditionally to the herd status and to $P_{ij}$ the conditional true seroprevalence in positive herds [39–41]. We chose a zero-inflated beta distribution (Equation 3) to model the $WHP_{ij}$ to reflect that some herds were free of *C. burnetii* infection.

3 $$\begin{gathered} HerdStatus_{ij} \sim Bern\left( {BHP_{i} } \right)\\{ }P_{ij} \sim beta\left( {\mu_{P} \times \frac{{\left( {1 - \gamma_{P} } \right)}}{{\gamma_{P} }},\left( {1 - \mu_{P} } \right) \times \frac{{\left( {1 - \gamma_{P} } \right)}}{{\gamma_{P} }}} \right) \hfill \\ WHP_{ij} = HerdStatus_{ij} \times P_{ij} \hfill \\ \end{gathered}$$

The beta distribution of $P_{ij}$ was reparametrized in mean ($\mu_{P}$) and precision ($\gamma_{P}$) to facilitate the interpretation of its parameters [36–38]. In each herd, the beta distribution of the within-herd seroprevalence was truncated so that $P_{ij}$ was equal to or greater than the ratio 1/herd size.

#### Conditional dependence between the three tests

We applied the fixed effect model developed by Wang et al. [33], in which the conditional dependence between the three tests is considered with eight conditional dependence terms ($\gamma_{se111}$, $\gamma_{se001}$, $\gamma_{se001}$, $\gamma_{se000}$, $\gamma_{sp111}$, $\gamma_{sp011}$, $\gamma_{sp001}$ and $\gamma_{sp000}$). Each of these terms models the excess (or lack) of probability, compared to the expected probability under the assumption of conditional independence between tests, for a truly seropositive or seronegative individual to have a cross-classified test result in the corresponding category (for example “negative to test 1 while positive to tests 2 and 3” for $\gamma_{se011}$ and $\gamma_{sp011}$). The conditional dependence terms between each pair of tests was then calculated as in Equations 2 and 3 [33]:

4 $$\begin{gathered} \gamma_{{{\text{SpT1T2}}}} = \gamma_{{{\text{Sp111}}}} + \gamma_{{{\text{Sp110}}}} \hfill \\ \gamma_{{{\text{SpT1T3}}}} = \gamma_{{{\text{Sp111}}}} + \gamma_{{{\text{Sp101}}}} \hfill \\ \gamma_{{{\text{SpT2T3}}}} = \gamma_{{{\text{Sp111}}}} + \gamma_{{{\text{Sp011}}}} \hfill \\ \end{gathered}$$

5 $$\begin{gathered} \gamma_{{{\text{SeT1T2}}}} = \gamma_{{{\text{Se111}}}} + \gamma_{{{\text{Se110}}}} \hfill \\ \gamma_{{{\text{SeT1T3}}}} = \gamma_{{{\text{Se111}}}} + \gamma_{{{\text{Se101}}}} \hfill \\ \gamma_{{{\text{SeT2T3}}}} = \gamma_{{{\text{Se111}}}} + \gamma_{{{\text{Se011}}}} \hfill \\ \end{gathered}.$$

with $\gamma_{{{\text{SpT}}_{{\text{k}}} {\text{T}}_{l} }}$ and $\gamma_{{{\text{SeT}}_{k} {\text{T}}_{l} }}$ the pairwise conditional dependence terms between tests $k$ and $l$ for truly seronegative and truly seropositive animals, respectively.

#### Number of individuals in each category of the cross-classified test results

$n_{ij}$ is a vector of eight dimensions corresponding to the number of animals in each of the eight possible categories of cross-classified test results from the $j$ th herd in the $i$ th department. $n_{ij}$ follows a multinomial distribution of size $Nsample_{ij}$ (number of sampled animals in the herd) and whose probability depends on (1) the within-herd seroprevalence, (2) Se of the three tests, (3) Sp of the three tests, and (4) conditional dependence terms. To ensure that all conditional probabilities were included in the $\left[ {0;1} \right]$ interval, the same inequality constraints as proposed by Wang et al. [33] were implemented in JAGS with the D-interval distribution. With this aim, for each constrained parameter, a fictive observed variable was created to specify that these parameters were included in their respective definition intervals (the likelihood of the considered parameters is set to 0 otherwise, see JAGS user manual for details [17]). We also constrained Se estimates to be greater than the complement of the respective Sp estimates ($Se \in \left[ {1 - Sp;1} \right]$) to avoid unidentifiability issues related to the existence of a mirror image with the same likelihood (i.e., when the model switches the labels of truly positive and truly negative results) [28]. We provide the complete specifications of the model in the Additional file 1: Appendix B.

### Constant accuracy across populations

Because constant accuracy across all populations is one of the main hypotheses of the LCM, we checked that this assumption was fulfilled, considering independently each ruminant species. For cattle, this assumption was obviously not satisfied in all cattle herds: we observed 37 (23%) cows in the category $T_{1}^{ + } T_{2}^{ - } T_{3}^{ - }$(i.e., positive with test 1 and negative with the other tests) in the G department, while there were only 6 cows at most (0 to 3%) in this category in other departments (see Additional file 1: Appendix A for details); therefore, for cattle only, we made the choice to run the model on the one hand in all departments but G, and on the other in department G alone. Then, to check whether the assumption of constant accuracy across each population was valid for sheep and goats, and for cattle considering the nine remaining departments, we ran models independently in each department, using the posterior distributions of the complete model (i.e., the model considering departments altogether) as priors. These priors ensured that the models converged even in departments with a low within-herd variability (especially those in which most herds are either seropositive or seronegative, in which case Sp or Se, respectively are unidentifiable). Then, we graphically compared the Se and Sp estimates of the complete models with those obtained from the models considering each department separately.

### Prior distributions

Vaguely informative priors were assigned to each parameter allowing variation within a realistic range and forcing probability parameters ($\mu_{P}$, $\gamma_{P}$, ﻿$Se_{ \bullet }$, $Sp_{ \bullet }$ and $BHP_{i}$) ranging from 0 and 1 (Table 3). A Cauchy distribution, with 2.5 and 97.5 percentiles fixed at −0.5 and 0.5, respectively (calibrated with the SHELF package in R), was used as a prior for all conditional dependence terms in the model (Table 3). This prior supported posterior estimates with relatively low conditional dependence values (since the Cauchy distribution is centred and relatively peaked at 0), while also allowing the model to converge toward other posteriors when conditional terms were relatively high, since the Cauchy distribution has long distribution tails [42]. To assess the impact of choosing Cauchy prior distribution (instead of uniform prior distribution as suggested by Wang et al. [33]), we performed a sensitivity analysis by re-running the models with uniform distributions between −0.5 and 0.5 as the prior for all conditional dependence terms.

**Table 3:** **Prior distribution of unknown parameters**

**Nodes**	**Prior**
$\mu_{P}$**, **$\gamma_{P}$**, **$BHP_{i}$	${\text{beta}}\left( {0.5,0.5} \right)$
$Se_{1} ,\ Se_{2} ,\ Se_{3} ,\ Sp_{1} ,\ Sp_{2} ,\ Sp_{3}$	${\text{beta}}\left( {0.5,0.5} \right)$
$\gamma_{Se \bullet \bullet \bullet }$ $\gamma_{Sp \bullet \bullet \bullet }$	${\text{Cauchy}}\left( {0,0.039} \right)$

Monte Carlo Markov Chain (MCMC) techniques were used to estimate the full joint posterior distribution of parameters from prior distributions and data. Three independent MCMC chains were run in parallel. For each chain, 110 000 samples were produced: the first 10 000 were discarded as burn-in; the remaining 100 000 samples were thinned by selecting one out of 20 samples to deal with autocorrelation, thus retaining 5000 samples per chain. For each parameter, a point estimate was defined as the median of its marginal posterior distribution and a 95% credible interval was defined by the 2.5 and 97.5 percentiles of this marginal distribution. The convergence was checked again by displaying MCMC chain traces and autocorrelation plots, and by computing the Gelman and Rubin’s statistics, as modified by Brooks and Gelman [43]. To compare prior and posterior distributions, 15 000 samples of the full joint prior distributions were obtained for each species by running the model, considering that no animal was sampled. Prior vs posterior density plots and overlaps between both distributions were plotted and calculated with the MCMCvis R package [44].

Finally, we used the joint posterior distribution to represent the distribution of the within-herd seroprevalence in each species and to compute posterior distributions of herd sensitivities and herd specificities for each of the three tests. All calculations were performed on all the 15 000 joint MCMC samples from the posterior distribution, allowing us to obtain 15 000 estimations of all the parameters of interest.

### Accuracy of the model

The accuracy of the LCM when used to assess the respective Se and Sp of the three diagnostic tests, in a similar context to the current study, was checked by simulation. We defined five plausible scenarios considering various ranges of Se, Sp and conditional dependence values: 1) “original”, 2) “High Se, High Sp and conditional independence”, 3) “High Se, original Sp and low conditional dependence”, 4) “Low Se, original Sp and conditional dependence”, and 5) “Low Sp for test 3 only”. For each scenario and species, we generated 100 random datasets, which were analyzed by the LCM of the current study considering two alternative prior distributions for conditional dependence terms (either a $Cauchy\left( {0,0.039} \right)$ or a $Unif\left( { - 0.5,0.5} \right)$). We graphically assessed the accuracy of the Se and Sp estimators, and we calculated the mean bias, coverage probability, and square root of the quadratic error mean corresponding to all parameters obtained with both models, for each scenario and species. Details are provided in Additional file 1: Appendix C.

### Estimation of within-herd seroprevalence distribution

To represent the distribution of within-herd seroprevalence values in each species, we calculated the posterior distribution of 101 percentiles (from 0 to 1 by 0.01) from the corresponding beta distribution. Then, we plotted the cumulative distribution function and its 95% credibility band, by plotting the median and the 2.5 and 97.5 percentiles, respectively of the posterior distribution of each percentile.

### Herd sensitivities and specificities

Herd sensitivity (HSe) was defined as the probability that at least one animal is positive to the considered test in a seropositive herd, and herd specificity (HSp) as the probability that all animals are negative to the considered test in a seronegative herd. Details and justification about the procedure used to assess HSe and HSp are provided in Additional file 1: Appendix D. Briefly, we assumed that the number of animals positive to a test in a herd (noted $Npos_{herd}$) follows a binomial distribution of size $Nherd$ (the number of animals in the herd) and of probability ($WHP \times Se + \left( {1 - WHP} \right) \times \left( {1 - Sp} \right)$, where $WHP$ is the expected seroprevalence in the herd). Because WHP is generally not known in advance when preparing a sample plan, we calculated HSe and HSp values corresponding to a weighted integral of HSe and HSp values across the whole distribution of $WHP$ in each species. We assumed that the number of positive animals sampled ($Npos$) follows a hypergeometric distribution [45] of parameters $Nherd,Nsample,Npos_{herd}$, where $Npos_{herd}$ is the number of potential positive animals to the test in the herd, $Nsample$ is the number of animals sampled in the herd, and $Nherd$ is fixed as the median of the herd size (i.e., the total number of parous females) in each species (i.e., 57 for cattle, 120 for goat, 146 for sheep). We first calculated Hse and HSp for a sample size ranging from 1 to 20 animals sampled per herd and considering the herd positive if at least one animal tested positive. We then determined the best sample size for each test and species by maximizing the Youden Index (i.e., the sum of the Hse and HSp) for each sample size [46].

## Results

The point estimates and 95% credibility intervals of all parameters are available in Additional file 1: Appendix E). Trace plots, prior *vs* posterior density plots, and Gelman and Rubin’s statistics of all parameters for each species indicate the good convergence of the models and show that posteriors overlap with less than 30% of prior distributions for most of the parameters (see Additional file 2 for details).

### Validation of the hypothesis of constant accuracy across populations

The results of the models run independently in each department are available in Additional file 1: Appendix A. The Se and Sp estimates in all departments (except department G for cattle) were included within the 95% credibility intervals of the corresponding global model (including all departments but G). Therefore, we assumed that the hypothesis of constant accuracy across populations was valid when excluding cattle herds of department G.

### Sensitivity and specificity estimates at the individual level

**Figure 2:**
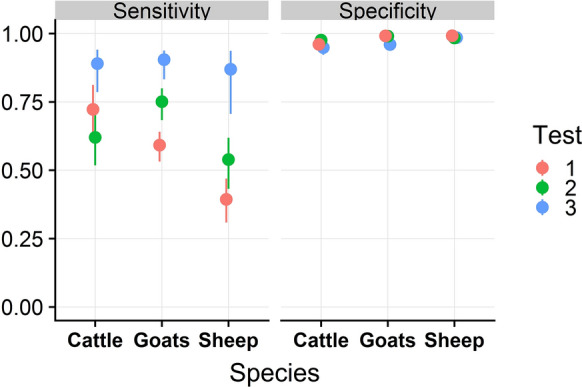
**Posterior estimates of the Se and Sp (according to the global model) for the three tests in each species.** Points represent point estimates and plain lines 95% credibility intervals. The Sp of test 1 in cattle is lower (0.750 [0.676; 0.860]) when considering only data from department G.

Se and Sp estimates for the three tests in each animal species according to the global model are shown in Table 4 and in Figure 2.

**Table 4:** **Se and Sp estimates of the three tests in each species with their 95% credible intervals (in square brackets) according to the global model**

Species	Test	Sensitivity Median [95% credible interval]	Specificity Median [95% credible interval]
Cattle	Test 1	0.720 [0.618; 0.808]	0.959 [0.942; 0.977]^a^
	Test 2	0.619 [0.517; 0.718]	0.975 [0.962; 0.987]
	Test 3	0.890 [0.785; 0.941]	0.948 [0.921; 0.978]
Goats	Test 1	0.592 [0.535; 0.641]	0.991 [0.982; 0.997]
	Test 2	0.752 [0.684; 0.799]	0.991 [0.981; 0.997]
	Test 3	0.905 [0.833; 0.938]	0.960 [0.937; 0.976]
Sheep	Test 1	0.393 [0.307; 0.470]	0.992 [0.985; 0.997]
	Test 2	0.538 [0.433; 0.618]	0.984 [0.974; 0.993]
	Test 3	0.869 [0.712; 0.936]	0.985 [0.973; 0.994]

^a^Sp of test 1 in cattle is lower (0.750 [0.676; 0.860]) when considering only data from department G.

According to the global model, the three tests were highly specific, with Sp estimates ranging from 94.8% [92.1; 97.8] for test 3 in cattle to 99.2% [98.5; 99.7] for test 1 in sheep. The Sp of test 1 was estimated at 75% [67.6; 86.0] in department G (according to the model run for department G only) and 95.9% [94.2; 97.7] in the other departments (according to the global model based on all departments but G; see Additional file 1: Appendix E for details). Conversely, Se estimates were low, especially for tests 1 and 2 in sheep, with Se values under or close to 50% (39.3% [30.7; 47.0] and 53.8% [43.3; 61.8], respectively). Test 3 was the most sensitive in all species but it was also the least specific. Test 2 was the most specific in cattle and was more sensitive than test 1 in goats and sheep. Test 1 was the most specific in sheep and was slightly more sensitive than test 2 in cattle.

**Figure 3:**
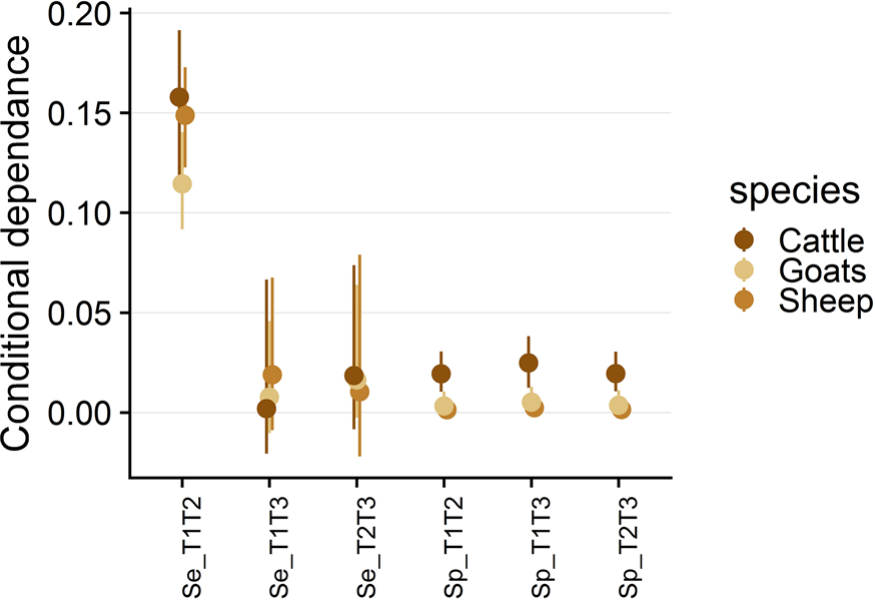
**Point estimates (dot) and 95% credibility intervals (lines) of the pairwise conditional dependence terms between each pair of tests in each species. **$\gamma_{{SeT_{k} T_{l} }}$ noted $Se\_TkTl$ for truly seropositive animals and $\gamma_{{SpT_{k} T_{l} }}$ noted $Sp\_TkTl$ for truly seronegative animals, $k,l \in \left\{ {1,2,3} \right\}$.

The conditional dependence estimates for truly seropositive and seronegative animals between each pair of tests are shown in Figure 3. Conditional dependence for truly seropositive animals between tests 1 and 2 was high in all species: in fact, the probability for a cow (a sheep or a goat, respectively) to be false-negative with test 1, knowing that it is already false-negative with test 2, varied from 72.3% (39.5% or 59%, respectively) under the assumption of conditional independence to 98% (67.5% or 74%, respectively) under the assumption of conditional dependence. Conversely, conditional dependence between tests 1 and 3 and between tests 2 and 3 was lower and 95% credibility intervals included zero. Conditional dependence for truly seronegative cattle, sheep and goats was low but strictly positive for all pairs of tests. Additionally, all 95% credibility intervals were strictly positive: for example, the probability for a goat to be false-positive with test 1, knowing that it is already false-positive with test 2 (or with test 3), varied from 0.9% under the assumption of conditional independence to 39.3% (or 14.3%) under the assumption of conditional dependence.

### Between- and within-herd seroprevalence accounting for diagnostic errors

**Figure 4:**
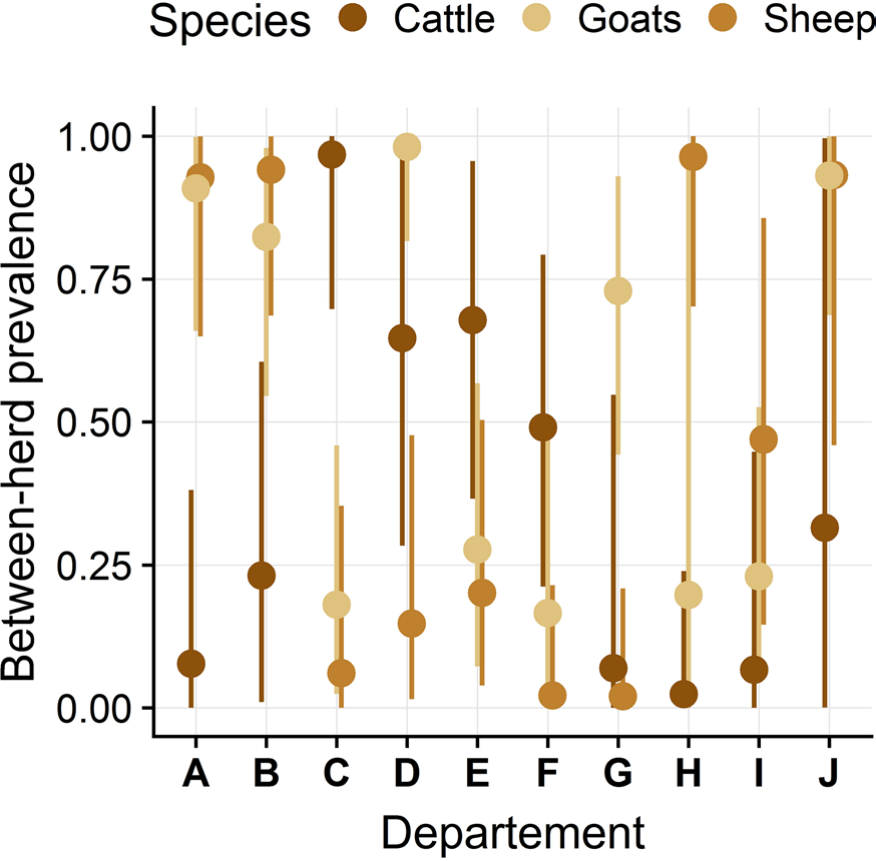
**Posterior estimates of the between-herd seroprevalence for each department and species.** Dots represent point estimates and plain lines their 95% credibility intervals. The between-herd seroprevalence shown for department G was estimated with the model ran only with herds from this department (see Additional file 1: Appendix E text for details).

The between-herd seroprevalence estimates varied among departments for all species (Figure 4): some departments had few seropositive herds whereas others had almost all herds seropositive for one or multiple species. Interestingly, in seven of the ten departments, between-herd seroprevalence estimates were in a similar range for sheep and goats, whereas they differed for cattle.

**Figure 5:**
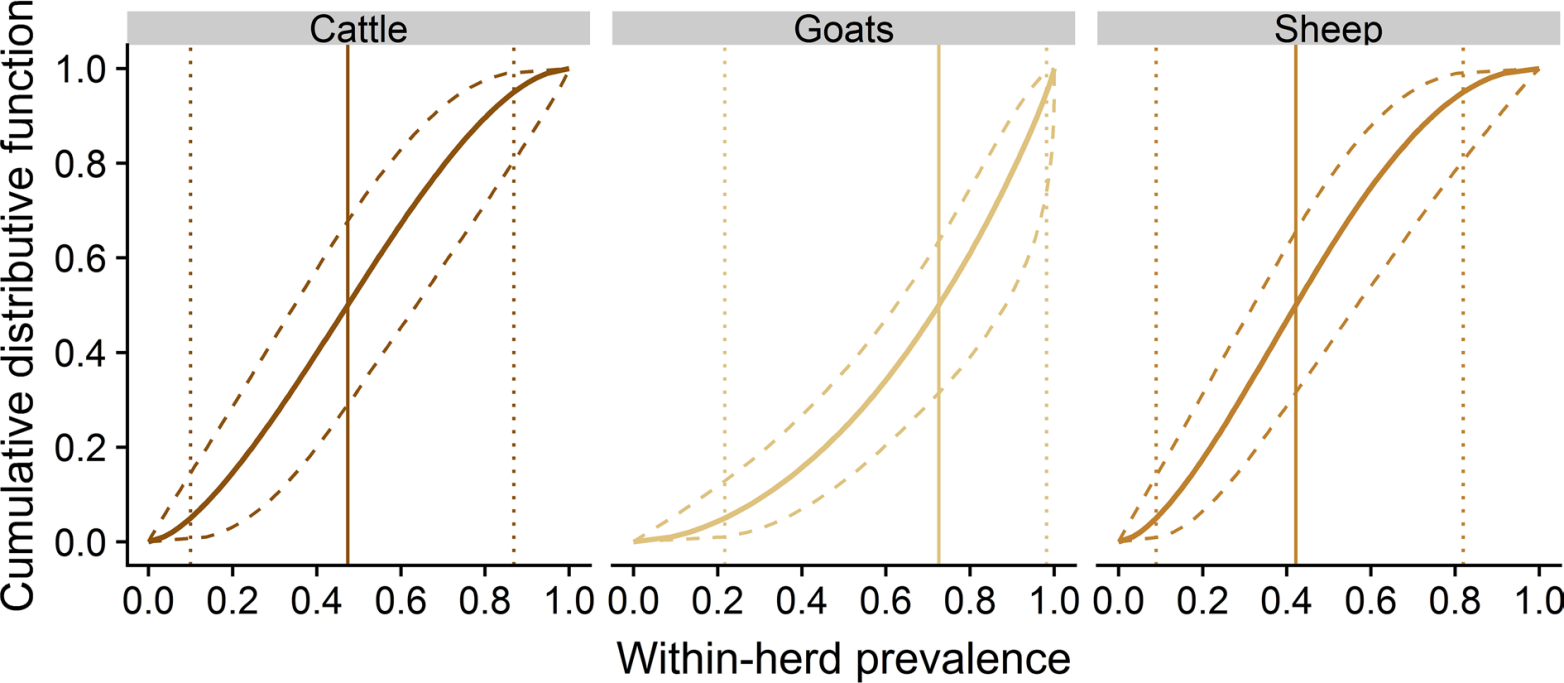
**Predicted cumulative distribution functions of the within-herd seroprevalence in seropositive herds for each species. Plain and dashed lines represent the median and the 95% credible intervals of the predicted quantiles, respectively.** Vertical plain and dotted lines highlight point estimates of the median, 5^th^ and 95^th^ percentiles of the predicted distribution of the within-herd seroprevalence.

The distributions of within-herd seroprevalence values (Figure 5) were wide in each species and tended to be higher in seropositive goat herds (with a median of 72.6% [62.1; 88.8]) than in cattle (47.3% [34.8; 63.7]) and sheep (41.9% [31.9; 56.1]) herds. The proportion of herds with a within-herd seroprevalence below 20% was also lower for goats (4.4% [0.7; 11.9]) than for cattle (14.5% [3.1; 28.8]) and for sheep (17.4% [6.3; 31.2]). Furthermore, 5% of seropositive herds had a seroprevalence below 21.0% [8.8; 36.2] for goats, while they had a seroprevalence below 10.0% [3.3; 23.7] for cattle, and below 9.0% [3.1; 18.3] for sheep. The proportion of herds displaying a high within-herd seroprevalence was also high for goats, as compared to the other species: 38.9% [25.2; 61.5] of seropositive goat herds had a within-herd seroprevalence above 80%, while this proportion was only 10.3% [2.4; 29.3] in cattle herds and 6.0% [1.0; 21.0] in sheep herds. Additionally, 95% of seropositive herds had a within-herd seroprevalence below 98% [93.8; 99.9] for goats, while it was below 86.7% [74.4%; 97.4] for cattle and 82% [69%; 94.5] for sheep.

### Optimal sample size accounting for herd sensitivity and specificity

**Figure 6:**
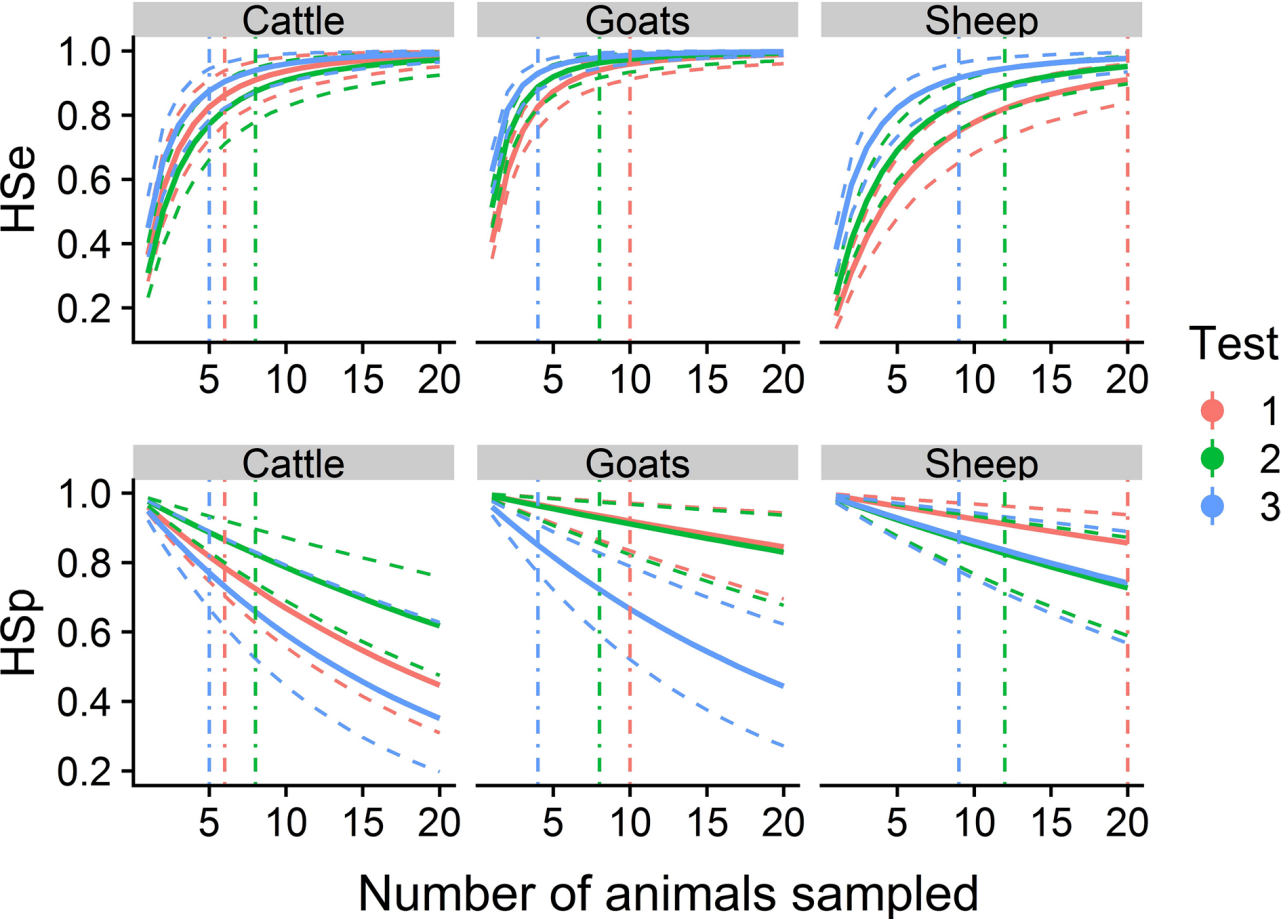
**Posterior estimates of the herd sensitivities (HSe: probability that at least one animal is positive to the test in a seropositive herd) and specificities (HSp: probability that all animals are negative to the test in a seronegative herd) of the three tests for each species.** The sample size per herd varies from 1 to 20 animals. HSe and HSp were integrated across the whole distribution of the within-herd seroprevalence in each species. Plain and dotted lines represent the median and the 95% credible interval of each parameter. Dot dashed vertical lines represent the optimal sample size (maximizing the Youden Index) for each test and species. All lines are colored in red, green and blue, respectively for tests 1, 2 and 3.

HSe values rapidly increased with the sample size, while HSp values decreased, as expected when considering a herd seropositive if at least one animal tests positive (Figure 6). Using a similar sample size, test 3 was the test with the highest HSe but also the lowest HSp. The optimal sample size for maximizing both HSe and HSp varied depending on the test and animal species considered (e.g., the point estimate was 3 animals in goat herds for test 3 and at least 20 animals in sheep herds for test 1).

### Accuracy of the model assessed by simulation

#### Comparison of Cauchy and uniform prior distributions

Point estimates of conditional dependence terms obtained with Cauchy prior distribution (hereafter named Cauchy model) were generally closer to the true value used to generate the datasets than point estimates obtained with uniform prior distribution (hereafter named uniform model) (Additional file 1: Figures C1, C2 and C3 of Appendix C). The Cauchy model presented a lower negative mean bias and a higher coverage probability than the uniform model for all Se and Sp estimators in cattle and goats for all scenarios (except scenario 4), and in sheep for scenarios 2 and 3 (Additional file 1: Table C2and C4 of Appendix C). The square roots of the quadratic error means of Se and Sp estimators of both models were low; they were lower for the Cauchy model in all scenarios (except scenario 4) in cattle and goats, and for scenarios 2 and 3 in sheep.

### Accuracy of the latent class model retained in the current study

Point estimates of the Se and Sp values assessed in cattle and goats were generally close and centered on the true values used to generate the datasets, while they were generally less than their respective true values in sheep (Additional file 1: Figures C1, C2 and C3 of Appendix C). The overall mean of the mean bias for Se and Sp estimators ranged between −0.0089 and −0.0028 across all scenarios for goats, between −0.0298 and −0.0102 for sheep, and between −0.0183 and −0.0025 for cattle (Additional file 1: Table C2 of Appendix C). The mean coverage probability considering all Se and Sp estimators ranged between 0.805 and 0.948 across all scenarios for goats, between 0.790 and 0.931 for sheep, and between 0.878 and 0.970 for cattle, respectively (Additional file 1: Table C3 of Appendix C). Although point estimates of Se in sheep seemed biased and might underestimate the Se of the test from −0.0113 to −0.0983, 95% credible intervals contained the true value used to generate the dataset in more than 90% of cases.

### Sensitivity analysis

Posterior estimates obtained when considering uniform prior distribution for conditional dependence terms (see Additional file 1: Appendix F) were very close to those obtained with Cauchy prior distribution, with a difference of less than 0.5% for Se estimates in sheep (less than 1.4% and 3.6% in goats and cattle respectively), and a difference of less than 0.1% for Sp estimates in sheep (less than 0.1% and 0.4% in goats and cattle respectively).

## Discussion

According to both the World Organisation for Animal Health (OIE) and EFSA, ELISA methods are the reference tests for the serological diagnosis of *C. burnetii* infection in animals [7, 47]. Nevertheless, in the absence of a gold standard, these tests are not well characterized and interpretation of their results at the individual and herd levels can be biased.

We used LCMs to assess the diagnostic performances of the three commonly used ELISA tests at the respective positivity or doubtful thresholds provided by their manufacturers. Our results allow for better interpretation of these tests and are essential to optimize their implementation as part of epidemiological studies.

Overall, when all hypotheses are verified, the estimation of diagnostic performances from LCMs is less biased than when it is based on an imperfect reference test or on samples of “known-status”. Importantly, using an imperfect gold standard leads to the assessment of relative Se and Sp values [18, 19, 35]. Moreover, unlike studies based on samples of “known-status”, LCMs can be used to analyze data collected from the target population in which the diagnostic tests are to be applied in practice (i.e., a population for which the true individual status remains unknown).

### Applicability of the data

The animals tested in this study constitute a subsample of a former survey, in which herds were randomly selected within ten French departments [5]. This subsample was not strictly random since we used the serum samples collected from the first 10 to 13 herds sampled by veterinarians in each department, which are 14.5%, 29.5% and 17.3% of the cattle, goat and sheep herds from the original survey, respectively. For this reason, a sampling bias could not be entirely ruled out: for instance, we may hypothesize that the earliest sampled herds were more likely to have reproductive disorders and/or to belong to farmers with a greater interest to search for *C. burnetii* infection (i.e. farmers with previous experience of Q fever abortions in their herd). Although the potential bias induced by this sampling procedure may have a minor impact on within- or between-herd seroprevalence estimates in each department, it is unlikely to impact the estimations of the tests’ diagnostic performances.

Given the origin of these data, the diagnostic performance estimates obtained in this study are essentially relevant to plan or analyze the results of epidemiological studies dealing with screening or active monitoring data. For the clinical diagnosis of Q fever, the current recommendations are to perform a group diagnosis (at the herd scale) and to use serology as a complement to PCR direct diagnosis [7, 47]. However, our data were not specifically collected from herds with reproductive disorders such as series of abortions (i.e., herds with a clinical suspicion of Q fever), and we cannot ascertain whether diagnostic performances of the three ELISA tests are similar in this context. For example, in herds suspected of clinical Q fever, Se might be higher if most animals that suffer from active infection display high antibody titers or, conversely, lower if they have not yet seroconverted. Therefore, the Se and Sp values estimated in our study are a priori not optimal for use when the tests are performed for diagnostic purposes in an abortive context.

### Definition of the latent status

The latent status is unobserved. It is only assessed by the model as a computer-based consensus between tests and it must be cautiously interpreted. The LCMs were run with data resulting from three ELISA tests that all detect circulating antibodies against *C. burnetii*. Thus, we assumed that the latent status modelled corresponds to “the presence or absence of *C. burnetii*-specific antibodies in a serum sample”: the Se and Sp estimates therefore correspond to the diagnostic performances of these ELISA tests to detect truly seropositive or truly seronegative animals. As previously mentioned in the paragraph about the applicability of the data, the latent status modelled does not (and cannot be extrapolated to) identify animals vaccinated against Q fever or establishing animal freedom from infection or defining *C. burnetii* shedding status or attributing abortions to *C. burnetii.* Clearly, defining a *C. burnetii* infection status is complex and requires specific sampling strategies that need to be interpreted in the context of each herd history (abortions, shedding, vaccination, movement and introduction, etc.) [7].

### Constant accuracy across populations

The hypothesis of constant accuracy across the populations was overall satisfied at the scale of the department, except for test 1 performed in cattle sampled from department G. Therefore, we considered that the variations of diagnostic performances across populations were negligible, excluding cattle herds from department G.

The proportion of cows positive to test 1 and negative to tests 2 and 3 was higher in department G. The model considered these cows as false-positive due to lower Sp of test 1 in cattle in this area (Additional file 1: Appendix E). This result is consistent with the fact that abortions were rarely attributed to *C. burnetii* in this department for at least the past decade, according to the quantitative PCR results obtained by the veterinary laboratory responsible for the diagnosis of abortions in this area. These false-positive cows were sampled from several herds (9 out of the 10 herds sampled in this department). We were not able to identify any specific factor (i.e., age, parity, geographic area) associated with this result. We also discarded the hypothesis of a technical issue at the laboratories because positive and negative controls of each ELISA fulfil the validity criteria defined by the manufacturer. We also discarded a batch effect of test 1: although all cattle from department G were tested with a same batch of test 1, this batch was also used for other species and departments. Overall, these results point out a potential lack of Sp of test 1 in some epidemiological contexts, at least when applied to cattle. This lack of Sp might be related to the antigen preparation of this test, based on the tick-isolated Nine Mile reference strain: we may hypothesize that this antigen cross-reacts with antibodies against other bacterial species [48–50], especially some *Coxiella*-like bacteria [51, 52]; however, data are lacking to assess whether cattle are particularly exposed to *Coxiella*-like bacteria in this department. Overall, we conclude that test 1 should be used and interpreted carefully in cattle because false-positive results may occur, at least in some geographic areas.

### Sensitivity and specificity estimates

According to our global models (cattle from department G being excluded), the three tests were highly specific, especially for goats and sheep, but poorly sensitive, with Se estimates particularly low (they were below 76% for tests 1 and 2 in all species). This result supports the current recommendations of the OIE that ELISA tests should not be used for the diagnosis of *C. burnetii* infection at the individual level [7]. Although test 3 was the most sensitive in all species, it was also the least specific in cattle and goats. The variation in Se and Sp estimates between domestic ruminant species (Figure 2) is noteworthy, and was already outlined in previous studies [14, 20, 24]. This result points out the importance of considering the species on which a test is intended to be applied when choosing an ELISA test.

The Sp and Se estimates of the current study are respectively similar and lower than those reported in previous studies [20–24]. We explain this difference by the fact that our models provide accurate estimations of the conditional dependence between the three ELISA tests. In particular, the conditional dependence for seropositive animals between tests 1 and 2 was greater than 0.1 in all species, which is high compared to the value estimated in other studies, in which conditional dependence was either omitted (and fixed to 0) [20, 24] or estimated at low values [21–24] (0.004 to 0.013 according to Lucchese et al. for the same tests 1 and 2 [24]). The high positive conditional dependence between tests 1 and 2 in all species for truly seropositive animals results from a large increase of the probability of mutual false-negative results between tests 1 and 2, compared to the situation where tests are conditionally independent. The estimates of the conditional dependence for seronegative animals between all pairs of tests were low but strictly positive. As a result, even though false-positive animals are rare (because specificities of the tests are high), the probability that an animal would be falsely positive to a test, given that it is already false-positive to another test, is relatively high. Therefore, when suspecting one serum to be falsely positive to one of the three ELISA tests, the procedure for testing this serum with another ELISA test will generally also provide a positive result, even if the animal is truly seronegative.

Our main hypothesis to explain discrepancies in diagnostic performance estimates between the current and previous studies was that our study considered simultaneously all three ELISA tests and their conditional dependence. As a consequence, the models from previous studies might be adjusted to a latent status that is different from the one in our study (i.e., “the presence or absence of *C. burnetii*-specific antibodies in a serum sample according to the combination of tests 1, 2 and 3”) [53]. In previous studies, only test 1 [22–24] and/or test 2 [21, 24] were investigated, sometimes in comparison with CFT [24] or IFA [22, 23]. Because Paul et al. [21] used the same ELISA test (test 2) on two different biological samples (milk and serum), their modelled latent status was likely “being seropositive with test 2”. Regarding the studies of Muleme et al. [22], Lucchese et al. [24] and Wood et al. [23], the modelled latent status may stand for “being seropositive with test 1 and/or test 2”, which is different from being seropositive with the combination of tests 1, 2 and 3. In our study, tests 1 and 2 displayed the highest pairwise conditional dependence for seropositive animals (Figure 3). Therefore, tests 1 and 2 tend towards the same results in truly seropositive individuals and, for example, if one individual is false-negative (or true-positive) with test 1, it will probably also be false-negative (or true-positive) with test 2. Moreover, because in our study these tests are less sensitive and slightly more specific than test 3, it is possible that these two tests detect only individuals displaying levels of antibodies higher than those detected with test 3; if this assumption is correct, the differences between the Se values estimated in our and other studies may be explained by the fact that the latent status modelled involuntary differed, being “displaying a high level of antibodies against *C. burnetii*” in previous studies [22–24] against “displaying both low and high levels of antibody against C*. burnetii*” in the current study.

The assessment of conditional dependence between tests is a complex issue in LCMs [31, 32]. In this study, the results of simulations (Additional file 1: Appendix C) show that our model accurately estimated conditional dependence between tests. We hypothesize that we succeeded in estimating high conditional dependence estimates because test 3 was more sensitive and almost as specific as the other two tests, which allowed us to consider that some individuals, negative with both tests 1 and 2 but positive with test 3, were actually truly seropositive. An alternative might be that test 3 was poorly specific and that the estimates of the current study were biased by the inclusion of a non-specific test; however, if test 3 really lacked Sp, we would expect to observe only few herds with no animal positive to test 3, which was not supported by our data (130 herds among the 308 included in the study were entirely negative with test 3). Moreover, the simulation of scenario 5 demonstrated that the inclusion of a less specific test does not bias significantly the posterior estimates of our model (Additional file 1: Appendix C).

### Between- and within-herd seroprevalence estimates

Between-herd seroprevalence estimates were highly variable among geographic areas (Figure 5), with wide credible intervals due to the small number of herds considered (*n* = 9 to 13 per department). Between-herd seroprevalence was estimated to be greater than 75% in four departments for goat and sheep herds, and in one department for cattle herds. Conversely, less than 25% of herds were seropositive in five departments for cattle and sheep herds, and in four departments for goat herds. The estimates of the current study are overall similar to those of the original survey [5], except that the values are lower (respectively higher) in departments with low (respectively high) between-herd seroprevalence in the original survey. We explain the higher number of seropositive herds in endemic departments found in our study by the fact that the study of Gache et al. [5] was based only on test 2, which is less sensitive than test 3 according to our model; and we explain the detection of fewer false-positive herds in departments where *C. burnetii* infection is rare by the fact that we modelled the imperfect Sp of the tests. Interestingly, similar seroprevalence values were observed in sheep and goat herds in several departments; in addition to the fact that *C. burnetii* strains circulating in sheep and goats present genotypic similarities [54], this observation supports the hypothesis that *C. burnetii* is more frequently transmitted between sheep and goat herds than between cattle and small ruminants herds.

Regarding within-herd seroprevalence, the ranges of the estimates were wide in all species, some herds having a low proportion of seropositive animals, while others had a high proportion. As expected, since the original survey was based on test 2, which is less sensitive than test 3, the within-herd seroprevalence was overall higher in our study than in the original survey [5]. Moreover, both studies revealed greater within-herd seroprevalence levels in goat herds compared to cattle and sheep herds; these differences are consistent with more active bacterial circulation in goat herds due to the massive shedding of *C. burnetii* and the higher rate of abortions attributed to *C. burnetii* reported in this species [55–58].

### Herd sensitivity and specificity

We chose to calculate HSe and HSp by weighting them across the whole distribution of within-herd seroprevalence in each species, instead of fixing an arbitrary within-herd seroprevalence value (such as the mean or median of its distribution). In this way, estimated values of HSe and HSp are more representative of field situations. Clearly, within-herd seroprevalence is generally not known in advance when preparing a sample plan. Moreover, we calculated HSe and HSp with the aim of detecting all truly seropositive herds, including those with low within-herd seroprevalence (i.e. a herd is considered truly seropositive if at least one parous female is truly seropositive out of the total number of parous females present in the herd). We assume that this target stands for the question “is the herd free of *C. burnetii* or not?”, but it might not be relevant for diagnostic purposes in an abortive context. Indeed, the targeted seroprevalence required to attribute abortion waves to *C. burnetii* is generally higher, as recommended by EFSA in complement to quantitative PCR [47].

We chose to consider that a herd was seropositive if at least one parous female tested positive, and we assessed the optimal sample size to maximize both HSe and HSp for every test and species. Despite low individual Se, HSe rapidly increased with the number of animals tested for all three ELISA tests (Figure 6). This result shows that, when a herd is considered seropositive as soon as one animal tests positive, the lack of Se of a test becomes unproblematic at the herd level, if several animals are tested. Conversely, HSp rapidly decreased with the number of animals tested; thus, moderate Sp differences at the individual level are amplified at the herd level. Therefore, when establishing a sampling scheme, a compromise is needed between sampling many animals (in order to increase the probability of sampling truly seropositive animals) and sampling few animals (in order to avoid false-positive results in truly seronegative herds). Overall, we recommend considering an optimal sample size, maximizing both HSe and HSp. This sample size varies depending on the considered test and ruminant species: in practice, the choice of an optimal test and sampling strategy should be made after a cost-effectiveness analysis depending on the aim of the screening (e.g., epidemiology, animal introduction, herd status, etc.) and the cost of sampling and testing any supplemental animal.

### Validity of the model

The model developed by Wang et al. in 2017 [33] was adapted for the purpose of this study because it allowed for modelling conditional dependence among multiple tests based on a same biological process. The major adjustment of the model concerned the hierarchical structure of the $WHP$ distribution (i.e. a zero-inflated beta-binomial distribution) which allowed us to account for the presence of herds free of seropositive animals [39–41]. This hierarchical structure associated with LCMs was shown to be accurate to assess between-herd seroprevalence in the case of high between-herd seroprevalence and Se values [59], which is the case for at least one of the tests and some departments in the current study.

#### Identifiability of the model

For each investigated ruminant species, we independently applied a LCM considering three different serological tests performed on numerous populations (each of the 106 cattle herds, 103 goat herds, and 99 sheep herds were considered a distinct population). The LCMs included 28 parameters, i.e., the Se and Sp of the three tests, the eight conditional dependence terms, the ten between-herd seroprevalence terms, and the two hyper-parameters of the within-herd distributions. For LCMs based on three tests, there are seven degrees of freedom for each population, corresponding to the possible number of categories of the cross-classified test results minus one [60]. Our model was therefore theoretically identifiable if at least four populations with distinct seroprevalence levels were included in the dataset. Based on apparent between- and within-herd seroprevalence levels reported in the original survey [5], we hypothesized that many herds (apart from seronegative herds for which seroprevalence is nil) would display distinct and contrasted seroprevalence levels. Accordingly, within-herd seroprevalence estimates modeled in all three species displayed wide ranges, which means that the number of populations with distinct seroprevalence levels was much greater than four. Thus, the number of degrees of freedom of our dataset was greater than the minimum required for model identifiability. In addition, the convergence of all LCMs met the expectations (see trace plots and Gelman and Rubin’s statistics in the Additional file 2).

#### Importance of the prior

Because the model was identifiable and the dataset of high quality, we used non-informative prior distributions for all parameters of the model (Table 3). We chose a $beta\left( {0.5,0.5} \right)$ distribution for all probability parameters [61], and a Cauchy distribution of location 0 and scale 0.039 for conditional dependence terms (chosen so that the 2.5 and 97.5 percentiles were fixed at −0.5 and 0.5, respectively). The choice of Cauchy prior distribution was motivated by the fact that, based on the authors’ experience, the use of a uniform distribution for conditional dependence terms (as suggested by Wang et al. [33]) generally favors posterior estimates with relatively high conditional dependence values, even when tests are conditionally independent. In contrast, Cauchy prior distribution, which was recommended for use as a default prior distribution for various models [42, 62], peaks at its location and has long tail ends. Therefore, it allows for occasional large coefficients while still performing a reasonable amount of shrinkage for coefficients near zero [42]. As a result, in our case, Cauchy prior distribution favors posterior estimates with relatively low conditional dependence terms, while it allows the convergence of the models toward high conditional dependence terms if suggested by the data.

The simulations performed in this study confirmed that Cauchy prior distribution outperforms uniform distribution for conditional dependence terms because it leads to lower negative bias, higher coverage probability, and lower quadratic error mean in all tested scenarios, except those with very low Se values (scenario 4 in cattle and goats, and scenarios 1, 4 and 5 in sheep, see Additional file 1: Appendix C for details). Overall, thanks to the high number of animals included in the current study, the impact of prior distributions used for conditional dependence terms was limited, as shown by the similarity of posterior estimates obtained with uniform or Cauchy prior distributions (see results of the sensitivity analysis in Additional file 1: Appendix F). Finally, the weak overlaps between posterior and prior distributions confirmed that posterior estimates of the current study are mostly obtained from the information included in the dataset (see output of the model in the Additional file 2).

#### Accuracy of the model

The results of the simulations (Additional file 1: Appendix C) show that the mean negative bias regarding Se and Sp estimators of our model is less than −0.01 in goats, −0.02 in cattle and −0.03 in sheep. The best performances estimated with our model for goats might be attributed to higher levels of within-herd seroprevalence in seropositive goat herds compared to sheep and cattle herds, [with a median of the within herd seroprevalence distribution estimated at 72.6% for goats and 41.9% and 47.3% for sheep and cattle, respectively (Figure 5)]. The highest negative bias on the Se estimators for sheep might be attributed both to lower levels of within-herd seroprevalence in seropositive sheep herds compared to goat herds and to a lower Se of tests 1 and 2 in this species. Accordingly, in cattle, while Se estimators of scenarios 1, 2, 3 and 5 show a low negative bias, scenario 4 (the “low Se”) is the most negatively biased.

In conclusion, when datasets were generated according to the main assumption of the model, posterior estimates provided accurate estimations of the true sensitivities and specificities for cattle and goats, even when the scenarios used to generate the datasets included conditional dependence values that were lower or higher than in reality. In sheep, Se values might be slightly underestimated but the coverage probability of their 95% credible intervals remained above 91%, indicating the importance of considering the whole 95% credible interval when interpreting the results of this study in sheep.

This study is the first to simultaneously assess the diagnostic performances of the three main ELISA tests commercialized for the serological diagnosis of *C. burnetii* infection in domestic ruminants. Our results point out that these tests are less sensitive than estimated in previous studies. The hierarchical beta-binomial LCM developed for the current study may be applied in the future to more than three tests, or to other diseases.

## Supplementary Information

**Additional file 1.  Supplementary material.** **Appendix A.** Cross-classified test results in each department and verification of the hypothesis of constant accuracy across populations. **Appendix B.** Complete specifications of the model. **Appendix C.** Simulation study. **Appendix D.** Herd sensitivity, herd specificity. **Appendix E.** Estimations of the model. **Appendix F.** Sensitivity analysis.

**Additional file 2: Output of the model.** R-Markdown Word document containing R code, summary of the models, Gelman and rubin’s statistics, trace plots and prior vs posterior density plots.

**Additional file 3: Data.** (Data_Lurier_LCM_Cburnetii_VetRes_2021). Cross-classified test results of the three ELISA in each included herd.

**Additional file 4: Rscript.** Runnable version of the R script for the latent class models.

## Data Availability

Appendix A, B, C, D and E can be found in the Additional file 1. Data (with anonymized numbers for herds and letters for departments) and a runnable R script of the models can be found in Additional files 2 and 3 respectively.

## Abbreviations

BDNI: French National Cattle Identification Database
BHP: Between-herd seroprevalence
CFT: Complement fixation test
EFSA: European Food Safety Authority
HSe: Herd sensitivity
HSp: Herd specificity
IgG: Immunoglobulin G
IFA: Indirect immunofluorescence assay
LCM: Latent class model
MCMC: Monte Carlo Markov Chain
NRL: National reference laboratory
ODR: Optical density ratio
OIE: World Organisation for Animal Health
PCR: Polymerase Chain Reaction
WHP: Within-herd seroprevalence
