# Supplementary material for: Evaluation using latent class models of the diagnostic performances of three ELISA tests commercialized for the serological diagnosis of Coxiella burnetii infection in domestic ruminants
Source: Vet Res. 2021 Apr 14;52:56. doi: 10.1186/s13567-021-00926-w (PMC8048088; doi:10.1186/s13567-021-00926-w)
Supplement: Supplementary file 1 — Additional file 1. Supplementary material. Appendix A. Cross-classified test results in each department and verification of the hypothesis of constant accuracy across populations. Appendix B. Complete specifications of the model. Appendix C. Simulation study. Appendix D. Herd sensitivity, herd specificity. Appendix E. Estimations of the model. Appendix F. Sensitivity analysis. [file 13567_2021_926_MOESM1_ESM.docx]

**Additional file 1 Supplementary material**

**Evaluation using latent class models of the diagnostic performances of three ELISA tests commercialized for the serological diagnosis of *Coxiella burnetii* infection in domestic ruminants**

Thibaut Lurier^1, 2,3^*, Elodie Rousset^4^, Patrick Gasqui^1^, Carole Sala^5^, Clément Claustre^1^, David Abrial^1^, Philippe Dufour^4^, Renée de Crémoux^6^, Kristel Gache^7^, Marie Laure Delignette-Muller^8^, Florence Ayral^2^, Elsa Jourdain^1^

1. Université Clermont Auvergne, INRAE, VetAgro Sup, UMR EPIA, 63122 Saint-Genès-Champanelle, France
2. Université de Lyon, INRAE, VetAgro Sup, UMR EPIA, 69280 Marcy l’Etoile, France
3. Université de Lyon, INRAE, VetAgro Sup, Usc 1233 UR RS2GP, 69280 Marcy l’Etoile, France
4. ANSES, Sophia Antipolis Laboratory, Animal Q fever Unit, Sophia Antipolis, France
5. University of Lyon-ANSES Lyon, Epidemiology and Support to Surveillance Unit, French Agency for Food, Environmental and Occupational Health & Safety (ANSES), Lyon, France
6. French Livestock Institute, Ruminant Health Management Joint Unit, Paris, France
7. GDS France (National Animal Health Farmers’ Organization), Paris, France
8. Université de Lyon, Université Lyon 1, VetAgro Sup, CNRS, UMR 5558, Laboratoire de Biométrie et Biologie Evolutive, 69622 Villeurbanne, France

*Corresponding author: [thibaut.lurier@vetagro-sup.fr](mailto:thibaut.lurier@vetagro-sup.fr)

**Appendix A. Cross-classified test results in each department and verification of the hypothesis of constant accuracy across populations**

Tables A1, A2 and A3 present the number of cattle, goats and sheep, respectively in each of the eight categories of cross-classified test results for each department. The variations observed in the number of animals in each category and department seem compatible with variations in seroprevalence in each department, except for the number of cattle in the category $T_{1}^{+}T_{2}^{-}T_{3}^{-}$ in department G. There were 37 cattle positive to test 1 and negative to tests 2 and 3, whereas there were only a few animals in this category in the other departments and species. This observation led us to question the assumption of constant accuracy of tests in each population, particularly for test 1 in cattle. Therefore, for cattle, we ran the model in department G and in the 9 other departments independently.

**Table A1: Number of cattle in each of the eight categories of cross-classified test results in each department.**

| **Cattle** | | | | | | | | | |
| --- | --- | --- | --- | --- | --- | --- | --- | --- | --- |
| dpt | $T_{1}^{-}T_{2}^{-}T_{3}^{-}$ | $T_{1}^{-}T_{2}^{-}T_{3}^{+}$ | $T_{1}^{-}T_{2}^{+}T_{3}^{-}$ | $T_{1}^{-}T_{2}^{+}T_{3}^{+}$ | $T_{1}^{+}T_{2}^{-}T_{3}^{-}$ | $T_{1}^{+}T_{2}^{-}T_{3}^{+}$ | $T_{1}^{+}T_{2}^{+}T_{3}^{-}$ | $T_{1}^{+}T_{2}^{+}T_{3}^{+}$ | Total |
| A | 134 | 4 | 0 | 0 | 1 | 1 | 0 | 3 | 143 |
| B | 135 | 12 | 0 | 1 | 5 | 2 | 1 | 1 | 157 |
| C | 80 | 21 | 0 | 0 | 1 | 7 | 1 | 40 | 150 |
| D | 130 | 6 | 0 | 1 | 1 | 3 | 1 | 39 | 181 |
| E | 89 | 15 | 0 | 0 | 6 | 8 | 6 | 31 | 155 |
| F | 111 | 13 | 0 | 0 | 0 | 3 | 1 | 33 | 161 |
| G | 114 | 2 | 0 | 0 | 37 | 1 | 0 | 1 | 155 |
| H | 143 | 2 | 0 | 1 | 0 | 0 | 0 | 4 | 150 |
| I | 138 | 5 | 0 | 0 | 2 | 3 | 1 | 3 | 152 |
| J | 8 | 0 | 0 | 0 | 0 | 0 | 0 | 1 | 9 |

**Table A2: Number of goats in each of the eight categories of cross-classified test results in each department.**

| **Goats** | | | | | | | | | |
| --- | --- | --- | --- | --- | --- | --- | --- | --- | --- |
| dpt | $T_{1}^{-}T_{2}^{-}T_{3}^{-}$ | $T_{1}^{-}T_{2}^{-}T_{3}^{+}$ | $T_{1}^{-}T_{2}^{+}T_{3}^{-}$ | $T_{1}^{-}T_{2}^{+}T_{3}^{+}$ | $T_{1}^{+}T_{2}^{-}T_{3}^{-}$ | $T_{1}^{+}T_{2}^{-}T_{3}^{+}$ | $T_{1}^{+}T_{2}^{+}T_{3}^{-}$ | $T_{1}^{+}T_{2}^{+}T_{3}^{+}$ | Total |
| A | 63 | 14 | 1 | 13 | 1 | 0 | 3 | 59 | 154 |
| B | 68 | 28 | 2 | 13 | 2 | 1 | 3 | 44 | 161 |
| C | 181 | 15 | 0 | 0 | 0 | 3 | 0 | 2 | 201 |
| D | 42 | 9 | 3 | 25 | 1 | 3 | 4 | 88 | 175 |
| E | 123 | 6 | 0 | 7 | 1 | 3 | 0 | 12 | 152 |
| F | 114 | 10 | 0 | 0 | 2 | 0 | 1 | 7 | 134 |
| G | 68 | 22 | 5 | 15 | 0 | 0 | 3 | 33 | 146 |
| H | 9 | 1 | 1 | 0 | 0 | 0 | 0 | 0 | 11 |
| I | 132 | 13 | 0 | 4 | 0 | 0 | 0 | 4 | 153 |
| J | 72 | 24 | 3 | 20 | 2 | 1 | 4 | 61 | 187 |

**Table A3: Number of sheep in each of the eight categories of cross-classified test results in each department.**

| **Sheep** | | | | | | | | | |
| --- | --- | --- | --- | --- | --- | --- | --- | --- | --- |
| dpt | $T_{1}^{-}T_{2}^{-}T_{3}^{-}$ | $T_{1}^{-}T_{2}^{-}T_{3}^{+}$ | $T_{1}^{-}T_{2}^{+}T_{3}^{-}$ | $T_{1}^{-}T_{2}^{+}T_{3}^{+}$ | $T_{1}^{+}T_{2}^{-}T_{3}^{-}$ | $T_{1}^{+}T_{2}^{-}T_{3}^{+}$ | $T_{1}^{+}T_{2}^{+}T_{3}^{-}$ | $T_{1}^{+}T_{2}^{+}T_{3}^{+}$ | Total |
| A | 91 | 27 | 0 | 10 | 2 | 0 | 0 | 35 | 165 |
| B | 94 | 28 | 5 | 11 | 0 | 0 | 2 | 22 | 162 |
| C | 145 | 3 | 0 | 0 | 1 | 0 | 0 | 0 | 149 |
| D | 124 | 2 | 8 | 4 | 0 | 0 | 0 | 7 | 145 |
| E | 142 | 10 | 0 | 0 | 0 | 0 | 0 | 3 | 155 |
| F | 154 | 1 | 1 | 0 | 1 | 0 | 0 | 0 | 157 |
| G | 161 | 0 | 0 | 0 | 0 | 0 | 0 | 0 | 161 |
| H | 91 | 25 | 3 | 4 | 3 | 2 | 0 | 18 | 146 |
| I | 124 | 15 | 9 | 3 | 2 | 1 | 0 | 2 | 156 |
| J | 24 | 2 | 1 | 0 | 0 | 0 | 0 | 9 | 36 |

To check whether the assumption of constant accuracy across each population remains valid in sheep and goats, and in the 9 other departments for cattle, we run the model independently in each department taking the posterior distribution of the complete model as a prior for the analysis. Including posterior distribution as a prior at this step ensured that the model converged, even in departments with low within-herd variability (especially in departments where most herds are seropositive or seronegative). Figures A1 and A2 present the posterior estimate of sensitivities and specificities, respectively in each department.


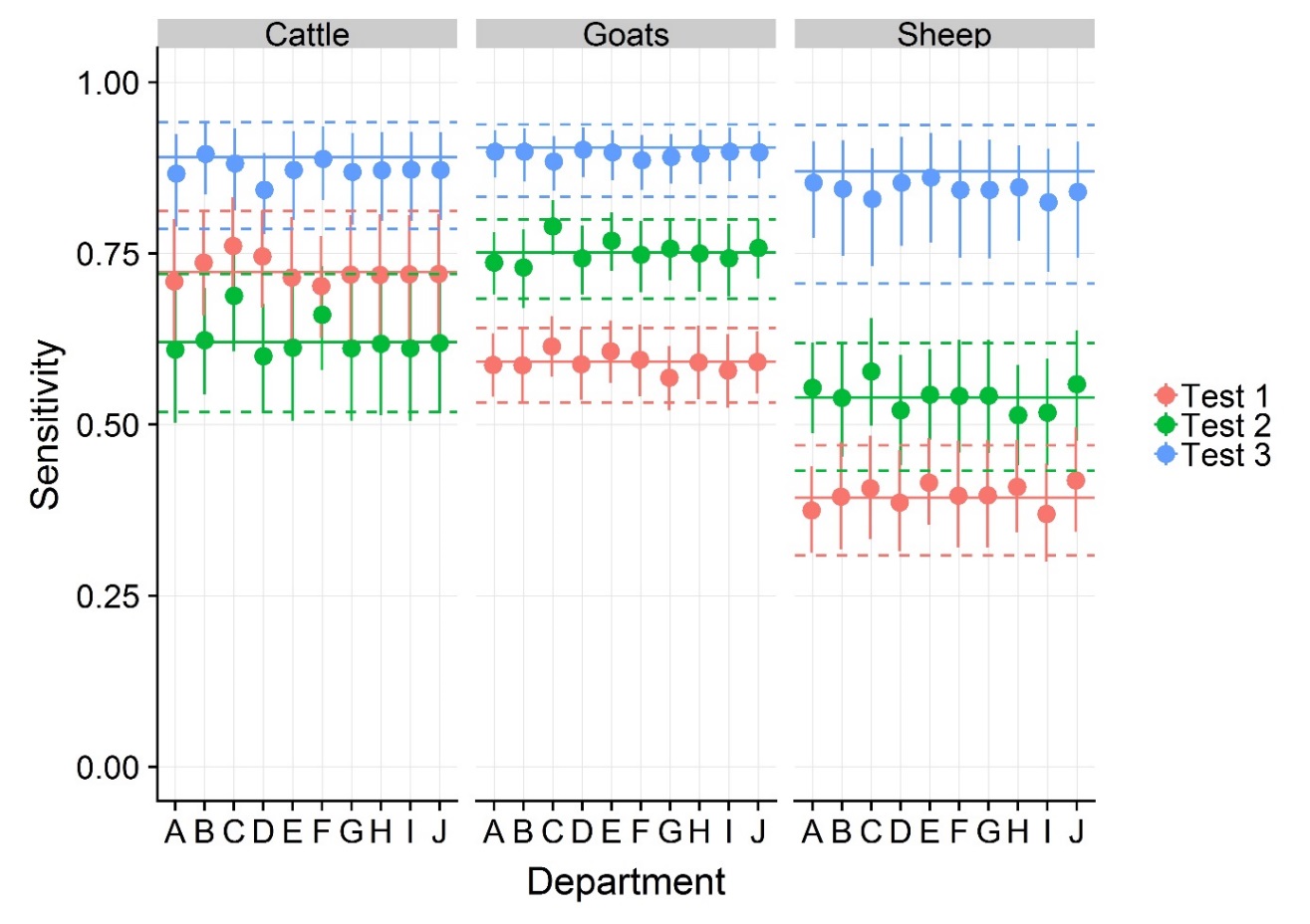


**Figure A1: Posterior estimates of the sensitivities in each department and in each species**. Points and plain lines colored according to the test represent their point estimates and their 95% credibility interval. Plain and dashed horizontal lines represent the posterior estimates of the global model (considering all “departments”), which have been used as a prior for this analysis.


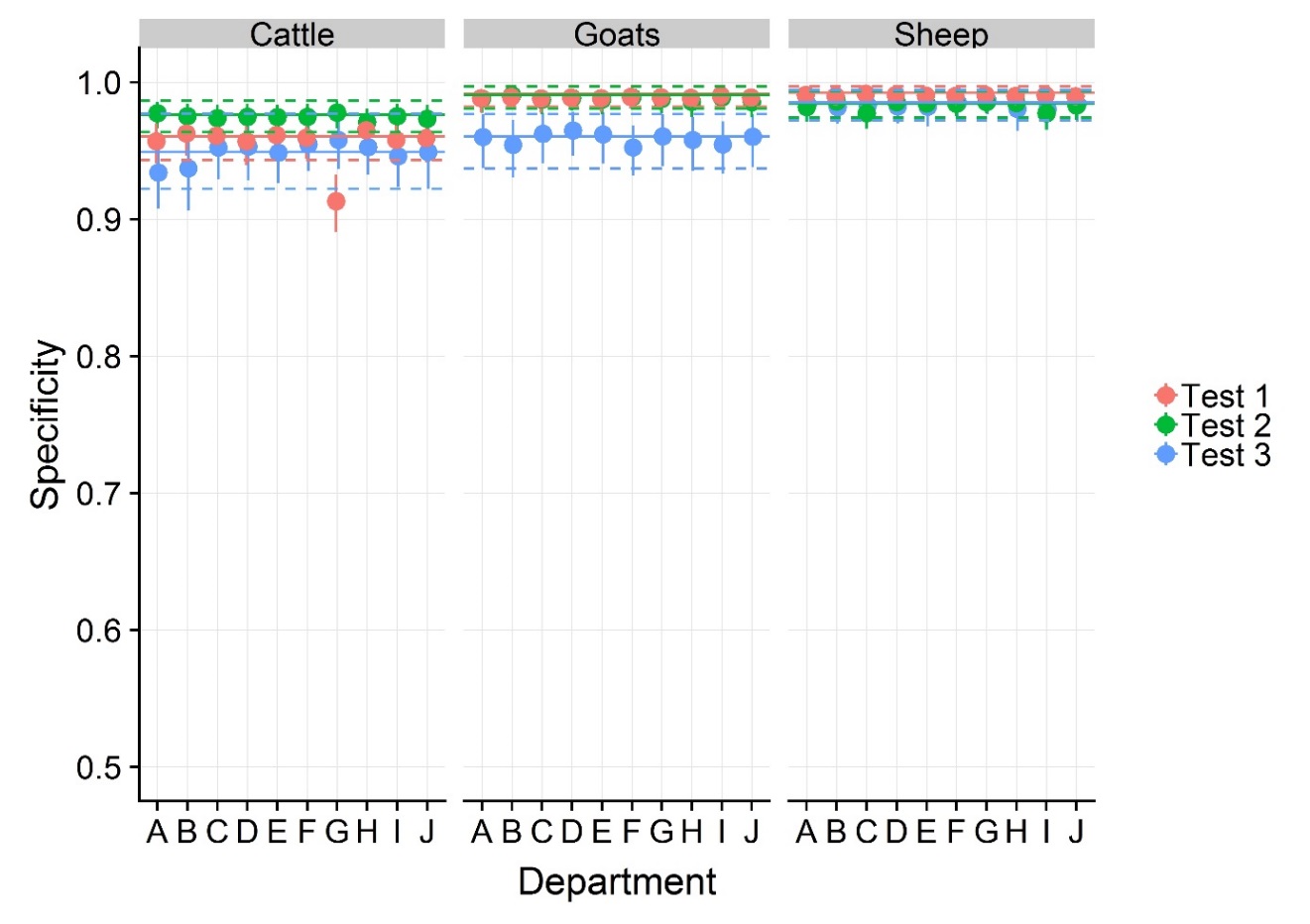


Figure A2: Posterior estimates of the specificities in each department and in each species. Points and plain lines colored according to the test represent their point estimates and their 95% credibility interval. Plain and dashed horizontal lines represent the posterior estimates of the global model in all departments, which has been used as a prior for this analysis.

**Appendix B. Complete specifications of the model**

In this section, we detail the specifications of the model, and provide a description of the probability vector of the multinomial distribution of $n_{ij}$the number of animals in each of the eight combinations of the free test results in the $j$th herd of the $i$th department in Table B1 and the inequality constraints included in the model.

**Table B1: Probability vector of the multinomial distribution of $n_{ij}$ the number of animals in each of the eight combinations of the free test results in the $j$th herd of the $i$th department (for example, the $T_{1}^{-}T_{2}^{+}T_{3}^{-}$ category corresponds to a serum with a positive test 2 and negative tests 1 and 3).**

| P | Category | Probability |
| --- | --- | --- |
| $p_{ij}\left[1\right]$ | $T_{1}^{-}T_{2}^{-}T_{3}^{-}$ | $\begin{matrix} WHP_{ij}\times\left(\left(1-Se_{1}\right)\times\left(1-Se_{2}\right)\times\left(1-Se_{3}\right)+\gamma_{Se000}\right)+ \\ \left(1-WHP_{ij}\right)\times\left(Sp_{1}\times Sp_{2}\times Sp_{3}+\gamma_{Sp000}\right) \end{matrix}$ |
| $p_{ij}\left[2\right]$ | $T_{1}^{-}T_{2}^{-}T_{3}^{+}$ | $\begin{matrix} WHP_{ij}\times\left(\left(1-Se_{1}\right)\times\left(1-Se_{2}\right)\times Se_{3}+\gamma_{Se001}\right)+ \\ \left(1-WHP_{ij}\right)\times\left(Sp_{1}\times Sp_{2}\times\left(1-Sp_{3}\right)+\gamma_{Sp001}\right) \end{matrix}$ |
| $p_{ij}\left[3\right]$ | $T_{1}^{-}T_{2}^{+}T_{3}^{-}$ | $\begin{matrix} WHP_{ij}\times\left(\left(1-Se_{1}\right)\times Se_{2}\times\left(1-Se_{3}\right)+\gamma_{Se010}\right)+ \\ \left(1-WHP_{ij}\right)\times\left(Sp_{1}\times\left(1-Sp_{2}\right)\times Sp_{3}+\gamma_{Sp010}\right) \end{matrix}$ |
| $p_{ij}\left[4\right]$ | $T_{1}^{-}T_{2}^{+}T_{3}^{+}$ | $\begin{matrix} WHP_{ij}\times\left(\left(1-Se_{1}\right)\times Se_{2}\times Se_{3}+\gamma_{Se011}\right)+ \\ \left(1-WHP_{ij}\right)\times\left(Sp_{1}\times\left(1-Sp_{2}\right)\times\left(1-Sp_{3}\right)+\gamma_{Sp011}\right) \end{matrix}$ |
| $p_{ij}\left[5\right]$ | $T_{1}^{+}T_{2}^{-}T_{3}^{-}$ | $\begin{matrix} WHP_{ij}\times\left(Se_{1}\times\left(1-Se_{2}\right)\times\left(1-Se_{3}\right)+\gamma_{Se100}\right)+ \\ \left(1-WHP_{ij}\right)\times\left(\left(1-Sp_{1}\right)\times Sp_{2}\times Sp_{3}+\gamma_{Sp100}\right) \end{matrix}$ |
| $p_{ij}\left[6\right]$ | $T_{1}^{+}T_{2}^{-}T_{3}^{+}$ | $\begin{matrix} WHP_{ij}\times\left(Se_{1}\times\left(1-Se_{2}\right)\times Se_{3}+\gamma_{Se101}\right)+ \\ \left(1-WHP_{ij}\right)\times\left(\left(1-Sp_{1}\right)\times Sp_{2}\times\left(1-Sp_{3}\right)+\gamma_{Sp101}\right) \end{matrix}$ |
| $p_{ij}\left[7\right]$ | $T_{1}^{+}T_{2}^{+}T_{3}^{-}$ | $\begin{matrix} WHP_{ij}\times\left(Se_{1}\times Se_{2}\times\left(1-Se_{3}\right)+\gamma_{Se110}\right)+ \\ \left(1-WHP_{ij}\right)\times\left(\left(1-Sp_{1}\right)\times\left(1-Sp_{2}\right)\times Sp_{3}+\gamma_{Sp110}\right) \end{matrix}$ |
| $p_{ij}\left[8\right]$ | $T_{1}^{+}T_{2}^{+}T_{3}^{+}$ | $\begin{matrix} WHP_{ij}\times\left(Se_{1}\times Se_{2}\times Se_{3}+\gamma_{Se111}\right)+ \\ \left(1-WHP_{ij}\right)\times\left(\left(1-Sp_{1}\right)\times\left(1-Sp_{2}\right)\times\left(1-Sp_{3}\right)+\gamma_{Sp111}\right) \end{matrix}$ |

According to Wang et al. [1], because of the total probability law, 8 of the 16 conditional dependence terms were expressed as a function of the eight others, according to the following equations:

$\begin{matrix} \gamma_{Se100}\text{= }\gamma_{Se011}+\gamma_{Se111}\text{-}\gamma_{Se000} \\ \gamma_{Se101}\text{= -}\left(\gamma_{Se001}+\gamma_{Se011}+\gamma_{Se111}\right) \\ \gamma_{Se110}\text{= }\gamma_{Se000}+\gamma_{Se001}\text{-}\gamma_{Se111} \\ \gamma_{Se010}\text{= -}\left(\gamma_{Se000}+\gamma_{Se001}+\gamma_{Se011}\right) \end{matrix}$ and $\begin{matrix} \gamma_{Sp100}\text{= }\gamma_{Sp011}+\gamma_{Sp111}\text{-}\gamma_{Sp000} \\ \gamma_{Sp101}\text{= -}\left(\gamma_{Sp001}+\gamma_{Sp011}+\gamma_{Sp111}\right) \\ \gamma_{Sp110}\text{= }\gamma_{Sp000}+\gamma_{Sp001}\text{-}\gamma_{Sp111} \\ \gamma_{Sp010}\text{= -}\left(\gamma_{Sp000}+\gamma_{Sp001}+\gamma_{Sp011}\right) \end{matrix}$

As every marginal probability in Table 1 belongs to $\left[0;1\right]$, each of the 16 conditional terms were constrained by the following inequalities:

$$\begin{matrix} \begin{matrix} & \end{matrix}\begin{matrix} & & \end{matrix}-Se_{1}\times Se_{2}\times Se_{3}<\gamma_{Se111}<\min\left(Se_{1},\min\left(Se_{2},Se_{3}\right)\right)-Se_{1}\times Se_{2}\times Se_{3} \\ \begin{matrix} & \end{matrix}\begin{matrix} & \end{matrix}-\left(1-Se_{1}\right)\times Se_{2}\times Se_{3}<\gamma_{Se011}<\min\left(\left(1-Se_{1}\right),\min\left(Se_{2},Se_{3}\right)\right)-\left(1-Se_{1}\right)\times Se_{2}\times Se_{3} \\ \begin{matrix} & \end{matrix}-\left(1-Se_{1}\right)\times\left(1-Se_{2}\right)\times Se_{3}<\gamma_{Se001}<\min\left(\left(1-Se_{1}\right),\min\left(\left(1-Se_{2}\right),Se_{3}\right)\right)-\left(1-Se_{1}\right)\times\left(1-Se_{2}\right)\times Se_{3} \\ -\left(1-Se_{1}\right)\times\left(1-Se_{2}\right)\times\left(1-Se_{3}\right)<\gamma_{Se000}<\min\left(\left(1-Se_{1}\right),\min\left(\left(1-Se_{2}\right),\left(1-Se_{3}\right)\right)\right)-\left(1-Se_{1}\right)\times\left(1-Se_{2}\right)\times\left(1-Se_{3}\right) \\ \begin{matrix} & \end{matrix}-Se_{1}\times\left(1-Se_{2}\right)\times\left(1-Se_{3}\right)<\gamma_{Se100}<\min\left(Se_{1},\min\left(\left(1-Se_{2}\right),\left(1-Se_{3}\right)\right)\right)-Se_{1}\times\left(1-Se_{2}\right)\times\left(1-Se_{3}\right) \\ \begin{matrix} & \end{matrix}\begin{matrix} & \end{matrix}-Se_{1}\times\left(1-Se_{2}\right)\times Se_{3}<\gamma_{Se101}<\min\left(Se_{1},\min\left(\left(1-Se_{2}\right),Se_{3}\right)\right)-Se_{1}\times\left(1-Se_{2}\right)\times Se_{3} \\ \begin{matrix} & \end{matrix}\begin{matrix} & \end{matrix}-Se_{1}\times Se_{2}\times\left(1-Se_{3}\right)<\gamma_{Se110}<\min\left(Se_{1},\min\left(Se_{2},\left(1-Se_{3}\right)\right)\right)-Se_{1}\times Se_{2}\times\left(1-Se_{3}\right) \\ \begin{matrix} & \end{matrix}-\left(1-Se_{1}\right)\times Se_{2}\times\left(1-Se_{3}\right)<\gamma_{Se010}<\min\left(\left(1-Se_{1}\right),\min\left(Se_{2},\left(1-Se_{3}\right)\right)\right)-\left(1-Se_{1}\right)\times Se_{2}\times\left(1-Se_{3}\right) \end{matrix}$$

$$\begin{matrix} \begin{matrix} & \end{matrix}\begin{matrix} & & \end{matrix}-Sp_{1}\times Sp_{2}\times Sp_{3}<\gamma_{Sp000}<\min\left(Sp_{1},\min\left(Sp_{2},Sp_{3}\right)\right)-Sp_{1}\times Sp_{2}\times Sp_{3} \\ \begin{matrix} & \end{matrix}\begin{matrix} & \end{matrix}-\left(1-Sp_{1}\right)\times Sp_{2}\times Sp_{3}<\gamma_{Sp100}<\min\left(\left(1-Sp_{1}\right),\min\left(Sp_{2},Sp_{3}\right)\right)-\left(1-Sp_{1}\right)\times Sp_{2}\times Sp_{3} \\ \begin{matrix} & \end{matrix}-\left(1-Sp_{1}\right)\times\left(1-Sp_{2}\right)\times Sp_{3}<\gamma_{Sp110}<\min\left(\left(1-Sp_{1}\right),\min\left(\left(1-Sp_{2}\right),Sp_{3}\right)\right)-\left(1-Sp_{1}\right)\times\left(1-Sp_{2}\right)\times Sp_{3} \\ -\left(1-Sp_{1}\right)\times\left(1-Sp_{2}\right)\times\left(1-Sp_{3}\right)<\gamma_{Sp111}<\min\left(\left(1-Sp_{1}\right),\min\left(\left(1-Sp_{2}\right),\left(1-Sp_{3}\right)\right)\right)-\left(1-Sp_{1}\right)\times\left(1-Sp_{2}\right)\times\left(1-Sp_{3}\right) \\ \begin{matrix} & \end{matrix}-Sp_{1}\times\left(1-Sp_{2}\right)\times\left(1-Sp_{3}\right)<\gamma_{Sp011}<\min\left(Sp_{1},\min\left(\left(1-Sp_{2}\right),\left(1-Sp_{3}\right)\right)\right)-Sp_{1}\times\left(1-Sp_{2}\right)\times\left(1-Sp_{3}\right) \\ \begin{matrix} & \end{matrix}\begin{matrix} & \end{matrix}-Sp_{1}\times\left(1-Sp_{2}\right)\times Sp_{3}<\gamma_{Sp010}<\min\left(Sp_{1},\min\left(\left(1-Sp_{2}\right),Sp_{3}\right)\right)-Sp_{1}\times\left(1-Sp_{2}\right)\times Sp_{3} \\ \begin{matrix} & \end{matrix}\begin{matrix} & \end{matrix}-Sp_{1}\times Sp_{2}\times\left(1-Sp_{3}\right)<\gamma_{Sp001}<\min\left(Sp_{1},\min\left(Sp_{2},\left(1-Sp_{3}\right)\right)\right)-Sp_{1}\times Sp_{2}\times\left(1-Sp_{3}\right) \\ \begin{matrix} & \end{matrix}-\left(1-Sp_{1}\right)\times Sp_{2}\times\left(1-Sp_{3}\right)<\gamma_{Sp101}<\min\left(\left(1-Sp_{1}\right),\min\left(Sp_{2},\left(1-Sp_{3}\right)\right)\right)-\left(1-Sp_{1}\right)\times Sp_{2}\times\left(1-Sp_{3}\right) \end{matrix}$$

To avoid the unidentifiability problem related to the mirror image with the same likelihood (when the model switches the labels of truly positive and truly negative results), we also constrained the sensitivities to be greater than the complement of the respective specificities. Then, we specified in the model that:

$$\begin{matrix} Se_{1}>1-Sp_{1} \\ Se_{2}>1-Sp_{2} \\ Se_{3}>1-Sp_{3} \end{matrix}$$

A runnable version of the model with the data set is also provided in the .zip file of the supplementary data.

**Appendix C. Simulation study**

The objectives of the simulation study were to assess (1) the potential bias and coverage probability of the latent class model when used to assess the sensitivities and specificities of the three diagnostic tests in a context similar to the one observed on the study dataset, and (2) the impact of using two different prior distributions for conditional dependence terms. We performed the simulations independently for goats, sheep and cattle.

Five simulation scenarios were considered. We generated 100 datasets per scenario. In these datasets, the numbers of herds per department, and of animals per herd, were set as equal to the sample size observed in the corresponding species (see Table 1 of the main manuscript for details). We analyzed all simulated datasets with two LCMs. The first LCM (hereafter named “Cauchy”) was identical to the LCM performed on the study dataset; it used a Cauchy prior distribution of location 0 and scale 0.039 for conditional dependence terms. The second LCM (hereafter named “Unif”) was similar to the LCM performed on the study dataset, but a uniform prior distribution between -0.5 and 0.5 was used for conditional dependence terms.

**Description of the five scenarios used to create simulation datasets**

Because the main parameters of interest were sensitivity, specificity and conditional dependence terms, the only parameters we changed between the five scenarios, for each species, were sensitivity, specificity or conditional dependence terms. Serorevalence terms (between-herd seroprevalence in every department and the hyperparameters ($\gamma_{P}$and $\mu_{P}$) for within-herd seroprevalence) were set at their respective estimated values in each species for the five scenarios (see Table E1 of Appendix E for details). The five scenarios were set to represent five plausible situations related to the estimations of the main manuscript (Table C1):

- Scenario 1, named “original scenario” corresponds to the original values assessed in the current study (Table E1)
- Scenario 2, named “High Se, High Sp and conditional independence scenario”, corresponds to a situation where tests are conditionally independent and sensitivity and specificity are consequently higher than in the original scenario
- Scenario 3, named “High Se, original Sp and low conditional dependence”, corresponds to a situation where tests are more sensitive and specific and less conditionally dependent than in the original scenario
- Scenario 4, named “Low Se, original Sp and conditional dependence”, corresponds to a situation where tests are less sensitive but equally specific to those in the original scenario and still conditionally dependent
- Scenario 5, named “Low Sp for test 3 only”, corresponds to the original situation, except that the specificity of one test (test 3) is set to a lower value.

As the sensitivity and specificity of the three tests varied broadly between tests and species, and with the aim of standardizing the deviation from the original scenario, we used a logit link to fix the values of the low and high parameters as follows:

$$\begin{matrix} Se_{Low}=\mathrm{logit}^{-1}\left(\mathrm{logit}\left(Se_{Orignial}\right)-1\right) \\ Se_{High}=\mathrm{logit}^{-1}\left(\mathrm{logit}\left(Se_{Orignial}\right)+1\right) \\ Sp_{High}=\mathrm{logit}^{-1}\left(\mathrm{logit}\left(Sp_{Orignial}\right)+1\right) \end{matrix}$$

Where, $Se_{Original}$ and $Sp_{Original}$ are respectively the sensitivity and specificity values assessed in the main manuscript, $\mathrm{logit}$ is the logit link function defined as $\mathrm{logit}\left(x\right)=\ln\left(\frac{x}{1-x}\right)$

$\mathrm{logit}^{-1}$is the inverse logit function defined as $\mathrm{logit}^{-1}\left(x\right)=\frac{\exp\left(x\right)}{1+\exp\left(x\right)}$.

In scenario 5, deviations from the original value of the specificity of test 3 ($Sp3$) was chosen to be slightly higher than for other tests (as in scenarios 1 to 4) in order to assess the stability of the estimations if one of the tests had a specificity much lower than the others.

$$Sp_{3Low}=\mathrm{logit}^{-1}\left(\mathrm{logit}\left(Sp_{3Orignal}\right)-1.5\right)$$

For scenarios in which tests are conditionally dependent (scenarios 1, 3, 4 and 5), all conditional dependence terms have to be fixed while respecting their inequality constraints (see Appendix B for details). To do so, we ensure that, for each conditional term, the ratio between its value and the maximum value allowed by its respective constraints stays as close as possible to the corresponding ratio in the original scenario.

As an example, we detail the calculation of $\gamma_{Se111SC3}$, i.e. the value of $\gamma_{Se111}$ in scenario 3. The inequality constraint of $\gamma_{Se111}$is expressed as follows:

$$-Se_{1}\times Se_{2}\times Se_{3}<\gamma_{Se111}<\min\left(Se_{1},\min\left(Se_{2},Se_{3}\right)\right)-Se_{1}\times Se_{2}\times Se_{3}$$

Then the maximum possible value of $\gamma_{Se111}$ in the original scenario is equal to:

$$\max\left(\gamma_{Se111}\right)_{SC1}=\min\left(Se_{1SC1},\min\left(Se_{2SC1},Se_{3SC1}\right)\right)-Se_{1SC1}\times Se_{2SC1}\times Se_{3SC1}$$

Where$Se_{1SC1}$,$Se_{2SC1}$and $Se_{3SC1}$are the sensitivity of tests 1, 2 and 3, respectively in the original scenario.

The ratio between $\gamma_{Se111SC1}$ and its maximum values is noted$Ratio_{MAX}\left(\gamma_{Se111}\right)$and is equal to:

$$Ratio_{MAX}\left(\gamma_{Se111}\right)=\frac{\gamma_{Se111SC1}}{\min\left(Se_{1SC1},\min\left(Se_{2SC1},Se_{3SC1}\right)\right)-Se_{1SC1}\times Se_{2SC1}\times Se_{3SC1}}$$

Finally, we fixed $\gamma_{Se111SC3}$ the value of $\gamma_{Se111}$ in scenario 3 as follows:

$$\gamma_{Se111SC3}=Ratio_{MAX}\left(\gamma_{Se111}\right)\times\left(\min\left(Se_{1SC3},\min\left(Se_{2SC3},Se_{3SC3}\right)\right)-Se_{1SC3}\times Se_{2SC3}\times Se_{3SC3}\right)$$

Where$Se_{1SC3}$,$Se_{2SC3}$ and$Se_{3SC3}$are the sensitivity of tests 1, 2 and 3, respectively in scenario 3.

We applied the same procedure for other conditional dependence terms and scenarios. After following this procedure, when certain inequality constraints were still not satisfied, we arbitrarily chose values for the corresponding parameters near to the one calculated as described above.

The set of values used to simulate the datasets of scenarios 1 to 5 is provided in Table C1.

**Table C1: Values of sensitivity, specificity and conditional dependence terms used to generate 100 datasets for each scenario.**

|  | Goats | | | | | Sheep | | | | | Cattle | | | | |
| --- | --- | --- | --- | --- | --- | --- | --- | --- | --- | --- | --- | --- | --- | --- | --- |
| Parameters | SC1 | SC2 | SC3 | SC4 | SC5 | SC1 | SC2 | SC3 | SC4 | SC5 | SC1 | SC2 | SC3 | SC4 | SC5 |
| $Se_{1}$ | 0.592 | 0.797 | 0.797 | 0.348 | 0.59 | 0.395 | 0.639 | 0.639 | 0.193 | 0.395 | 0.723 | 0.876 | 0.876 | 0.489 | 0.723 |
| $Se_{2}$ | 0.751 | 0.891 | 0.891 | 0.525 | 0.751 | 0.541 | 0.762 | 0.762 | 0.302 | 0.541 | 0.621 | 0.816 | 0.816 | 0.376 | 0.621 |
| $Se_{3}$ | 0.905 | 0.962 | 0.962 | 0.778 | 0.905 | 0.871 | 0.948 | 0.948 | 0.712 | 0.871 | 0.891 | 0.956 | 0.956 | 0.75 | 0.891 |
| $Sp_{1}$ | 0.991 | 0.996 | 0.991 | 0.991 | 0.991 | 0.992 | 0.997 | 0.992 | 0.992 | 0.992 | 0.960 | 0.984 | 0.960 | 0.960 | 0.960 |
| $Sp_{2}$ | 0.991 | 0.996 | 0.991 | 0.991 | 0.991 | 0.984 | 0.994 | 0.984 | 0.984 | 0.984 | 0.976 | 0.991 | 0.976 | 0.976 | 0.976 |
| $Sp_{3}$ | 0.96 | 0.984 | 0.96 | 0.96 | 0.842 | 0.985 | 0.994 | 0.985 | 0.985 | 0.936 | 0.949 | 0.980 | 0.949 | 0.949 | 0.805 |
| $\gamma_{Se000}$ | 0.013 | 0 | 0.006 | 0.013 | 0.013 | 0.01 | 0 | 0.005 | 0.020 | 0.01 | 0.015 | 0 | 0.007 | 0.015 | 0.015 |
| $\gamma_{Se001}$ | 0.098 | 0 | 0.054 | 0.098 | 0.098 | 0.137 | 0 | 0.098 | 0.085 | 0.137 | 0.136 | 0 | 0.075 | 0.136 | 0.136 |
| $\gamma_{Se011}$ | -0.106 | 0 | -0.053 | -0.106 | -0.106 | -0.154 | 0 | -0.09 | -0.12 | -0.154 | -0.142 | 0 | -0.081 | -0.142 | -0.142 |
| $\gamma_{Se111}$ | 0.124 | 0 | 0.074 | 0.124 | 0.124 | 0.166 | 0 | 0.125 | 0.12 | 0.166 | 0.160 | 0 | 0.096 | 0.160 | 0.160 |
| $\gamma_{Sp000}$ | 0.002 | 0 | 0.002 | 0.002 | 0 | 0.005 | 0 | 0.005 | 0.005 | 0 | 0.046 | 0 | 0.046 | 0.046 | 0 |
| $\gamma_{Sp001}$ | 0.001 | 0 | 0.001 | 0.001 | 0 | -0.003 | 0 | -0.003 | -0.003 | 0 | -0.027 | 0 | -0.027 | -0.027 | 0 |
| $\gamma_{Sp011}$ | 0.011 | 0 | 0.011 | 0.011 | 0 | 0.001 | 0 | 0.001 | 0.001 | 0 | 0.001 | 0 | 0.001 | 0.001 | 0 |
| $\gamma_{Sp111}$ | -0.007 | 0 | -0.007 | -0.007 | 0 | 0.001 | 0 | 0.001 | 0.001 | 0 | 0.018 | 0 | 0.018 | 0.018 | 0 |

**Assessment of estimator performances**

Accuracy of the sensitivity and specificity estimators was initially assessed graphically by plotting the “boxplot” of the 100 point estimates of the sensitivity and specificity values obtained with the five simulation scenarios in goats, sheep and cattle (Figures C1, C2 and C3, respectively).

We calculated the mean bias (Tables C2 and C4 for the “Cauchy” and “Unif” models, respectively) and the coverage probability (Table C3 and C5 for the “Cauchy” and “Unif” models, respectively) corresponding to all parameters of each scenario and species. Finally, we compared the quadratic error mean of all sensitivity and specificity estimators between both models (Tables C6, C7 and C8 for cattle, sheep and goats, respectively).

For each parameter, the mean bias was defined as the mean difference between its point estimates and the value used to simulate the datasets:

$$\overline{Bias}\left(X\right)=\frac{\sum_{k=1}^{Nsim} \left(\tilde{X}_{k}-X_{Sim}\right)}{Nsim}$$

Where $\overline{Bias}\left(X\right)$is the mean bias of the parameter named $X$,$\tilde{X}_{k}$is the point estimate of $X$obtained from the $k^{th}$ simulated dataset, and $X_{Sim}$ is the “true” value of $X$ used to simulate the dataset in the corresponding scenario.

For each parameter, the coverage probability was defined as the number out of 100 of 95% credible intervals which includes the value used to simulate the datasets.

For each parameter, the quadratic error mean was defined as the mean of the square of the difference between its point estimates and the value used to simulate the datasets as follows:

$$\overline{QEM}\left(X\right)=\frac{\sum_{k=1}^{Nsim} \left(\tilde{X}_{k}-X_{Sim}\right)^{2}}{Nsim}$$

Where $\overline{QEM}\left(X\right)$ is the quadratic error mean of the parameter named $X$


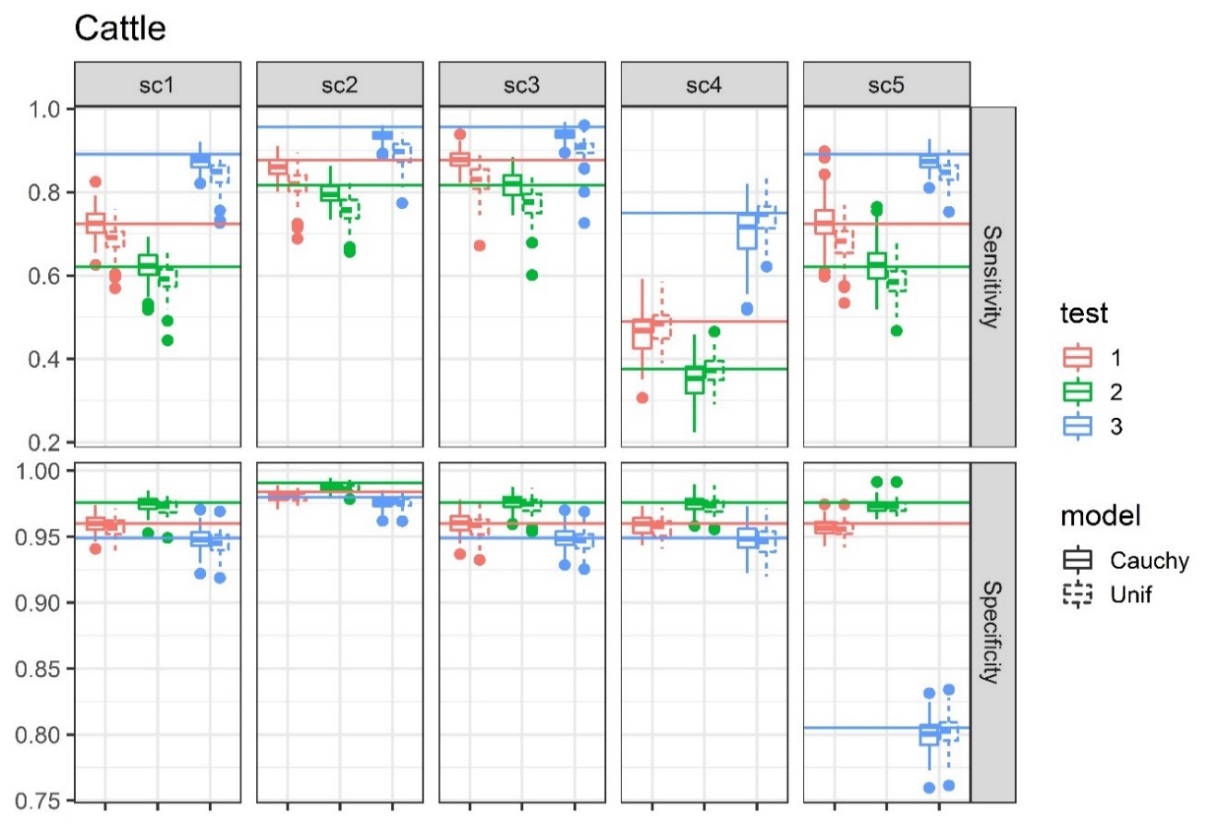


Figure C1: Boxplot of the 100 point estimates of the sensitivity and specificity values obtained with the five simulation scenarios for cattle and both prior distributions for conditional dependence terms: Cauchy (0,0.039) in plain line and uniform (-0.5,0.5) in dotted line. Plain horizontal lines represent the expected value of each parameter used to generate the dataset in the corresponding scenario.


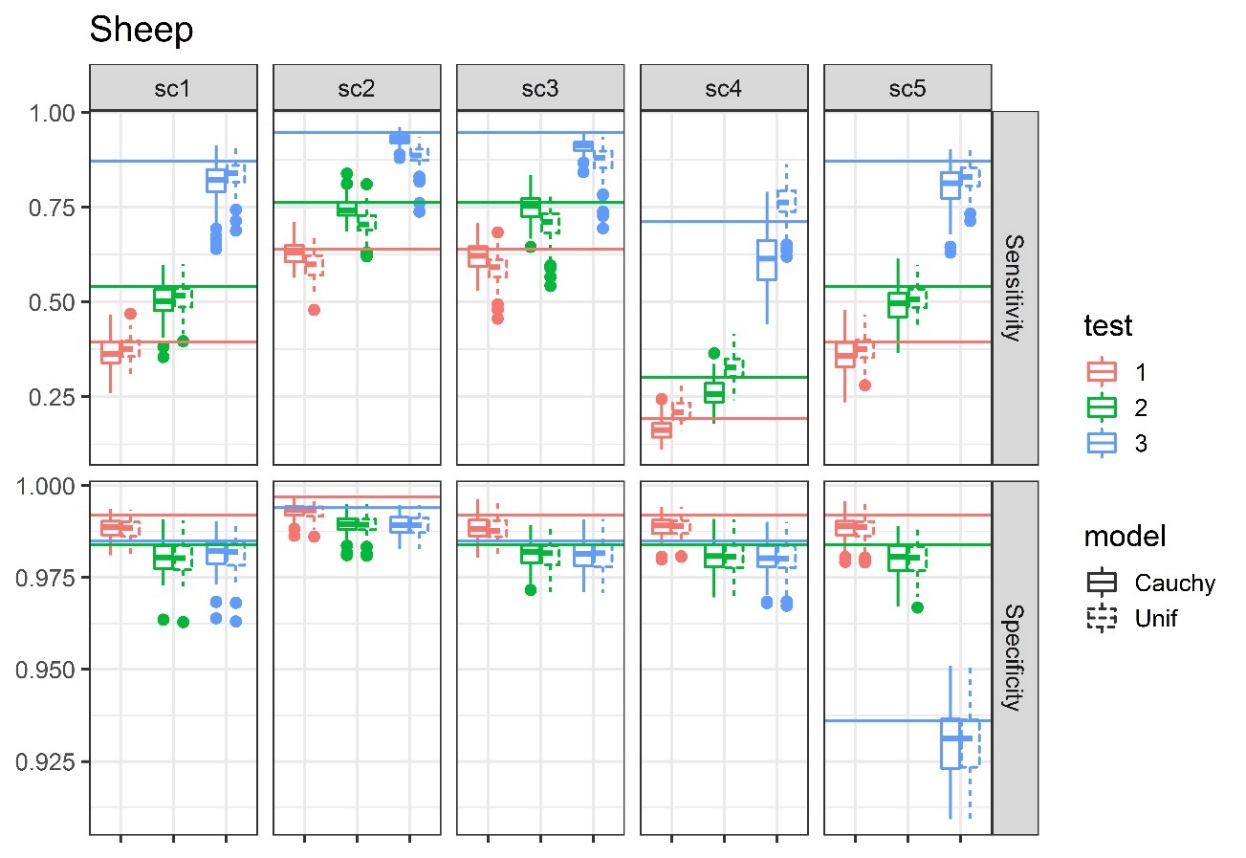


Figure C2: Boxplot of the 100 point estimates of the sensitivity and specificity values obtained with the five simulation scenarios for sheep and both prior distributions for conditional dependence terms: Cauchy (0,0.039) in plain line and uniform (-0.5,0.5) in dotted line. Plain horizontal lines represent the expected value of each parameter used to generate the dataset in the corresponding scenario.


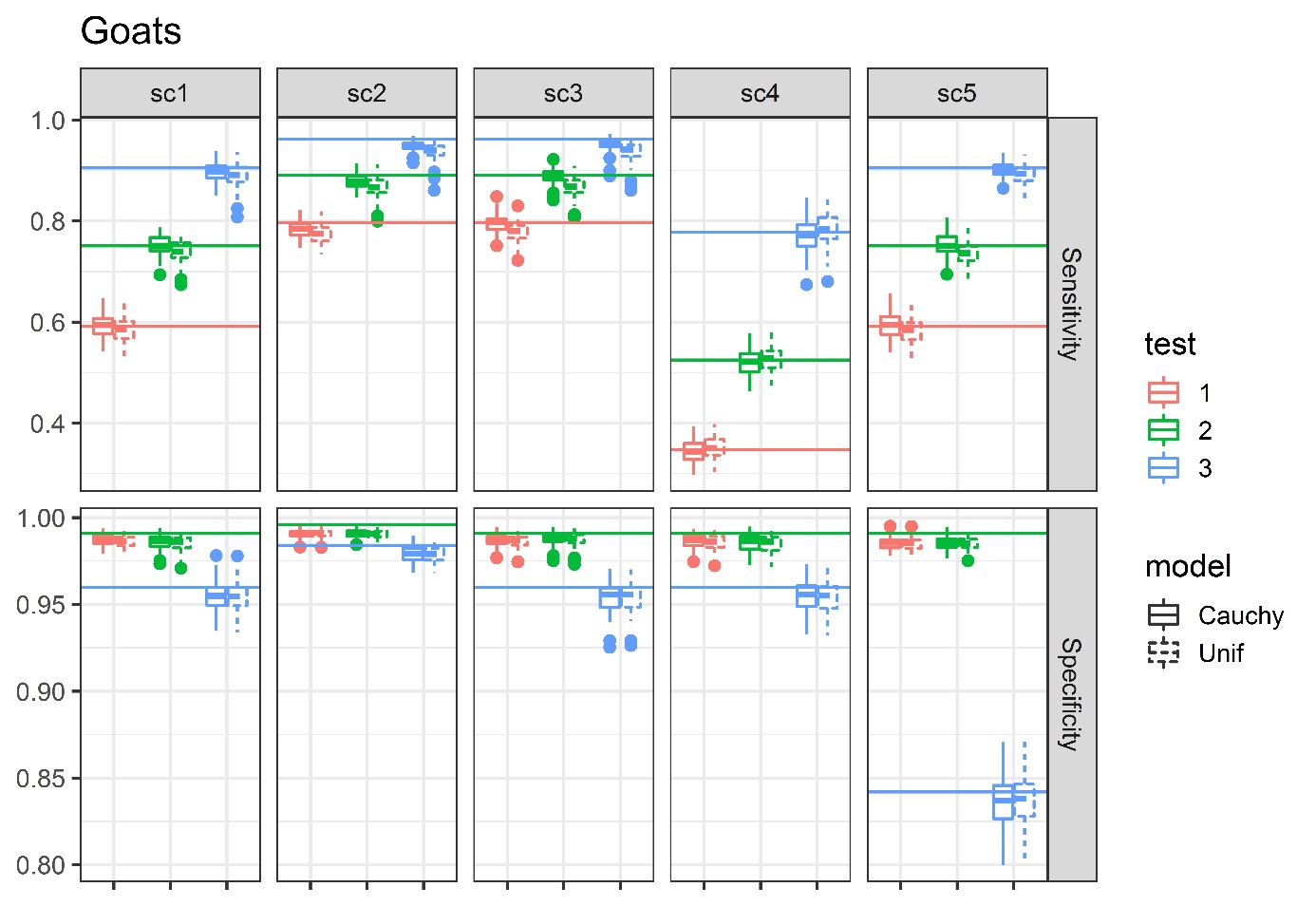


Figure C3: Boxplot of the 100 point estimates of the sensitivity and specificity values obtained with the five simulation scenarios for cattle and both prior distributions for conditional dependence terms: Cauchy (0,0.039) in plain line and uniform (-0.5,0.5) in dotted line. Plain horizontal lines represent the expected value of each parameter used to generate the dataset in the corresponding scenario.

**Table C2: Mean bias of sensitivity and specificity estimators obtained with the “Cauchy” model for the five scenarios in the three species**

|  | Goats | | | | | Sheep | | | | | Cattle | | | | |
| --- | --- | --- | --- | --- | --- | --- | --- | --- | --- | --- | --- | --- | --- | --- | --- |
| Parameters | SC1 | SC2 | SC3 | SC4 | SC5 | SC1 | SC2 | SC3 | SC4 | SC5 | SC1 | SC2 | SC3 | SC4 | SC5 |
| Se1 | 0.0010 | -0.0122 | -0.0015 | -0.0033 | 0.0023 | -0.0313 | -0.0113 | -0.0181 | -0.0273 | -0.0360 | 0.0010 | -0.0151 | 0.0012 | -0.0303 | 0.0077 |
| Se2 | 0.0014 | -0.0126 | -0.0026 | -0.0056 | 0.0026 | -0.0401 | -0.0172 | -0.0144 | -0.0418 | -0.0463 | -0.0019 | -0.0195 | 0.0016 | -0.0268 | 0.0064 |
| Se3 | -0.0081 | -0.0132 | -0.0102 | -0.0084 | -0.0043 | -0.0595 | -0.0192 | -0.0368 | -0.0984 | -0.0709 | -0.0179 | -0.0203 | -0.0174 | -0.0484 | -0.0178 |
| Sp1 | -0.0040 | -0.0050 | -0.0038 | -0.0045 | -0.0056 | -0.0036 | -0.0040 | -0.0036 | -0.0032 | -0.0036 | -0.0005 | -0.0039 | 0.0000 | -0.0014 | -0.0030 |
| Sp2 | -0.0047 | -0.0052 | -0.0029 | -0.0054 | -0.0057 | -0.0034 | -0.0046 | -0.0023 | -0.0034 | -0.0037 | -0.0010 | -0.0042 | 0.0000 | -0.0016 | -0.0029 |
| Sp3 | -0.0052 | -0.0053 | -0.0057 | -0.0046 | -0.0062 | -0.0037 | -0.0047 | -0.0039 | -0.0045 | -0.0058 | -0.0008 | -0.0042 | -0.0005 | -0.0011 | -0.0051 |
| Mean | -0.0033 | -0.0089 | -0.0044 | -0.0053 | -0.0028 | -0.0236 | -0.0102 | -0.0132 | -0.0298 | -0.0277 | -0.0035 | -0.0112 | -0.0025 | -0.0183 | -0.0024 |

**Table C3: Coverage probability of sensitivity and specificity estimators obtained with the “Cauchy” model for the five scenarios in the three species**

|  | Goats | | | | | Sheep | | | | | Cattle | | | | |
| --- | --- | --- | --- | --- | --- | --- | --- | --- | --- | --- | --- | --- | --- | --- | --- |
| Parameters | SC1 | SC2 | SC3 | SC4 | SC5 | SC1 | SC2 | SC3 | SC4 | SC5 | SC1 | SC2 | SC3 | SC4 | SC5 |
| Se1 | 0.98 | 0.93 | 0.98 | 0.98 | 0.97 | 0.95 | 0.92 | 0.94 | 0.96 | 0.93 | 0.98 | 0.93 | 0.99 | 0.95 | 0.98 |
| Se2 | 1 | 0.90 | 1 | 1 | 0.97 | 0.91 | 0.91 | 0.96 | 0.92 | 0.96 | 0.97 | 0.92 | 0.96 | 0.95 | 0.99 |
| Se3 | 0.99 | 0.87 | 0.94 | 1 | 1 | 0.97 | 0.92 | 0.83 | 0.96 | 0.96 | 0.98 | 0.88 | 0.92 | 1 | 0.99 |
| Sp1 | 0.92 | 0.70 | 0.89 | 0.83 | 0.75 | 0.86 | 0.59 | 0.84 | 0.88 | 0.83 | 0.97 | 0.88 | 0.94 | 0.92 | 0.96 |
| Sp2 | 0.85 | 0.62 | 0.95 | 0.76 | 0.79 | 0.92 | 0.72 | 0.93 | 0.93 | 0.92 | 0.98 | 0.81 | 0.96 | 0.91 | 0.91 |
| Sp3 | 0.89 | 0.81 | 0.93 | 0.94 | 0.91 | 0.92 | 0.68 | 0.93 | 0.94 | 0.90 | 0.94 | 0.85 | 0.95 | 0.93 | 0.95 |
| Mean | 0.938 | 0.805 | 0.948 | 0.918 | 0.898 | 0.921 | 0.790 | 0.905 | 0.931 | 0.916 | 0.970 | 0.878 | 0.953 | 0.943 | 0.963 |

**Table C4: Mean bias of sensitivity and specificity estimators obtained with the “Unif” model for the five scenarios in the three species**

|  | Goats | | | | | Sheep | | | | | Cattle | | | | |
| --- | --- | --- | --- | --- | --- | --- | --- | --- | --- | --- | --- | --- | --- | --- | --- |
| Parameters | SC1 | SC2 | SC3 | SC4 | SC5 | SC1 | SC2 | SC3 | SC4 | SC5 | SC1 | SC2 | SC3 | SC4 | SC5 |
| Se1 | -0.0072 | -0.0226 | -0.0189 | 0.0037 | -0.0086 | -0.0177 | -0.0437 | -0.0510 | 0.0189 | -0.0195 | -0.0378 | -0.0586 | -0.0485 | -0.0117 | -0.0452 |
| Se2 | -0.0112 | -0.0242 | -0.0235 | 0.0016 | -0.0133 | -0.0283 | -0.0547 | -0.0584 | 0.0229 | -0.0300 | -0.0313 | -0.0602 | -0.0440 | -0.0050 | -0.0359 |
| Se3 | -0.0168 | -0.0250 | -0.0253 | 0.0045 | -0.0133 | -0.0380 | -0.0634 | -0.0777 | 0.0488 | -0.0427 | -0.0492 | -0.0655 | -0.0534 | -0.0107 | -0.0458 |
| Sp1 | -0.0047 | -0.0052 | -0.0044 | -0.0052 | -0.0059 | -0.0039 | -0.0041 | -0.0039 | -0.0032 | -0.0038 | -0.0035 | -0.0040 | -0.0028 | -0.0038 | -0.0034 |
| Sp2 | -0.0053 | -0.0054 | -0.0034 | -0.0060 | -0.0059 | -0.0037 | -0.0047 | -0.0027 | -0.0035 | -0.0038 | -0.0034 | -0.0043 | -0.0024 | -0.0033 | -0.0031 |
| Sp3 | -0.0056 | -0.0055 | -0.0055 | -0.0054 | -0.0050 | -0.0040 | -0.0048 | -0.0039 | -0.0047 | -0.0058 | -0.0035 | -0.0042 | -0.0025 | -0.0034 | -0.0028 |
| Mean | -0.0085 | -0.0147 | -0.0135 | -0.0011 | -0.0087 | -0.0159 | -0.0292 | -0.0330 | 0.0132 | -0.0176 | -0.0214 | -0.0328 | -0.0256 | -0.0063 | -0.0227 |

**Table C5: Coverage probability of sensitivity and specificity estimators obtained with the “Unif” model for the five scenarios in the three species**

|  | Goats | | | | | Sheep | | | | | Cattle | | | | |
| --- | --- | --- | --- | --- | --- | --- | --- | --- | --- | --- | --- | --- | --- | --- | --- |
| Parameters | SC1 | SC2 | SC3 | SC4 | SC5 | SC1 | SC2 | SC3 | SC4 | SC5 | SC1 | SC2 | SC3 | SC4 | SC5 |
| Se1 | 0.98 | 0.86 | 0.94 | 0.99 | 0.96 | 0.97 | 0.89 | 0.87 | 0.98 | 0.93 | 0.97 | 0.78 | 0.83 | 0.99 | 0.94 |
| Se2 | 0.98 | 0.85 | 0.93 | 0.99 | 0.98 | 0.95 | 0.86 | 0.81 | 0.99 | 0.99 | 0.94 | 0.75 | 0.87 | 1.00 | 0.99 |
| Se3 | 0.98 | 0.72 | 0.85 | 1.00 | 0.99 | 0.98 | 0.74 | 0.71 | 1.00 | 0.97 | 0.95 | 0.57 | 0.81 | 1.00 | 0.97 |
| Sp1 | 0.87 | 0.68 | 0.88 | 0.81 | 0.75 | 0.84 | 0.58 | 0.81 | 0.89 | 0.82 | 0.94 | 0.88 | 0.90 | 0.88 | 0.95 |
| Sp2 | 0.84 | 0.61 | 0.94 | 0.74 | 0.77 | 0.91 | 0.74 | 0.93 | 0.92 | 0.90 | 0.96 | 0.81 | 0.93 | 0.88 | 0.88 |
| Sp3 | 0.89 | 0.81 | 0.94 | 0.94 | 0.94 | 0.92 | 0.68 | 0.93 | 0.94 | 0.90 | 0.94 | 0.86 | 0.94 | 0.90 | 0.96 |
| Mean | 0.923 | 0.755 | 0.913 | 0.912 | 0.898 | 0.928 | 0.748 | 0.843 | 0.953 | 0.918 | 0.950 | 0.775 | 0.880 | 0.942 | 0.948 |

**Table C6: Square roots of the quadratic error mean for sensitivity and specificity estimators obtained with both alternative prior distributions for conditional dependence terms for the five scenarios of the simulation study in cattle**

|  | Cattle | | | | | | | | | |
| --- | --- | --- | --- | --- | --- | --- | --- | --- | --- | --- |
| Scenario | SC1 | | SC2 | | SC3 | | SC4 | | SC5 | |
| Prior | Cauchy | Unif | Cauchy | Unif | Cauchy | Unif | Cauchy | Unif | Cauchy | Unif |
| Se1 | 0.032 | 0.051 | 0.026 | 0.067 | 0.025 | 0.058 | 0.062 | 0.042 | 0.053 | 0.063 |
| Se2 | 0.037 | 0.049 | 0.032 | 0.069 | 0.031 | 0.057 | 0.052 | 0.036 | 0.048 | 0.053 |
| Se3 | 0.027 | 0.059 | 0.025 | 0.072 | 0.022 | 0.06 | 0.079 | 0.042 | 0.027 | 0.053 |
| Sp1 | 0.007 | 0.008 | 0.005 | 0.005 | 0.008 | 0.009 | 0.007 | 0.008 | 0.006 | 0.007 |
| Sp2 | 0.005 | 0.007 | 0.005 | 0.005 | 0.006 | 0.007 | 0.006 | 0.007 | 0.006 | 0.006 |
| Sp3 | 0.008 | 0.009 | 0.006 | 0.006 | 0.008 | 0.008 | 0.01 | 0.011 | 0.014 | 0.013 |
| Mean | 0.019 | 0.03 | 0.016 | 0.037 | 0.017 | 0.033 | 0.036 | 0.024 | 0.026 | 0.032 |

**Table C7: Square roots of the quadratic error mean for sensitivity and specificity estimators obtained with both alternative prior distributions for conditional dependence terms for the five scenarios of the simulation study in sheep**

|  | Sheep | | | | | | | | | |
| --- | --- | --- | --- | --- | --- | --- | --- | --- | --- | --- |
| Scenario | SC1 | | SC2 | | SC3 | | SC4 | | SC5 | |
| Prior | Cauchy | Unif | Cauchy | Unif | Cauchy | Unif | Cauchy | Unif | Cauchy | Unif |
| Se1 | 0.049 | 0.035 | 0.032 | 0.056 | 0.041 | 0.065 | 0.04 | 0.034 | 0.059 | 0.042 |
| Se2 | 0.061 | 0.046 | 0.032 | 0.063 | 0.039 | 0.074 | 0.056 | 0.041 | 0.068 | 0.046 |
| Se3 | 0.08 | 0.054 | 0.024 | 0.071 | 0.041 | 0.089 | 0.124 | 0.068 | 0.094 | 0.058 |
| Sp1 | 0.004 | 0.005 | 0.004 | 0.005 | 0.005 | 0.005 | 0.004 | 0.004 | 0.005 | 0.005 |
| Sp2 | 0.006 | 0.006 | 0.005 | 0.005 | 0.004 | 0.005 | 0.005 | 0.005 | 0.006 | 0.006 |
| Sp3 | 0.006 | 0.006 | 0.005 | 0.006 | 0.006 | 0.006 | 0.006 | 0.006 | 0.011 | 0.011 |
| Mean | 0.034 | 0.025 | 0.017 | 0.034 | 0.023 | 0.041 | 0.039 | 0.026 | 0.04 | 0.028 |

**Table C8: Square roots of the quadratic error mean for sensitivity and specificity estimators obtained with both alternative prior distributions for conditional dependence terms for the five scenarios of the simulation study in goats**

|  | Goats | | | | | | | | | |
| --- | --- | --- | --- | --- | --- | --- | --- | --- | --- | --- |
| Scenario | SC1 | | SC2 | | SC3 | | SC4 | | SC5 | |
| Prior | Cauchy | Unif | Cauchy | Unif | Cauchy | Unif | Cauchy | Unif | Cauchy | Unif |
| Se1 | 0.023 | 0.024 | 0.021 | 0.03 | 0.017 | 0.027 | 0.022 | 0.021 | 0.024 | 0.026 |
| Se2 | 0.02 | 0.025 | 0.019 | 0.031 | 0.015 | 0.03 | 0.026 | 0.024 | 0.022 | 0.027 |
| Se3 | 0.019 | 0.028 | 0.016 | 0.03 | 0.016 | 0.032 | 0.034 | 0.033 | 0.015 | 0.023 |
| Sp1 | 0.005 | 0.006 | 0.005 | 0.006 | 0.005 | 0.006 | 0.006 | 0.007 | 0.007 | 0.007 |
| Sp2 | 0.006 | 0.007 | 0.006 | 0.006 | 0.005 | 0.005 | 0.007 | 0.008 | 0.007 | 0.007 |
| Sp3 | 0.01 | 0.01 | 0.007 | 0.007 | 0.01 | 0.01 | 0.01 | 0.01 | 0.015 | 0.015 |
| Mean | 0.014 | 0.017 | 0.012 | 0.018 | 0.011 | 0.018 | 0.018 | 0.017 | 0.015 | 0.017 |

**Appendix D. Herd sensitivity, herd specificity**

We considered a sample plan consisting of submitting $Nsample$ animals from one herd of size $Nherd$ to a test of sensitivity $Se$ and of specificity$Sp$. The herd was considered positive if at least 1 animal sampled is positive to the test (i.e. if$Npos_{sample}$, the number of sampled positive animals is greater than or equal to 1). The within-herd seroprevalence $WHP$was unknown but followed a beta distribution of location and precision parameters noted $\mu_{P}$ and $\gamma_{P}$, respectively:

$$WHP\sim beta\left(\mu_{P}\times\frac{\left(1-\gamma_{P}\right)}{\gamma_{P}},\left(1-\mu_{P}\right)\times\frac{\left(1-\gamma_{P}\right)}{\gamma_{P}}\right)$$

The objective of the study based on this sample plan was to identify all seropositive herds regardless of their $WHP$. We defined the corresponding herd sensitivity$HSe_{WHP>0}$ and specificity $HSp_{WHP=0}$ as follows:

$$\begin{matrix} HSe_{WHP>0}=1-P\left(Npos_{sample}=0|WHP>0\right) \\ HSp_{WHP=0}=P\left(Npos_{sample}=0|WHP=0\right) \end{matrix}$$

For a given $WHP$, we assumed that $Npos_{Herd}$ , the number of potential positive animals to the test, followed a binomial distribution of size $Nherd$ and of probability $WHP\times Se+\left(1-WHP\right)\times\left(1-Sp\right)$:

We assumed that$Npos_{Sampled}$, the number of positive individuals sampled, followed a hypergeometric distribution depending on the number of potential positive individuals in the herd, the herd size and the sample size ($Nsample$):

$$Npos_{Sampled}\sim hypergeometric\left(Nsample,Nherd,Npos_{Herd}\right)$$

Then, according to the total probability law, for every integer$n\in\left[0:Nherd\right]$:

$$\begin{matrix} P\left(Npos_{Sampled}=n|Nherd,Se,Sp,WHP\right)= \\ \sum_{x=0}^{Nherd} P\left(Npos_{Sampled}=n|Nherd,Nsample,Npos_{herd}=x\right)\times P\left(Npos_{herd}=x|Nherd,Se,Sp,WHP\right) \end{matrix}$$

where $P\left(Npos_{herd}=x|Nherd,Se,Sp,WHP\right)$was the probability density function of the binomial distribution (1.4)

$P\left(Npos_{Sampled}=n|Nherd,Nsample,Npos_{herd}=x\right)$ was the probability density function of the hypergeometric distribution (1.5)

According to the total probability law, we then weighted and integrated the expression (1.6) across all possible $WHP$as follows:

$$\begin{matrix} P\left(Npos_{Sampled}=n|Nherd,Se,Sp\right)= \\ \int_{0}^{1} P\left(WHP=P|\mu_{P},\gamma_{P}\right)\times P\left(Npos_{Sampled}=n|Nherd,Se,Sp,WHP=P\right)dP \end{matrix}$$

Where $P\left(WHP=P|\mu_{P},\gamma_{P}\right)$was the density probability function of the beta distribution (1.4)

Finally, considering a herd as positive if at least 1 animal was positive from the $Nsampled$animals sampled in a herd of size $Nherd$, we calculated the herd sensitivity corresponding to the non-free of *C. burnetii* Herd as:

$$HSe_{WHP>0}=1-\int_{0}^{1} P\left(WHP=P|\mu_{P},\gamma_{P}\right)\times P\left(Npos_{Sampled}=0|Nherd,Se,Sp,WHP=P\right)dP$$

The herd specificity of this situation was calculated from the expression (1.6) because $WHP$ was equal to 0:

$$HSp_{WHP=0}=\sum_{x=0}^{Nherd} P\left(Npos_{Sampled}=0|Nherd,Nsample,Npos_{herd}=x\right)\times Sp^{Nherd-x}\times\left(1-Sp\right)^{x}$$

**Appendix E. Estimations of the model**

Table E1: Estimations of the models for each parameter; point estimates and 95% credibility intervals (in square brackets) are the median and the 2.5 and 97.5 percentiles, respectively of the posterior distribution.

| **Parameter** | **Sheep** | **Goats** | **Cattle without department G** | **Cattle only in department G** |
| --- | --- | --- | --- | --- |
| $BHP_{A}$ | 0.929 [0.652;1] | 0.912 [0.655;0.999] | 0.078 [0;0.376] |  |
| $BHP_{B}$ | 0.941 [0.687;1] | 0.823 [0.54;0.979] | 0.232 [0.011;0.602] |  |
| $BHP_{C}$ | 0.06 [0;0.349] | 0.179 [0.024;0.462] | 0.969 [0.701;1] |  |
| $BHP_{D}$ | 0.148 [0.014;0.482] | 0.982 [0.82;1] | 0.648 [0.29;0.965] |  |
| $BHP_{E}$ | 0.204 [0.039;0.503] | 0.275 [0.073;0.57] | 0.681 [0.365;0.961] |  |
| $BHP_{F}$ | 0.021 [0;0.212] | 0.165 [0.017;0.496] | 0.492 [0.216;0.79] |  |
| $BHP_{G}$ | 0.022 [0;0.21] | 0.73 [0.439;0.933] |  | 0.071 [0;0.575] |
| $BHP_{H}$ | 0.965 [0.707;1] | 0.196 [0;0.96] | 0.023 [0;0.242] |  |
| $BHP_{I}$ | 0.47 [0.143;0.854] | 0.231 [0.05;0.525] | 0.064 [0;0.457] |  |
| $BHP_{J}$ | 0.931 [0.458;1] | 0.93 [0.684;1] | 0.306 [0.001;0.996] |  |
| $Se_{1}$ | 0.395 [0.312;0.471] | 0.592 [0.532;0.641] | 0.723 [0.618;0.811] | 0.568 [0.277;0.891]* |
| $Se_{2}$ | 0.541 [0.436;0.618] | 0.751 [0.682;0.798] | 0.621 [0.514;0.719] | 0.372 [0.063;0.845]* |
| $Se_{3}$ | 0.871 [0.715;0.937] | 0.905 [0.832;0.938] | 0.891 [0.788;0.941] | 0.475 [0.096;0.871]* |
| $Sp_{1}$ | 0.992 [0.985;0.997] | 0.991 [0.982;0.997] | **0.96 [0.943;0.977]** | **0.75 [0.676;0.86]** |
| $Sp_{2}$ | 0.984 [0.974;0.993] | 0.991 [0.98;0.997] | 0.976 [0.963;0.986] | 0.98 [0.952;0.994] |
| $Sp_{3}$ | 0.985 [0.973;0.994] | 0.96 [0.936;0.976] | 0.949 [0.922;0.977] | 0.97 [0.931;0.993] |
| $\gamma_{Se000}$ | 0.01 [-0.023;0.108] | 0.013 [-0.006;0.076] | 0.015 [-0.007;0.103] | -0.003 [-0.079;0.071] |
| $\gamma_{Se001}$ | 0.137 [0.044;0.175] | 0.098 [0.059;0.122] | 0.136 [0.077;0.176] | 0.002 [-0.061;0.076] |
| $\gamma_{Se011}$ | -0.154 [-0.188;-0.104] | -0.106 [-0.125;-0.085] | -0.142 [-0.169;-0.108] | -0.001 [-0.065;0.066] |
| $\gamma_{Se111}$ | 0.166 [0.138;0.196] | 0.124 [0.096;0.172] | 0.16 [0.118;0.221] | 0.005 [-0.069;0.082] |
| $\gamma_{Sp000}$ | 0.005 [0.001;0.012] | 0.011 [0.004;0.024] | 0.046 [0.025;0.068] | 0.015 [0.001;0.04] |
| $\gamma_{Sp001}$ | -0.003 [-0.008;-0.001] | -0.007 [-0.016;-0.002] | -0.027 [-0.039;-0.014] | -0.009 [-0.027;0.001] |
| $\gamma_{Sp011}$ | 0.001 [0;0.004] | 0.001 [0;0.006] | 0.001 [-0.001;0.005] | 0.003 [0;0.015] |
| $\gamma_{Sp111}$ | 0.001 [0;0.004] | 0.002 [0;0.009] | 0.018 [0.009;0.03] | 0.005 [0;0.02] |
| $\gamma_{P}$ | 0.204 [0.119;0.319] | 0.27 [0.169;0.432] | 0.224 [0.127;0.345] | 0.187 [0.002;0.966] |
| $\mu_{P}$ | 0.432 [0.347;0.549] | 0.679 [0.593;0.783] | 0.478 [0.374;0.609] | 0.535 [0.062;0.995] |

***** Because between-herd seroprevalence is close to 0 in department G, posterior distributions of the sensitivities are close to the uninformative prior distribution used in the model; hence, point estimates of sensitivity assessed only with data from department G only are not relevant.

**Appendix F. Sensitivity analysis**

Table F1: Estimations of the models with uniform ($Unif\left[-0.5;0.5\right]$ instead of $Cauchy\left(0,0.039\right)$ prior distribution for conditional dependence terms; point estimates and 95% credibility intervals (in square brackets) are the median and the 2.5 and 97.5 percentiles, respectively of the posterior distribution.

| **Parameter** | **Sheep** | **Goats** | **Cattle without department G** | **Cattle only in department G** |
| --- | --- | --- | --- | --- |
| $BHP_{A}$ | 0.93 [0.654;1] | 0.913 [0.659;0.999] | 0.076 [0;0.377] |  |
| $BHP_{B}$ | 0.941 [0.682;1] | 0.827 [0.544;0.979] | 0.225 [0.008;0.588] |  |
| $BHP_{C}$ | 0.06 [0;0.349] | 0.179 [0.024;0.456] | 0.967 [0.685;1] |  |
| $BHP_{D}$ | 0.147 [0.014;0.475] | 0.981 [0.815;1] | 0.61 [0.264;0.947] |  |
| $BHP_{E}$ | 0.203 [0.04;0.502] | 0.275 [0.071;0.572] | 0.664 [0.359;0.944] |  |
| $BHP_{F}$ | 0.021 [0;0.214] | 0.164 [0.017;0.49] | 0.474 [0.206;0.773] |  |
| $BHP_{G}$ | 0.021 [0;0.207] | 0.732 [0.443;0.934] |  | 0.068 [0;0.55] |
| $BHP_{H}$ | 0.964 [0.707;1] | 0.198 [0.001;0.966] | 0.022 [0;0.225] |  |
| $BHP_{I}$ | 0.475 [0.147;0.852] | 0.232 [0.051;0.524] | 0.05 [0;0.405] |  |
| $BHP_{J}$ | 0.933 [0.459;1] | 0.932 [0.681;1] | 0.292 [0.001;0.994] |  |
| $Se_{1}$ | 0.399 [0.322;0.472] | 0.583 [0.519;0.635] | 0.687 [0.567;0.783] | 0.565 [0.282;0.886] * |
| $Se_{2}$ | 0.54 [0.446;0.616] | 0.737 [0.663;0.79] | 0.595 [0.481;0.696] | 0.374 [0.067;0.842] * |
| $Se_{3}$ | 0.875 [0.737;0.939] | 0.894 [0.813;0.936] | 0.866 [0.721;0.936] | 0.479 [0.099;0.867] * |
| $Sp_{1}$ | 0.992 [0.985;0.997] | 0.991 [0.981;0.997] | 0.956 [0.938;0.973] | 0.75 [0.677;0.852] |
| $Sp_{2}$ | 0.984 [0.974;0.993] | 0.99 [0.98;0.997] | 0.973 [0.958;0.984] | 0.98 [0.953;0.994] |
| $Sp_{3}$ | 0.985 [0.972;0.994] | 0.96 [0.938;0.977] | 0.945 [0.918;0.972] | 0.97 [0.931;0.993] |
| $\gamma_{Se000}$ | 0.009 [-0.024;0.098] | 0.024 [-0.005;0.092] | 0.041 [-0.006;0.155] | -0.003 [-0.078;0.071] |
| $\gamma_{Se001}$ | 0.143 [0.059;0.18] | 0.096 [0.051;0.124] | 0.127 [0.04;0.177] | 0.002 [-0.062;0.076] |
| $\gamma_{Se011}$ | -0.16 [-0.192;-0.115] | -0.11 [-0.129;-0.091] | -0.15 [-0.174;-0.122] | -0.001 [-0.064;0.065] |
| $\gamma_{Se111}$ | 0.17 [0.143;0.2] | 0.135 [0.102;0.185] | 0.185 [0.134;0.248] | 0.005 [-0.07;0.082] |
| $\gamma_{Sp000}$ | 0.005 [0.001;0.012] | 0.011 [0.004;0.026] | 0.053 [0.031;0.077] | 0.015 [0.001;0.041] |
| $\gamma_{Sp001}$ | -0.003 [-0.009;-0.001] | -0.007 [-0.016;-0.002] | -0.03 [-0.043;-0.017] | -0.009 [-0.026;0.001] |
| $\gamma_{Sp011}$ | 0.001 [0;0.004] | 0.001 [0;0.007] | 0.001 [-0.001;0.005] | 0.003 [0;0.015] |
| $\gamma_{Sp111}$ | 0.001 [0;0.004] | 0.002 [0;0.009] | 0.021 [0.011;0.034] | 0.005 [0;0.02] |
| $\gamma_{P}$ | 0.205 [0.12;0.314] | 0.283 [0.173;0.469] | 0.237 [0.13;0.384] | 0.192 [0.002;0.971] |
| $\mu_{P}$ | 0.431 [0.347;0.54] | 0.688 [0.601;0.817] | 0.506 [0.39;0.672] | 0.555 [0.058;0.995] |

***** Because between-herd seroprevalence is close to 0 in department G, posterior distributions of the sensitivities are close to the uninformative prior distribution used in the model; Hence point estimates of sensitivity assessed only with data from department G only are not relevant.
