# Supplementary material for: Evaluation using latent class models of the diagnostic performances of three ELISA tests commercialized for the serological diagnosis of Coxiella burnetii infection in domestic ruminants
Source: Vet Res. 2021 Apr 14;52:56. doi: 10.1186/s13567-021-00926-w (PMC8048088; doi:10.1186/s13567-021-00926-w)

Output of the model

data <- read.table("data4paper.txt",header = TRUE)

# Description of the model in JAGS language

model.lclass <-
 "model
{

 # Number of animal from each population (herd) in each of the 8 categories of tests results
 for (i in 1:Npop)
 {

 n[i,] ~ dmulti(p[i,],N[i])

 p[i,1] <- Pinf[i] * ((1 - Se1) * (1 - Se2) * (1 - Se3) + covse_000) + (1 - Pinf[i]) * ((Sp1 * Sp2 * Sp3) + covsp_000)
 p[i,2] <- Pinf[i] * ((1 - Se1) * (1 - Se2) * Se3 + covse_001) + (1 - Pinf[i]) * (Sp1 * Sp2 * (1 - Sp3) + covsp_001)
 p[i,3] <- Pinf[i] * ((1 - Se1) * Se2 * (1 - Se3) + covse_010 ) + (1 - Pinf[i]) * (Sp1 * (1 - Sp2) * Sp3 + covsp_010)
 p[i,4] <- Pinf[i] * ((1 - Se1) * Se2 * Se3 + covse_011) + (1 - Pinf[i]) * (Sp1 * (1 - Sp2) * (1 - Sp3) + covsp_011)
 p[i,5] <- Pinf[i] * (Se1 * (1 - Se2) * (1 - Se3) + covse_100) + (1 - Pinf[i]) * ((1 - Sp1) * Sp2 * Sp3 + covsp_100)
 p[i,6] <- Pinf[i] * (Se1 * (1 - Se2) * Se3 + covse_101) + (1 - Pinf[i]) * ((1 - Sp1) * Sp2 * (1 - Sp3) + covsp_101)
 p[i,7] <- Pinf[i] * (Se1 * Se2 * (1 - Se3) + covse_110) + (1 - Pinf[i]) * ((1 - Sp1) * (1 - Sp2) * Sp3 + covsp_110)
 p[i,8] <- Pinf[i] * (Se1 * Se2 * Se3 + covse_111) + (1 - Pinf[i]) * ((1 - Sp1) * (1 - Sp2) * (1 - Sp3) + covsp_111)


 }

 # zero inflated beta-binomial distribution of the prevalence in each heard
 for(e in 1:Npop)
 {
 herdstatus[e] ~ dbern(Pherd[numdpt[e]])
 P[e] ~ dbeta(mup * (1 - gamma) / gamma, (1 - mup) * (1 - gamma) / gamma)T(0.00001,0.99999)
 Pinf[e] <- P[e] * herdstatus[e]

 # P[e] is at least 1 out the herdsize
 y_P[e] ~ dinterval(P[e], 1/Nherd[e])
 }

 # Calculation of the covariance terms
 covse_100 <- covse_011 + covse_111 - covse_000
 covse_101 <- -(covse_001 + covse_011 + covse_111)
 covse_110 <- covse_000 + covse_001 - covse_111
 covse_010 <- -(covse_000 + covse_001 + covse_011)

 covsp_100 <- covsp_011 + covsp_111 - covsp_000
 covsp_101 <- -(covsp_001 + covsp_011 + covsp_111)
 covsp_110 <- covsp_000 + covsp_001 - covsp_111
 covsp_010 <- -(covsp_000 + covsp_001 + covsp_011)

 ########
 # Prior#
 ########

 # Between herd prevalence of the jth departement
 for(j in 1:Ndpt)
 {
 Pherd[j] ~ dbeta(0.5,0.5)
 }


 mup ~ dbeta(0.5,0.5)T(0.00001,0.99999)
 gamma ~ dbeta(0.5,0.5)T(0.00001,0.99999)

 Se1 ~ dbeta(0.5,0.5)
 Se2 ~ dbeta(0.5,0.5)
 Se3 ~ dbeta(0.5,0.5)
 Sp1 ~ dbeta(0.5,0.5)
 Sp2 ~ dbeta(0.5,0.5)
 Sp3 ~ dbeta(0.5,0.5)

 covse_111 ~ dt(0, 1/0.039^2 , 1)
 covse_011 ~ dt(0, 1/0.039^2 , 1)
 covse_000 ~ dt(0, 1/0.039^2 , 1)
 covse_001 ~ dt(0, 1/0.039^2 , 1)

 covsp_111 ~ dt(0, 1/0.039^2 , 1)
 covsp_011 ~ dt(0, 1/0.039^2 , 1)
 covsp_000 ~ dt(0, 1/0.039^2 , 1)
 covsp_001 ~ dt(0, 1/0.039^2 , 1)

 ##########################
 # Inequality constraints #
 ##########################

 # Se > 1-Sp
 yse1 ~ dinterval(Se1 ,c(1-Sp1,1))
 yse2 ~ dinterval(Se2 ,c(1-Sp2,1))
 yse3 ~ dinterval(Se3 ,c(1-Sp3,1))

 # inequality constraints for covariance terms

 yse_000 ~ dinterval(covse_000 ,c(-(1-Se1)*(1-Se2)*(1-Se3),min(1-Se1, min(1-Se2, 1-Se3))-(1-Se1)*(1-Se2)*(1-Se3)))
 yse_001 ~ dinterval(covse_001 ,c(-(1-Se1)*(1-Se2)*Se3,min(1-Se1, min(1-Se2, Se3))-(1-Se1)*(1-Se2)*Se3))
 yse_010 ~ dinterval(covse_010 ,c(-(1-Se1)*Se2*(1-Se3),min(1-Se1, min(Se2, 1-Se3))-(1-Se1)*Se2*(1-Se3)))
 yse_011 ~ dinterval(covse_011 ,c(-(1-Se1)*Se2*Se3, min(1-Se1, min(Se2, Se3))-(1-Se1)*Se2*Se3))
 yse_100 ~ dinterval(covse_100 ,c(-Se1*(1-Se2)*(1-Se3),min(Se1, min((1-Se2), (1-Se3))) -Se1*(1-Se2)*(1-Se3)))
 yse_101 ~ dinterval(covse_101 ,c(-Se1*(1-Se2)*Se3, min(Se1, min((1-Se2), Se3))-Se1*(1-Se2)*Se3))
 yse_110 ~ dinterval(covse_110 ,c(-Se1*Se2*(1-Se3),min(Se1, min(Se2, 1-Se3))-Se1*Se2*(1-Se3)))
 yse_111 ~ dinterval(covse_111 ,c(-Se1*Se2*Se3,min(Se1, min(Se2, Se3)) -Se1*Se2*Se3))

 ysp_000 ~ dinterval(covsp_000 ,c(-Sp1*Sp2*Sp3,min(Sp1, min(Sp2, Sp3))-Sp1*Sp2*Sp3))
 ysp_001 ~ dinterval(covsp_001 ,c(-Sp1*Sp2*(1-Sp3), min(Sp1, min(Sp2, 1-Sp3))-Sp1*Sp2*(1-Sp3)))
 ysp_010 ~ dinterval(covsp_010 ,c(-Sp1*(1-Sp2)*Sp3,min(Sp1, min(1-Sp2, Sp3))-Sp1*(1-Sp2)*Sp3))
 ysp_011 ~ dinterval(covsp_011 ,c(-Sp1*(1-Sp2)*(1-Sp3),min(Sp1, min(1-Sp2, 1-Sp3))-Sp1*(1-Sp2)*(1-Sp3)))
 ysp_100 ~ dinterval(covsp_100 ,c(-(1-Sp1)*Sp2*Sp3,min((1-Sp1),min(Sp2, Sp3))-(1-Sp1)*Sp2*Sp3))
 ysp_110 ~ dinterval(covsp_110 ,c(-(1-Sp1)*(1-Sp2)*Sp3,min((1-Sp1), min((1-Sp2), Sp3))-(1-Sp1)*(1-Sp2)*(1-Sp3)))
 ysp_101 ~ dinterval(covsp_101 ,c(-(1-Sp1)*Sp2*(1-Sp3),min((1-Sp1), min(Sp2, 1-Sp3))-(1-Sp1)*Sp2*(1-Sp3)))
 ysp_111 ~ dinterval(covsp_111 ,c(-(1-Sp1)*(1-Sp2)*(1-Sp3),min(1-Sp1, min(1-Sp2, 1-Sp3))-(1-Sp1)*(1-Sp2)*(1-Sp3)))

}"

# SHEEP

data_O <- subset(data, data$species == "sheep")
data_O$Nherd[is.na(data_O$Nherd)] <- median(data_O$Nherd,na.rm = T)

#Creation of argument data for jags.model

N <- rowSums(data_O[,4:11])

data4jags.lclass_O <- list(Npop = nrow(data_O),
 N = N,
 Nherd = data_O$Nherd,
 n = as.matrix(data_O[,4:11]),
 Ndpt=10,
 numdpt = data_O$dpt,
 yse_000 = 1,
 yse_001 = 1,
 yse_010 = 1,
 yse_011 = 1,
 yse_100 = 1,
 yse_101 = 1,
 yse_110 = 1,
 yse_111 = 1,
 ysp_000 = 1,
 ysp_001 = 1,
 ysp_010 = 1,
 ysp_011 = 1,
 ysp_100 = 1,
 ysp_101 = 1,
 ysp_110 = 1,
 ysp_111 = 1,
 yse1 = 1,
 yse2 = 1,
 yse3 = 1,
 y_P = rep(1,nrow(data_O)))

##################
# Initialisation #
##################

# initialization ensures that the parameters respect the constraints of the model

inits <- list(list(),list(),list())

for (k in 1:3) {

 Sp1 <- rbeta(1,5,1)
 Sp2 <- rbeta(1,5,1)
 Sp3 <- rbeta(1,5,1)

 Se1 <- runif(1,1-Sp1,1)
 Se2 <- runif(1,1-Sp2,1)
 Se3 <- runif(1,1-Sp3,1)


 covse_111 <- 0
 covse_011 <- 0
 covse_001 <- 0
 covse_000 <- 0

 covsp_111 <- 0
 covsp_011 <- 0
 covsp_001 <- 0
 covsp_000 <- 0

 P <- runif(length(data_O$Nherd),1/data_O$Nherd,1)

 inits[[k]] <- list(Se1 = Se1, Se2 = Se2, Se3 = Se3,Sp1 = Sp1,
 Sp2 = Sp2, Sp3 = Sp3, covse_111 = covse_111,
 covse_011 = covse_011, covse_001 = covse_001,
 covse_000 = covse_000, covsp_111 = covsp_111,
 covsp_011 = covsp_011, covsp_001 = covsp_001,
 covsp_000 = covsp_000, P = P)
}


##################################
# Inference using MCMC algorithm #
# This step can take a few hours #
##################################
m.lclass_O<- jags.model(file = textConnection(model.lclass),
 data = data4jags.lclass_O, n.chains = 3, inits = inits)

## Compiling model graph
## Resolving undeclared variables
## Allocating nodes
## Graph information:
## Observed stochastic nodes: 217
## Unobserved stochastic nodes: 224
## Total graph size: 3602
##
## Initializing model

update(m.lclass_O, n.iter = 10000) # burnin

mcmc.lclass_O<- coda.samples(m.lclass_O, c("mup","gamma","Pherd","Se1","Se2","Se3",
 "Sp1","Sp2","Sp3","covse_000","covse_001",
 "covse_011", "covse_111","covsp_000","covsp_001",
 "covsp_011", "covsp_111"), n.iter = 100000, thin = 20)


##############################
# Estimation and diagnostics #
##############################

# Check of the convergence
gelman.diag(mcmc.lclass_O)

## Potential scale reduction factors:
##
## Point est. Upper C.I.
## Pherd[1] 1.00 1.00
## Pherd[2] 1.00 1.00
## Pherd[3] 1.00 1.00
## Pherd[4] 1.00 1.00
## Pherd[5] 1.00 1.00
## Pherd[6] 1.00 1.00
## Pherd[7] 1.00 1.00
## Pherd[8] 1.00 1.00
## Pherd[9] 1.00 1.00
## Pherd[10] 1.00 1.00
## Se1 1.00 1.00
## Se2 1.00 1.00
## Se3 1.00 1.01
## Sp1 1.00 1.00
## Sp2 1.00 1.00
## Sp3 1.00 1.00
## covse_000 1.01 1.01
## covse_001 1.00 1.01
## covse_011 1.00 1.01
## covse_111 1.00 1.00
## covsp_000 1.00 1.01
## covsp_001 1.00 1.01
## covsp_011 1.00 1.00
## covsp_111 1.00 1.01
## gamma 1.00 1.00
## mup 1.00 1.01
##
## Multivariate psrf
##
## 1

# Parameter estimations
summary( mcmc.lclass_O)

##
## Iterations = 11020:111000
## Thinning interval = 20
## Number of chains = 3
## Sample size per chain = 5000
##
## 1. Empirical mean and standard deviation for each variable,
## plus standard error of the mean:
##
## Mean SD Naive SE Time-series SE
## Pherd[1] 0.8996588 0.096626 7.889e-04 8.016e-04
## Pherd[2] 0.9146210 0.085794 7.005e-04 7.092e-04
## Pherd[3] 0.0922484 0.097467 7.958e-04 8.075e-04
## Pherd[4] 0.1742365 0.124483 1.016e-03 1.027e-03
## Pherd[5] 0.2213847 0.122622 1.001e-03 1.010e-03
## Pherd[6] 0.0455057 0.061181 4.995e-04 5.088e-04
## Pherd[7] 0.0432429 0.058043 4.739e-04 4.848e-04
## Pherd[8] 0.9314721 0.082911 6.770e-04 6.713e-04
## Pherd[9] 0.4775170 0.187105 1.528e-03 1.619e-03
## Pherd[10] 0.8742590 0.148892 1.216e-03 1.213e-03
## Se1 0.3940116 0.041108 3.356e-04 9.696e-04
## Se2 0.5371163 0.046584 3.804e-04 1.190e-03
## Se3 0.8596919 0.056544 4.617e-04 1.936e-03
## Sp1 0.9918860 0.002987 2.439e-05 3.649e-05
## Sp2 0.9840102 0.004917 4.015e-05 4.695e-05
## Sp3 0.9845999 0.005705 4.658e-05 6.010e-05
## covse_000 0.0179285 0.033129 2.705e-04 1.199e-03
## covse_001 0.1308886 0.033074 2.700e-04 1.166e-03
## covse_011 -0.1520266 0.021164 1.728e-04 6.158e-04
## covse_111 0.1663616 0.014461 1.181e-04 2.462e-04
## covsp_000 0.0054197 0.002778 2.268e-05 5.246e-05
## covsp_001 -0.0036458 0.001965 1.604e-05 3.447e-05
## covsp_011 0.0008503 0.001058 8.639e-06 1.131e-05
## covsp_111 0.0010751 0.001079 8.808e-06 1.814e-05
## gamma 0.2066418 0.050092 4.090e-04 8.373e-04
## mup 0.4371037 0.050483 4.122e-04 1.111e-03
##
## 2. Quantiles for each variable:
##
## 2.5% 25% 50% 75% 97.5%
## Pherd[1] 6.523e-01 0.8506827 0.9271470 0.975983 0.9996886
## Pherd[2] 6.916e-01 0.8717028 0.9403639 0.982700 0.9998305
## Pherd[3] 1.300e-04 0.0167491 0.0608017 0.136963 0.3496278
## Pherd[4] 1.431e-02 0.0783466 0.1473697 0.243121 0.4801091
## Pherd[5] 3.726e-02 0.1280723 0.2026269 0.295526 0.5064522
## Pherd[6] 3.936e-05 0.0050430 0.0216913 0.062460 0.2222728
## Pherd[7] 4.773e-05 0.0046122 0.0206293 0.059084 0.2115973
## Pherd[8] 7.012e-01 0.9005902 0.9634397 0.991740 0.9999169
## Pherd[9] 1.384e-01 0.3417923 0.4692166 0.609527 0.8533406
## Pherd[10] 4.611e-01 0.8127012 0.9331868 0.984572 0.9998526
## Se1 3.077e-01 0.3682866 0.3954794 0.422041 0.4706017
## Se2 4.313e-01 0.5097830 0.5406268 0.569001 0.6185342
## Se3 7.075e-01 0.8341144 0.8714311 0.898762 0.9363896
## Sp1 9.852e-01 0.9900691 0.9922102 0.994056 0.9967776
## Sp2 9.739e-01 0.9808077 0.9841189 0.987404 0.9931310
## Sp3 9.724e-01 0.9809656 0.9850514 0.988768 0.9941984
## covse_000 -2.208e-02 -0.0056058 0.0095681 0.032060 0.1096620
## covse_001 4.126e-02 0.1159465 0.1381319 0.153902 0.1751196
## covse_011 -1.876e-01 -0.1666995 -0.1540953 -0.139918 -0.1023251
## covse_111 1.381e-01 0.1566791 0.1660644 0.175957 0.1954493
## covsp_000 1.419e-03 0.0033977 0.0049443 0.006940 0.0122256
## covsp_001 -8.252e-03 -0.0047619 -0.0033184 -0.002195 -0.0007575
## covsp_011 -2.663e-04 0.0001045 0.0005281 0.001278 0.0036257
## covsp_111 2.559e-05 0.0003066 0.0007431 0.001474 0.0040367
## gamma 1.194e-01 0.1715442 0.2027867 0.237248 0.3147534
## mup 3.493e-01 0.4029875 0.4327849 0.466216 0.5508722

######################
# Prior vs posterior #
######################


data4jags.lclass_prior_O <- list(Npop = nrow(data_O),
 N = rep(0,nrow(data_O)),
 Nherd = data_O$Nherd,
 n = matrix(data=0, nrow= nrow(data_O), ncol=8),
 Ndpt=10,
 numdpt = data_O$dpt,
 yse_000 = 1,
 yse_001 = 1,
 yse_010 = 1,
 yse_011 = 1,
 yse_100 = 1,
 yse_101 = 1,
 yse_110 = 1,
 yse_111 = 1,
 ysp_000 = 1,
 ysp_001 = 1,
 ysp_010 = 1,
 ysp_011 = 1,
 ysp_100 = 1,
 ysp_101 = 1,
 ysp_110 = 1,
 ysp_111 = 1,
 yse1 = 1,
 yse2 = 1,
 yse3 = 1,
 y_P = rep(1,nrow(data_O)))

 m.lclass_prior_O<- jags.model(file = textConnection(model.lclass), data = data4jags.lclass_prior_O, n.chains = 3, inits = inits)

## Compiling model graph
## Resolving undeclared variables
## Allocating nodes
## Graph information:
## Observed stochastic nodes: 217
## Unobserved stochastic nodes: 224
## Total graph size: 3602
##
## Initializing model

update(m.lclass_prior_O, n.iter = 10000) # burnin


 mcmc.lclass_prior_O<- coda.samples(m.lclass_prior_O, c("mup","gamma","Pherd","Se1","Se2","Se3","Sp1","Sp2","Sp3","covse_000","covse_001", "covse_011", "covse_111","covsp_000","covsp_001", "covsp_011", "covsp_111"), n.iter = 100000, thin = 20)


MCMCtrace( mcmc.lclass_O,
 ISB = FALSE,
 priors = as.matrix( mcmc.lclass_prior_O),
 pdf = FALSE,
 Rhat = FALSE,
 n.eff = FALSE)


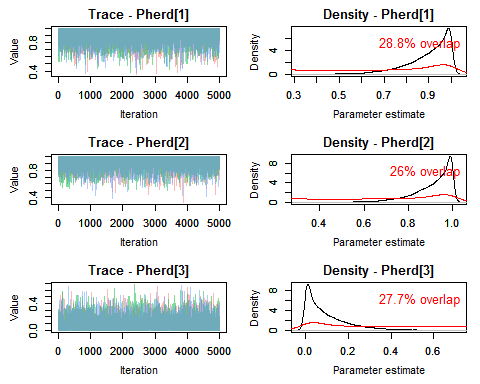

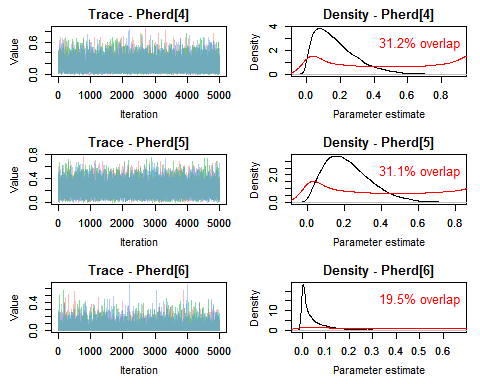

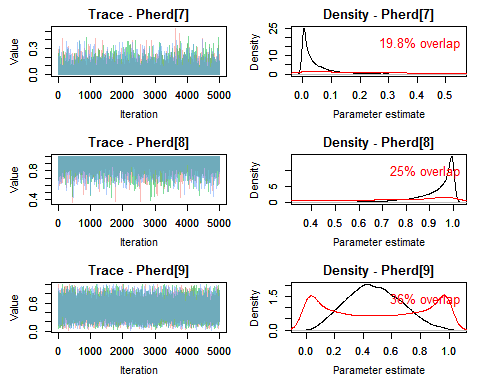

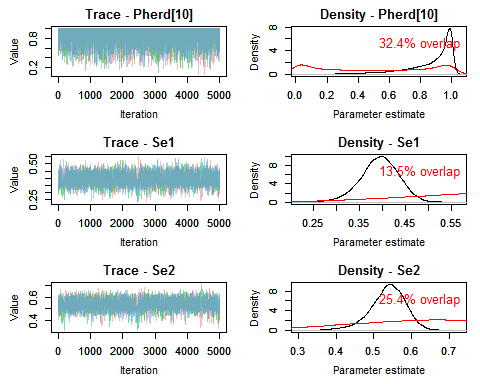

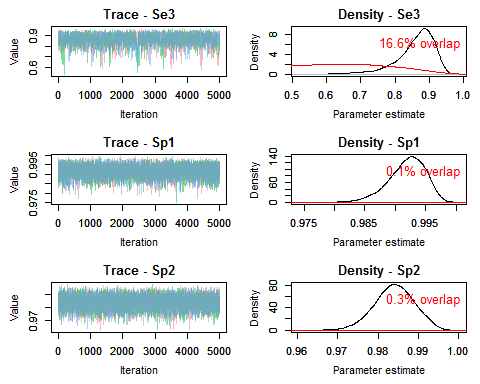

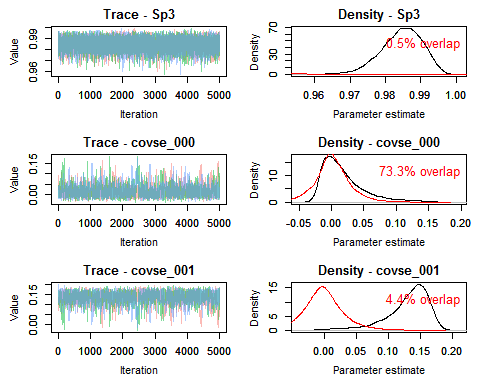

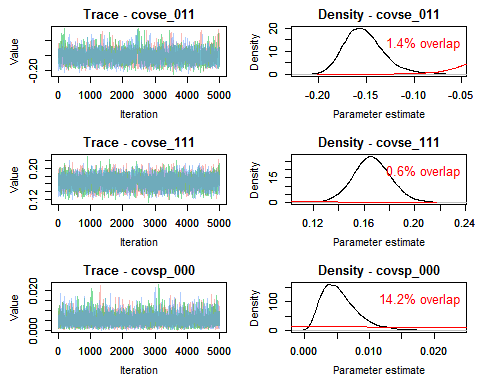

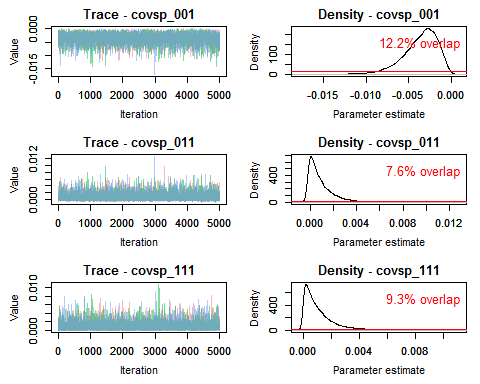

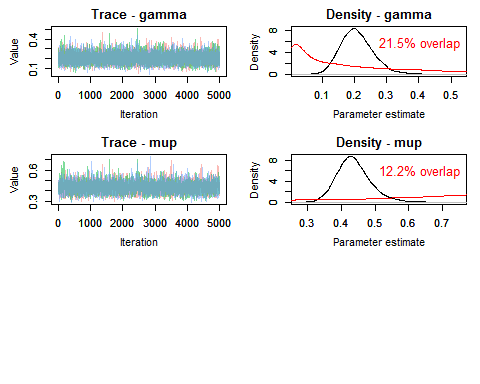


# GOATS

data_C <- subset(data, data$species == "goat")
data_C$Nherd[is.na(data_C$Nherd)] <- median(data_C$Nherd,na.rm = T)
#Creation of argument data for jags.model

N <- rowSums(data_C[,4:11])

data4jags.lclass_C <- list(Npop = nrow(data_C),
 N = N,
 Nherd = data_C$Nherd,
 n = as.matrix(data_C[,4:11]),
 Ndpt=10,
 numdpt = data_C$dpt,
 yse_000 = 1,
 yse_001 = 1,
 yse_010 = 1,
 yse_011 = 1,
 yse_100 = 1,
 yse_101 = 1,
 yse_110 = 1,
 yse_111 = 1,
 ysp_000 = 1,
 ysp_001 = 1,
 ysp_010 = 1,
 ysp_011 = 1,
 ysp_100 = 1,
 ysp_101 = 1,
 ysp_110 = 1,
 ysp_111 = 1,
 yse1 = 1,
 yse2 = 1,
 yse3 = 1,
 y_P = rep(1,nrow(data_C)))

##################
# Initialisation #
##################

# initialization ensures that the parameters respect the constraints of the model


inits <- list(list(),list(),list())

for (k in 1:3) {

 Sp1 <- rbeta(1,5,1)
 Sp2 <- rbeta(1,5,1)
 Sp3 <- rbeta(1,5,1)

 Se1 <- runif(1,1-Sp1,1)
 Se2 <- runif(1,1-Sp2,1)
 Se3 <- runif(1,1-Sp3,1)


 covse_111 <- 0
 covse_011 <- 0
 covse_001 <- 0
 covse_000 <- 0

 covsp_111 <- 0
 covsp_011 <- 0
 covsp_001 <- 0
 covsp_000 <- 0

 P <- runif(length(data_C$Nherd),1/data_C$Nherd,1)

 inits[[k]] <- list(Se1 = Se1, Se2 = Se2, Se3 = Se3,Sp1 = Sp1,
 Sp2 = Sp2, Sp3 = Sp3, covse_111 = covse_111,
 covse_011 = covse_011, covse_001 = covse_001,
 covse_000 = covse_000, covsp_111 = covsp_111,
 covsp_011 = covsp_011, covsp_001 = covsp_001,
 covsp_000 = covsp_000, P = P)
}

##################################
# Inference using MCMC algorithm #
# This step can take a few hours #
##################################
m.lclass_C<- jags.model(file = textConnection(model.lclass),
 data = data4jags.lclass_C, n.chains = 3, inits = inits)

## Compiling model graph
## Resolving undeclared variables
## Allocating nodes
## Graph information:
## Observed stochastic nodes: 225
## Unobserved stochastic nodes: 232
## Total graph size: 3750
##
## Initializing model

update(m.lclass_C, n.iter = 10000) # burnin

mcmc.lclass_C<- coda.samples(m.lclass_C, c("mup","gamma","Pherd","Se1","Se2","Se3",
 "Sp1","Sp2","Sp3","covse_000","covse_001",
 "covse_011", "covse_111","covsp_000","covsp_001",
 "covsp_011", "covsp_111"), n.iter = 100000, thin = 20)


##############################
# Estimation and diagnostics #
##############################

# Check of the convergence
gelman.diag(mcmc.lclass_C)

## Potential scale reduction factors:
##
## Point est. Upper C.I.
## Pherd[1] 1.00 1.00
## Pherd[2] 1.00 1.00
## Pherd[3] 1.00 1.00
## Pherd[4] 1.00 1.00
## Pherd[5] 1.00 1.00
## Pherd[6] 1.00 1.00
## Pherd[7] 1.00 1.00
## Pherd[8] 1.00 1.00
## Pherd[9] 1.00 1.00
## Pherd[10] 1.00 1.00
## Se1 1.00 1.01
## Se2 1.00 1.01
## Se3 1.01 1.02
## Sp1 1.00 1.01
## Sp2 1.00 1.00
## Sp3 1.00 1.00
## covse_000 1.01 1.02
## covse_001 1.01 1.02
## covse_011 1.00 1.00
## covse_111 1.01 1.02
## covsp_000 1.00 1.01
## covsp_001 1.00 1.01
## covsp_011 1.00 1.01
## covsp_111 1.00 1.00
## gamma 1.00 1.01
## mup 1.00 1.00
##
## Multivariate psrf
##
## 1.01

# Parameter estimations
summary( mcmc.lclass_C)

##
## Iterations = 11020:111000
## Thinning interval = 20
## Number of chains = 3
## Sample size per chain = 5000
##
## 1. Empirical mean and standard deviation for each variable,
## plus standard error of the mean:
##
## Mean SD Naive SE Time-series SE
## Pherd[1] 0.888806 0.090804 7.414e-04 7.322e-04
## Pherd[2] 0.804187 0.114735 9.368e-04 9.290e-04
## Pherd[3] 0.195943 0.113963 9.305e-04 9.472e-04
## Pherd[4] 0.961318 0.051788 4.228e-04 4.288e-04
## Pherd[5] 0.287104 0.129745 1.059e-03 1.059e-03
## Pherd[6] 0.188336 0.128913 1.053e-03 1.105e-03
## Pherd[7] 0.720327 0.127741 1.043e-03 1.043e-03
## Pherd[8] 0.293876 0.286831 2.342e-03 2.313e-03
## Pherd[9] 0.246840 0.125866 1.028e-03 1.019e-03
## Pherd[10] 0.907031 0.086410 7.055e-04 7.305e-04
## Se1 0.590717 0.027068 2.210e-04 5.337e-04
## Se2 0.748974 0.028908 2.360e-04 6.956e-04
## Se3 0.900110 0.026775 2.186e-04 7.758e-04
## Sp1 0.990971 0.003908 3.191e-05 8.784e-05
## Sp2 0.990216 0.004214 3.440e-05 8.549e-05
## Sp3 0.959113 0.010094 8.241e-05 1.555e-04
## covse_000 0.018199 0.021614 1.765e-04 6.501e-04
## covse_001 0.096327 0.015968 1.304e-04 4.198e-04
## covse_011 -0.106313 0.010141 8.280e-05 1.226e-04
## covse_111 0.126186 0.019012 1.552e-04 5.207e-04
## covsp_000 0.011260 0.005276 4.308e-05 1.410e-04
## covsp_001 -0.007403 0.003467 2.831e-05 8.505e-05
## covsp_011 0.001668 0.001783 1.456e-05 2.577e-05
## covsp_111 0.002545 0.002234 1.824e-05 5.399e-05
## gamma 0.270639 0.059640 4.870e-04 1.051e-03
## mup 0.675582 0.042630 3.481e-04 7.166e-04
##
## 2. Quantiles for each variable:
##
## 2.5% 25% 50% 75% 97.5%
## Pherd[1] 0.6655986 0.8386681 0.909467 0.960027 0.998465
## Pherd[2] 0.5425877 0.7334650 0.820386 0.890662 0.977677
## Pherd[3] 0.0242617 0.1091721 0.179612 0.266018 0.455039
## Pherd[4] 0.8138866 0.9480135 0.981225 0.995769 0.999955
## Pherd[5] 0.0736238 0.1895055 0.275370 0.371598 0.567667
## Pherd[6] 0.0173166 0.0873980 0.161800 0.265336 0.493971
## Pherd[7] 0.4441621 0.6368604 0.733455 0.815971 0.932466
## Pherd[8] 0.0004120 0.0467495 0.196307 0.479967 0.957271
## Pherd[9] 0.0510780 0.1503736 0.231291 0.326740 0.528823
## Pherd[10] 0.6864344 0.8631625 0.930064 0.975300 0.999645
## Se1 0.5328577 0.5734273 0.592158 0.609671 0.640077
## Se2 0.6835003 0.7321088 0.751569 0.769080 0.798664
## Se3 0.8344235 0.8866909 0.905472 0.918949 0.938623
## Sp1 0.9818498 0.9886625 0.991499 0.993788 0.997025
## Sp2 0.9804498 0.9877234 0.990792 0.993284 0.996734
## Sp3 0.9368533 0.9529616 0.959903 0.966223 0.976513
## covse_000 -0.0059702 0.0018326 0.012358 0.028407 0.074091
## covse_001 0.0588668 0.0874377 0.098231 0.107238 0.122542
## covse_011 -0.1258974 -0.1131463 -0.106474 -0.099684 -0.085996
## covse_111 0.0960997 0.1125348 0.123248 0.137151 0.170467
## covsp_000 0.0034322 0.0074462 0.010382 0.014128 0.023774
## covsp_001 -0.0155655 -0.0094156 -0.006954 -0.004857 -0.002067
## covsp_011 -0.0002810 0.0003636 0.001168 0.002481 0.006268
## covsp_111 0.0001023 0.0009076 0.001939 0.003521 0.008361
## gamma 0.1687860 0.2290963 0.265642 0.306264 0.402985
## mup 0.5935644 0.6467968 0.675373 0.703048 0.761554

######################
# Prior vs posterior #
######################


data4jags.lclass_prior_C <- list(Npop = nrow(data_C),
 N = rep(0,nrow(data_C)),
 Nherd = data_C$Nherd,
 n = matrix(data=0, nrow= nrow(data_C), ncol=8),
 Ndpt=10,
 numdpt = data_C$dpt,
 yse_000 = 1,
 yse_001 = 1,
 yse_010 = 1,
 yse_011 = 1,
 yse_100 = 1,
 yse_101 = 1,
 yse_110 = 1,
 yse_111 = 1,
 ysp_000 = 1,
 ysp_001 = 1,
 ysp_010 = 1,
 ysp_011 = 1,
 ysp_100 = 1,
 ysp_101 = 1,
 ysp_110 = 1,
 ysp_111 = 1,
 yse1 = 1,
 yse2 = 1,
 yse3 = 1,
 y_P = rep(1,nrow(data_C)))

 m.lclass_prior_C<- jags.model(file = textConnection(model.lclass), data = data4jags.lclass_prior_C, n.chains = 3, inits = inits)

## Compiling model graph
## Resolving undeclared variables
## Allocating nodes
## Graph information:
## Observed stochastic nodes: 225
## Unobserved stochastic nodes: 232
## Total graph size: 3750
##
## Initializing model

update(m.lclass_prior_C, n.iter = 10000) # burnin


 mcmc.lclass_prior_C<- coda.samples(m.lclass_prior_C, c("mup","gamma","Pherd","Se1","Se2","Se3","Sp1","Sp2","Sp3","covse_000","covse_001", "covse_011", "covse_111","covsp_000","covsp_001", "covsp_011", "covsp_111"), n.iter = 100000, thin = 20)


MCMCtrace( mcmc.lclass_C,
 ISB = FALSE,
 priors = as.matrix( mcmc.lclass_prior_C),
 pdf = FALSE,
 Rhat = FALSE,
 n.eff = FALSE)


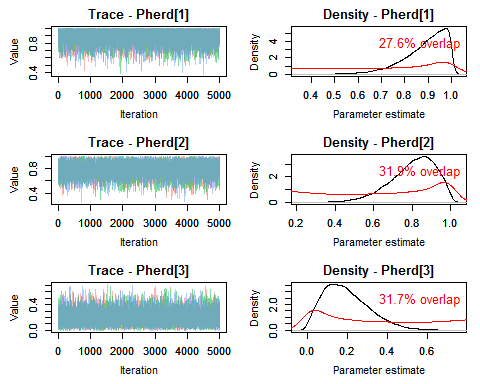

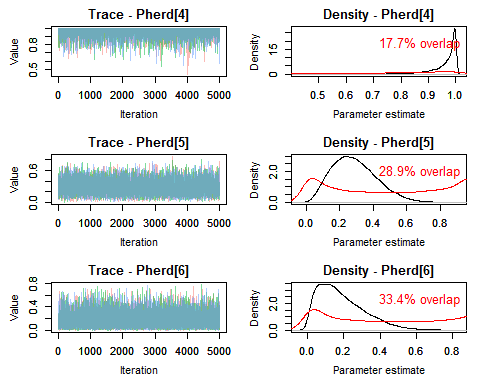

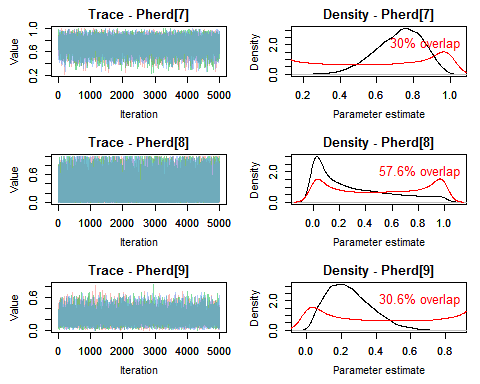

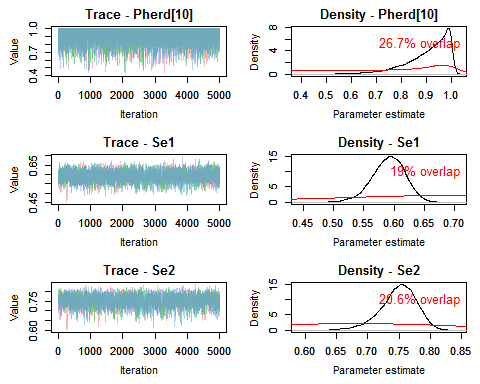

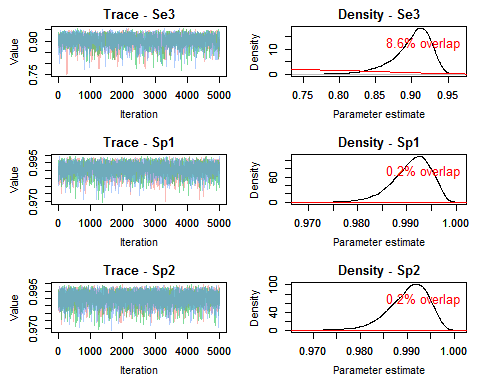

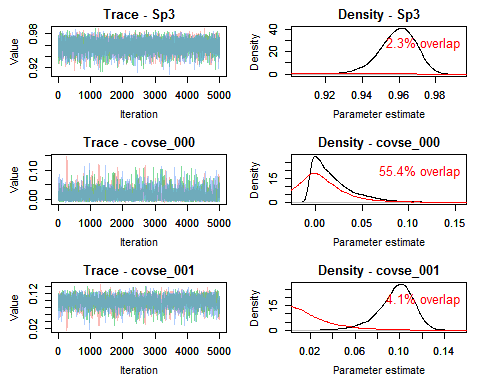

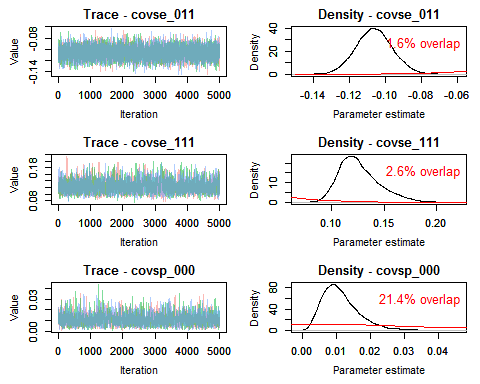

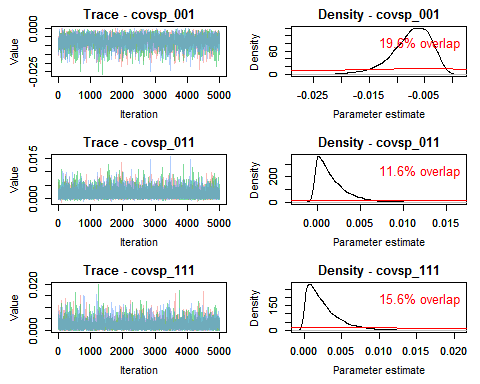

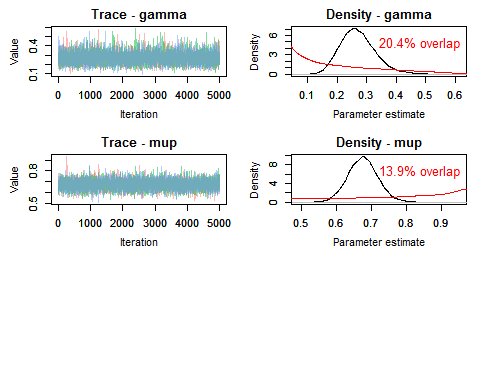


# CATTLE without 7th Department (Department G)

data_B_s7 <- subset(data, data$species == "cattle" & data$dpt!=7)
data_B_s7$Nherd <- as.integer(data_B_s7$Nherd)
data_B_s7$Nherd[is.na(data_B_s7$Nherd)] <- median(data_B_s7$Nherd,na.rm = T)
#Creation of argument data for jags.model

N <- rowSums(data_B_s7[,4:11])

data4jags.lclass_B_s7 <- list(Npop = nrow(data_B_s7),
 N = N,
 Nherd = data_B_s7$Nherd ,
 n = as.matrix(data_B_s7[,4:11]),
 Ndpt=10,
 numdpt = data_B_s7$dpt,
 yse_000 = 1,
 yse_001 = 1,
 yse_010 = 1,
 yse_011 = 1,
 yse_100 = 1,
 yse_101 = 1,
 yse_110 = 1,
 yse_111 = 1,
 ysp_000 = 1,
 ysp_001 = 1,
 ysp_010 = 1,
 ysp_011 = 1,
 ysp_100 = 1,
 ysp_101 = 1,
 ysp_110 = 1,
 ysp_111 = 1,
 yse1 = 1,
 yse2 = 1,
 yse3 = 1,
 y_P = rep(1,nrow(data_B_s7)))

##################
# Initialisation #
##################

# initialization ensures that the parameters respect the constraints of the model

inits <- list(list(),list(),list())

for (k in 1:3) {

 Sp1 <- rbeta(1,5,1)
 Sp2 <- rbeta(1,5,1)
 Sp3 <- rbeta(1,5,1)

 Se1 <- runif(1,1-Sp1,1)
 Se2 <- runif(1,1-Sp2,1)
 Se3 <- runif(1,1-Sp3,1)


 covse_111 <- 0
 covse_011 <- 0
 covse_001 <- 0
 covse_000 <- 0

 covsp_111 <- 0
 covsp_011 <- 0
 covsp_001 <- 0
 covsp_000 <- 0

 P <- runif(length(data_B_s7$Nherd),1/data_B_s7$Nherd,1)

 inits[[k]] <- list(Se1 = Se1, Se2 = Se2, Se3 = Se3,Sp1 = Sp1,
 Sp2 = Sp2, Sp3 = Sp3, covse_111 = covse_111,
 covse_011 = covse_011, covse_001 = covse_001,
 covse_000 = covse_000, covsp_111 = covsp_111,
 covsp_011 = covsp_011, covsp_001 = covsp_001,
 covsp_000 = covsp_000, P = P)
}


##################################
# Inference using MCMC algorithm #
# This step can take a few hours #
##################################
m.lclass_B_s7<- jags.model(file = textConnection(model.lclass),
 data = data4jags.lclass_B_s7, n.chains = 3, inits = inits)

## Compiling model graph
## Resolving undeclared variables
## Allocating nodes
## Graph information:
## Observed stochastic nodes: 211
## Unobserved stochastic nodes: 218
## Total graph size: 3525
##
## Initializing model

update(m.lclass_B_s7, n.iter = 10000) # burnin

mcmc.lclass_B_s7<- coda.samples(m.lclass_B_s7, c("mup","gamma","Pherd","Se1","Se2","Se3",
 "Sp1","Sp2","Sp3","covse_000","covse_001",
 "covse_011", "covse_111","covsp_000","covsp_001",
 "covsp_011", "covsp_111"), n.iter = 100000, thin = 20)


##############################
# Estimation and diagnostics #
##############################

# Check of the convergence
gelman.diag(mcmc.lclass_B_s7)

## Potential scale reduction factors:
##
## Point est. Upper C.I.
## Pherd[1] 1.00 1.00
## Pherd[2] 1.00 1.00
## Pherd[3] 1.00 1.00
## Pherd[4] 1.00 1.00
## Pherd[5] 1.00 1.00
## Pherd[6] 1.00 1.00
## Pherd[7] 1.00 1.00
## Pherd[8] 1.00 1.00
## Pherd[9] 1.00 1.00
## Pherd[10] 1.00 1.00
## Se1 1.01 1.02
## Se2 1.00 1.01
## Se3 1.03 1.07
## Sp1 1.01 1.01
## Sp2 1.01 1.01
## Sp3 1.01 1.01
## covse_000 1.04 1.10
## covse_001 1.01 1.04
## covse_011 1.00 1.00
## covse_111 1.02 1.06
## covsp_000 1.01 1.02
## covsp_001 1.01 1.02
## covsp_011 1.00 1.00
## covsp_111 1.01 1.01
## gamma 1.00 1.01
## mup 1.00 1.01
##
## Multivariate psrf
##
## 1.02

# Parameter estimations
summary( mcmc.lclass_B_s7)

##
## Iterations = 11020:111000
## Thinning interval = 20
## Number of chains = 3
## Sample size per chain = 5000
##
## 1. Empirical mean and standard deviation for each variable,
## plus standard error of the mean:
##
## Mean SD Naive SE Time-series SE
## Pherd[1] 0.105520 0.104219 8.509e-04 1.047e-03
## Pherd[2] 0.244371 0.161869 1.322e-03 2.531e-03
## Pherd[3] 0.933546 0.084951 6.936e-04 7.894e-04
## Pherd[4] 0.637507 0.178381 1.456e-03 3.304e-03
## Pherd[5] 0.673504 0.154602 1.262e-03 1.717e-03
## Pherd[6] 0.491087 0.152652 1.246e-03 1.376e-03
## Pherd[7] 0.500006 0.353125 2.883e-03 2.869e-03
## Pherd[8] 0.049260 0.065844 5.376e-04 5.868e-04
## Pherd[9] 0.107063 0.121832 9.948e-04 2.306e-03
## Pherd[10] 0.401504 0.340108 2.777e-03 2.872e-03
## Se1 0.725735 0.049883 4.073e-04 1.911e-03
## Se2 0.624974 0.053123 4.337e-04 1.789e-03
## Se3 0.884081 0.040273 3.288e-04 2.425e-03
## Sp1 0.959846 0.008368 6.832e-05 3.939e-04
## Sp2 0.975410 0.005693 4.648e-05 2.817e-04
## Sp3 0.948348 0.014280 1.166e-04 5.741e-04
## covse_000 0.022942 0.029442 2.404e-04 1.883e-03
## covse_001 0.132512 0.025582 2.089e-04 1.120e-03
## covse_011 -0.139659 0.015623 1.276e-04 3.641e-04
## covse_111 0.161578 0.025306 2.066e-04 9.802e-04
## covsp_000 0.046884 0.010309 8.417e-05 5.849e-04
## covsp_001 -0.026884 0.006128 5.004e-05 3.266e-04
## covsp_011 0.001353 0.001634 1.334e-05 1.701e-05
## covsp_111 0.018810 0.005058 4.130e-05 2.563e-04
## gamma 0.224459 0.053401 4.360e-04 1.143e-03
## mup 0.482686 0.060561 4.945e-04 2.104e-03
##
## 2. Quantiles for each variable:
##
## 2.5% 25% 50% 75% 97.5%
## Pherd[1] 2.889e-04 0.0227425 0.075065 0.157939 0.375063
## Pherd[2] 9.850e-03 0.1137744 0.221339 0.350965 0.604400
## Pherd[3] 6.894e-01 0.9061425 0.967276 0.992517 0.999918
## Pherd[4] 2.843e-01 0.5116050 0.642186 0.770698 0.960977
## Pherd[5] 3.599e-01 0.5687668 0.679238 0.786463 0.955049
## Pherd[6] 2.083e-01 0.3819809 0.486205 0.597533 0.792252
## Pherd[7] 1.769e-03 0.1459879 0.499969 0.853074 0.998149
## Pherd[8] 3.974e-05 0.0051048 0.023211 0.068053 0.238538
## Pherd[9] 1.337e-04 0.0138627 0.060616 0.160107 0.431614
## Pherd[10] 8.447e-04 0.0800114 0.315006 0.709107 0.995779
## Se1 6.196e-01 0.6955118 0.727656 0.759822 0.814729
## Se2 5.185e-01 0.5899118 0.625673 0.661647 0.722572
## Se3 7.895e-01 0.8662840 0.890593 0.910364 0.939997
## Sp1 9.429e-01 0.9543498 0.960022 0.965500 0.976009
## Sp2 9.631e-01 0.9718399 0.975731 0.979387 0.985792
## Sp3 9.219e-01 0.9379384 0.947759 0.958934 0.975885
## covse_000 -7.166e-03 0.0031441 0.015024 0.033251 0.099650
## covse_001 7.553e-02 0.1187673 0.135225 0.149726 0.173110
## covse_011 -1.682e-01 -0.1503775 -0.140579 -0.129744 -0.106713
## covse_111 1.172e-01 0.1450845 0.159244 0.175537 0.219645
## covsp_000 2.694e-02 0.0399569 0.046518 0.053550 0.068047
## covsp_001 -3.920e-02 -0.0308746 -0.026865 -0.022782 -0.014737
## covsp_011 -1.005e-03 0.0001897 0.001069 0.002218 0.005316
## covsp_111 9.776e-03 0.0152736 0.018491 0.021992 0.029685
## gamma 1.266e-01 0.1879650 0.221775 0.258388 0.337213
## mup 3.750e-01 0.4403959 0.479369 0.520476 0.610574

######################
# Prior vs posterior #
######################


data4jags.lclass_prior_B_s7 <- list(Npop = nrow(data_B_s7),
 N = rep(0,nrow(data_B_s7)),
 Nherd = data_B_s7$Nherd,
 n = matrix(data=0, nrow= nrow(data_B_s7), ncol=8),
 Ndpt=10,
 numdpt = data_B_s7$dpt,
 yse_000 = 1,
 yse_001 = 1,
 yse_010 = 1,
 yse_011 = 1,
 yse_100 = 1,
 yse_101 = 1,
 yse_110 = 1,
 yse_111 = 1,
 ysp_000 = 1,
 ysp_001 = 1,
 ysp_010 = 1,
 ysp_011 = 1,
 ysp_100 = 1,
 ysp_101 = 1,
 ysp_110 = 1,
 ysp_111 = 1,
 yse1 = 1,
 yse2 = 1,
 yse3 = 1,
 y_P = rep(1,nrow(data_B_s7)))

 m.lclass_prior_B_s7<- jags.model(file = textConnection(model.lclass), data = data4jags.lclass_prior_B_s7, n.chains = 3, inits = inits)

## Compiling model graph
## Resolving undeclared variables
## Allocating nodes
## Graph information:
## Observed stochastic nodes: 211
## Unobserved stochastic nodes: 218
## Total graph size: 3525
##
## Initializing model

update(m.lclass_prior_B_s7, n.iter = 10000) # burnin


 mcmc.lclass_prior_B_s7<- coda.samples(m.lclass_prior_B_s7, c("mup","gamma","Pherd","Se1","Se2","Se3","Sp1","Sp2","Sp3","covse_000","covse_001", "covse_011", "covse_111","covsp_000","covsp_001", "covsp_011", "covsp_111"), n.iter = 100000, thin = 20)


MCMCtrace( mcmc.lclass_B_s7,
 ISB = FALSE,
 priors = as.matrix( mcmc.lclass_prior_B_s7),
 pdf = FALSE,
 Rhat = FALSE,
 n.eff = FALSE)


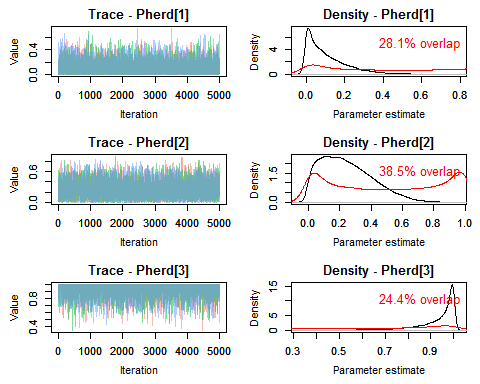

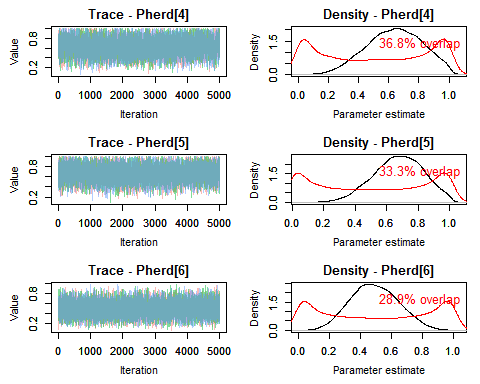

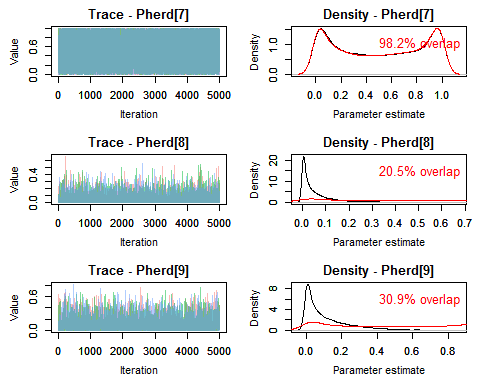

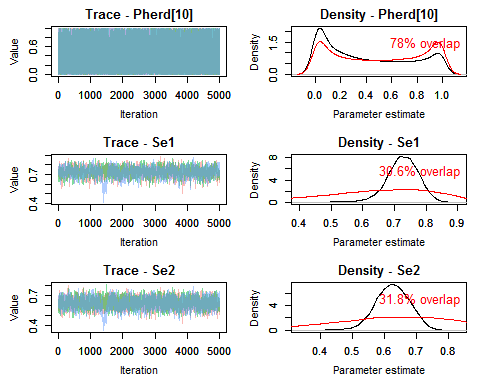

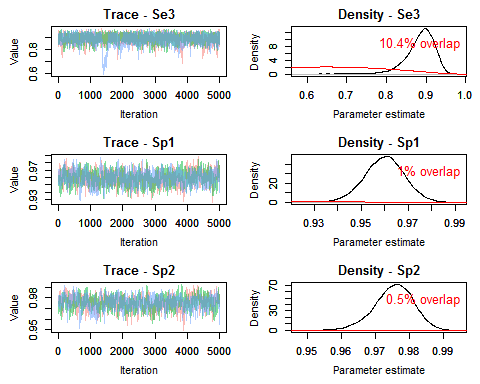

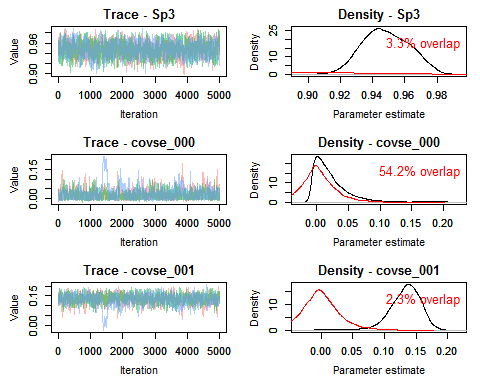

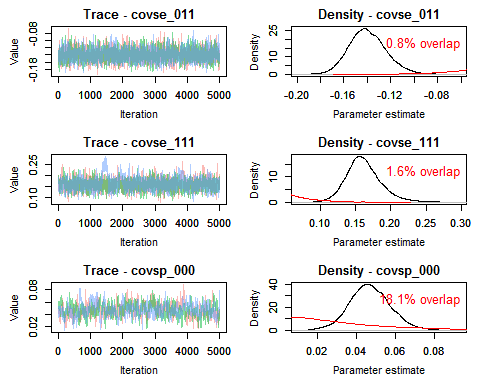

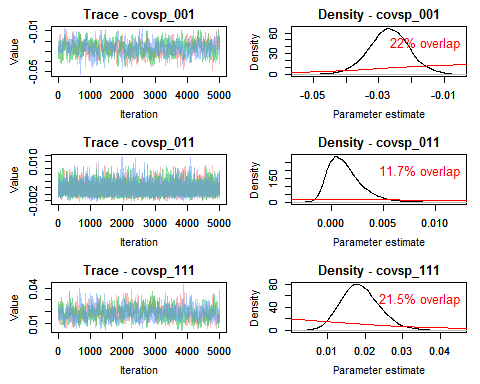

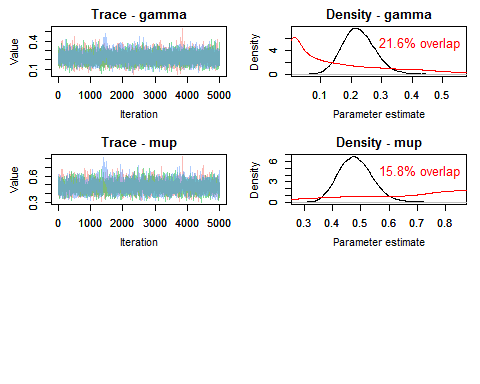


# CATTLE only in the 7th Department

data_B_7 <- subset(data, data$species == "cattle" & data$dpt==7)
data_B_7$Nherd[is.na(data_B_7$Nherd)] <- median(data_B_7$Nherd,na.rm = T)
#Creation of argument data for jags.model

N <- rowSums(data_B_7[,4:11])

data4jags.lclass_B_7 <- list(Npop = nrow(data_B_7),
 N = N,
 Nherd = data_B_7$Nherd,
 n = as.matrix(data_B_7[,4:11]),
 Ndpt=10,
 numdpt = data_B_7$dpt,
 yse_000 = 1,
 yse_001 = 1,
 yse_010 = 1,
 yse_011 = 1,
 yse_100 = 1,
 yse_101 = 1,
 yse_110 = 1,
 yse_111 = 1,
 ysp_000 = 1,
 ysp_001 = 1,
 ysp_010 = 1,
 ysp_011 = 1,
 ysp_100 = 1,
 ysp_101 = 1,
 ysp_110 = 1,
 ysp_111 = 1,
 yse1 = 1,
 yse2 = 1,
 yse3 = 1,
 y_P = rep(1,nrow(data_B_7)))

##################
# Initialisation #
##################

# initialization ensures that the parameters respect the constraints of the model

inits <- list(list(),list(),list())
for (k in 1:3) {

 Sp1 <- rbeta(1,5,1)
 Sp2 <- rbeta(1,5,1)
 Sp3 <- rbeta(1,5,1)

 Se1 <- runif(1,1-Sp1,1)
 Se2 <- runif(1,1-Sp2,1)
 Se3 <- runif(1,1-Sp3,1)


 covse_111 <- 0
 covse_011 <- 0
 covse_001 <- 0
 covse_000 <- 0

 covsp_111 <- 0
 covsp_011 <- 0
 covsp_001 <- 0
 covsp_000 <- 0

 P <- runif(length(data_B_7$Nherd),1/data_B_7$Nherd,1)

 inits[[k]] <- list(Se1 = Se1, Se2 = Se2, Se3 = Se3,Sp1 = Sp1,
 Sp2 = Sp2, Sp3 = Sp3, covse_111 = covse_111,
 covse_011 = covse_011, covse_001 = covse_001,
 covse_000 = covse_000, covsp_111 = covsp_111,
 covsp_011 = covsp_011, covsp_001 = covsp_001,
 covsp_000 = covsp_000, P = P)
}


##################################
# Inference using MCMC algorithm #
# This step can take a few hours #
##################################
m.lclass_B_7<- jags.model(file = textConnection(model.lclass),
 data = data4jags.lclass_B_7, n.chains = 3, inits = inits)

## Compiling model graph
## Resolving undeclared variables
## Allocating nodes
## Graph information:
## Observed stochastic nodes: 39
## Unobserved stochastic nodes: 46
## Total graph size: 545
##
## Initializing model

update(m.lclass_B_7, n.iter = 10000) # burnin

mcmc.lclass_B_7<- coda.samples(m.lclass_B_7, c("mup","gamma","Pherd","Se1","Se2","Se3",
 "Sp1","Sp2","Sp3","covse_000","covse_001",
 "covse_011", "covse_111","covsp_000","covsp_001",
 "covsp_011", "covsp_111"), n.iter = 100000, thin = 20)


##############################
# Estimation and diagnostics #
##############################

# Check of the convergence
gelman.diag(mcmc.lclass_B_7)

## Potential scale reduction factors:
##
## Point est. Upper C.I.
## Pherd[1] 1.00 1.00
## Pherd[2] 1.00 1.00
## Pherd[3] 1.00 1.00
## Pherd[4] 1.00 1.00
## Pherd[5] 1.00 1.00
## Pherd[6] 1.00 1.00
## Pherd[7] 1.01 1.02
## Pherd[8] 1.00 1.00
## Pherd[9] 1.00 1.00
## Pherd[10] 1.00 1.00
## Se1 1.00 1.00
## Se2 1.00 1.01
## Se3 1.00 1.00
## Sp1 1.01 1.01
## Sp2 1.00 1.00
## Sp3 1.00 1.01
## covse_000 1.00 1.00
## covse_001 1.00 1.00
## covse_011 1.00 1.00
## covse_111 1.00 1.00
## covsp_000 1.00 1.00
## covsp_001 1.00 1.00
## covsp_011 1.00 1.00
## covsp_111 1.00 1.00
## gamma 1.00 1.01
## mup 1.04 1.12
##
## Multivariate psrf
##
## 1.03

# Parameter estimations
summary( mcmc.lclass_B_7)

##
## Iterations = 11020:111000
## Thinning interval = 20
## Number of chains = 3
## Sample size per chain = 5000
##
## 1. Empirical mean and standard deviation for each variable,
## plus standard error of the mean:
##
## Mean SD Naive SE Time-series SE
## Pherd[1] 0.4977428 0.353409 2.886e-03 2.886e-03
## Pherd[2] 0.4948752 0.353610 2.887e-03 2.817e-03
## Pherd[3] 0.5015498 0.354332 2.893e-03 2.895e-03
## Pherd[4] 0.4968829 0.352736 2.880e-03 2.852e-03
## Pherd[5] 0.5009665 0.353481 2.886e-03 2.909e-03
## Pherd[6] 0.4991912 0.353342 2.885e-03 2.885e-03
## Pherd[7] 0.1166248 0.149595 1.221e-03 2.975e-03
## Pherd[8] 0.5010739 0.353359 2.885e-03 2.885e-03
## Pherd[9] 0.5036434 0.352017 2.874e-03 2.874e-03
## Pherd[10] 0.5041853 0.351685 2.871e-03 2.991e-03
## Se1 0.5703087 0.169615 1.385e-03 1.407e-03
## Se2 0.4048533 0.218706 1.786e-03 2.627e-03
## Se3 0.4778394 0.213099 1.740e-03 2.236e-03
## Sp1 0.7519080 0.043191 3.527e-04 6.348e-04
## Sp2 0.9779956 0.010866 8.872e-05 1.336e-04
## Sp3 0.9670599 0.016367 1.336e-04 2.409e-04
## covse_000 -0.0031626 0.035568 2.904e-04 2.904e-04
## covse_001 0.0029505 0.032665 2.667e-04 2.728e-04
## covse_011 -0.0002869 0.031344 2.559e-04 2.591e-04
## covse_111 0.0045670 0.036184 2.954e-04 3.044e-04
## covsp_000 0.0166960 0.010222 8.346e-05 1.501e-04
## covsp_001 -0.0103830 0.007098 5.795e-05 1.037e-04
## covsp_011 0.0038960 0.004340 3.544e-05 4.295e-05
## covsp_111 0.0064500 0.005376 4.389e-05 7.273e-05
## gamma 0.2262203 0.242658 1.981e-03 8.445e-03
## mup 0.5742407 0.300564 2.454e-03 1.857e-02
##
## 2. Quantiles for each variable:
##
## 2.5% 25% 50% 75% 97.5%
## Pherd[1] 0.0013722 0.1458289 0.4933997 0.850947 0.9984201
## Pherd[2] 0.0012795 0.1424275 0.4854657 0.846255 0.9984794
## Pherd[3] 0.0017747 0.1447957 0.5087593 0.855786 0.9980810
## Pherd[4] 0.0015883 0.1451736 0.4934034 0.852357 0.9983804
## Pherd[5] 0.0016569 0.1459209 0.5029634 0.854364 0.9982446
## Pherd[6] 0.0015029 0.1445215 0.5025127 0.852506 0.9983997
## Pherd[7] 0.0001656 0.0163393 0.0643985 0.158666 0.5602526
## Pherd[8] 0.0014776 0.1493027 0.4968268 0.854908 0.9985634
## Pherd[9] 0.0019334 0.1567040 0.5054887 0.853290 0.9983397
## Pherd[10] 0.0016022 0.1553774 0.5093272 0.852978 0.9983897
## Se1 0.2768264 0.4365584 0.5654800 0.701121 0.8871553
## Se2 0.0654123 0.2243359 0.3803677 0.568657 0.8451895
## Se3 0.1003891 0.3113351 0.4730701 0.644738 0.8695845
## Sp1 0.6766127 0.7255392 0.7500379 0.774017 0.8506224
## Sp2 0.9523454 0.9718410 0.9796566 0.985990 0.9941603
## Sp3 0.9306567 0.9567857 0.9685184 0.979565 0.9927753
## covse_000 -0.0764386 -0.0228412 -0.0032519 0.017171 0.0687538
## covse_001 -0.0619604 -0.0162262 0.0017735 0.021290 0.0721754
## covse_011 -0.0647417 -0.0179418 -0.0003225 0.016823 0.0660794
## covse_111 -0.0697503 -0.0161515 0.0043859 0.024395 0.0803436
## covsp_000 0.0010968 0.0092338 0.0153091 0.022720 0.0405070
## covsp_001 -0.0268401 -0.0145800 -0.0094132 -0.005282 0.0006987
## covsp_011 -0.0004371 0.0008539 0.0026006 0.005557 0.0156654
## covsp_111 0.0001977 0.0024267 0.0051345 0.009123 0.0200741
## gamma 0.0003357 0.0287121 0.1332526 0.363227 0.8326769
## mup 0.0741489 0.3047123 0.5672102 0.871727 0.9984571

######################
# Prior vs posterior #
######################


data4jags.lclass_prior_B_7 <- list(Npop = nrow(data_B_7),
 N = rep(0,nrow(data_B_7)),
 Nherd = data_B_7$Nherd,
 n = matrix(data=0, nrow= nrow(data_B_7), ncol=8),
 Ndpt=10,
 numdpt = data_B_7$dpt,
 yse_000 = 1,
 yse_001 = 1,
 yse_010 = 1,
 yse_011 = 1,
 yse_100 = 1,
 yse_101 = 1,
 yse_110 = 1,
 yse_111 = 1,
 ysp_000 = 1,
 ysp_001 = 1,
 ysp_010 = 1,
 ysp_011 = 1,
 ysp_100 = 1,
 ysp_101 = 1,
 ysp_110 = 1,
 ysp_111 = 1,
 yse1 = 1,
 yse2 = 1,
 yse3 = 1,
 y_P = rep(1,nrow(data_B_7)))

 m.lclass_prior_B_7<- jags.model(file = textConnection(model.lclass), data = data4jags.lclass_prior_B_7, n.chains = 3, inits = inits)

## Compiling model graph
## Resolving undeclared variables
## Allocating nodes
## Graph information:
## Observed stochastic nodes: 39
## Unobserved stochastic nodes: 46
## Total graph size: 545
##
## Initializing model

update(m.lclass_prior_B_7, n.iter = 10000) # burnin


 mcmc.lclass_prior_B_7<- coda.samples(m.lclass_prior_B_7, c("mup","gamma","Pherd","Se1","Se2","Se3","Sp1","Sp2","Sp3","covse_000","covse_001", "covse_011", "covse_111","covsp_000","covsp_001", "covsp_011", "covsp_111"), n.iter = 100000, thin = 20)


MCMCtrace( mcmc.lclass_B_7,
 ISB = FALSE,
 priors = as.matrix( mcmc.lclass_prior_B_7),
 pdf = FALSE,
 Rhat = FALSE,
 n.eff = FALSE)


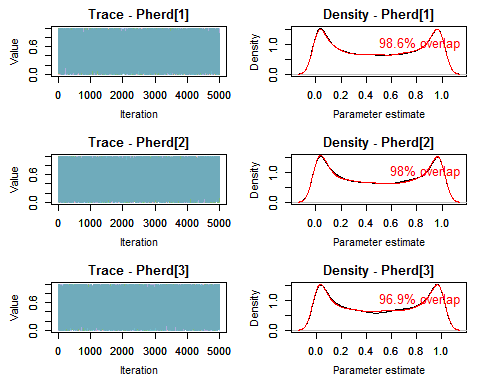

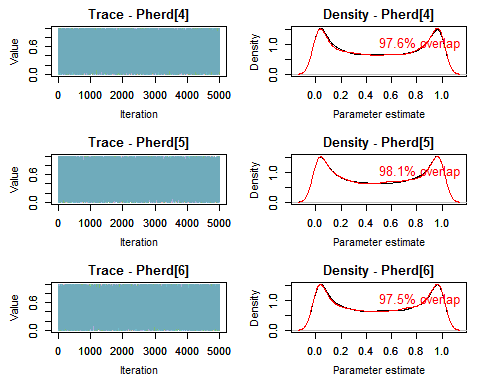

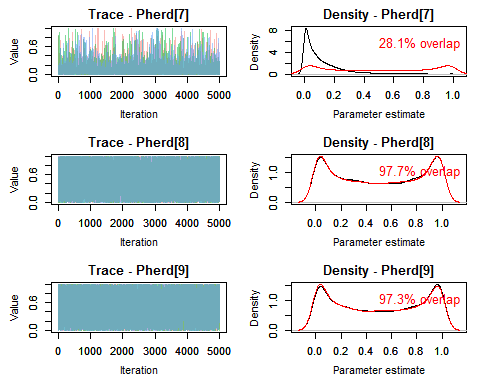

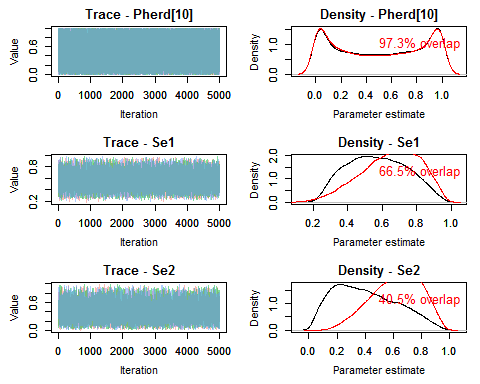

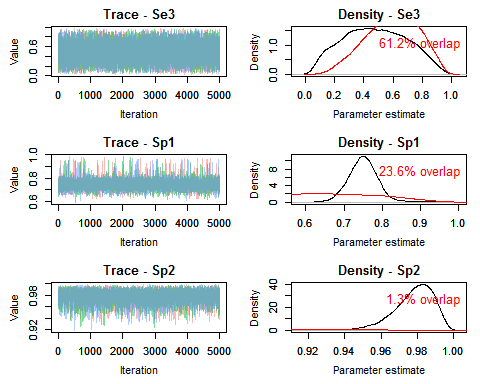

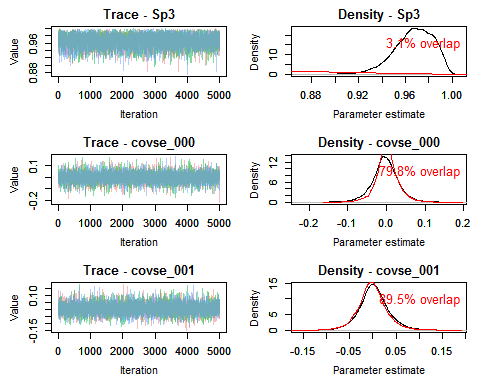

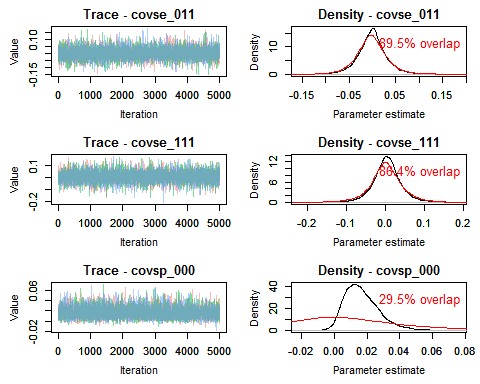

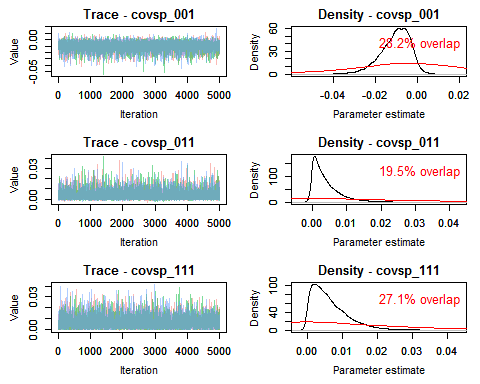

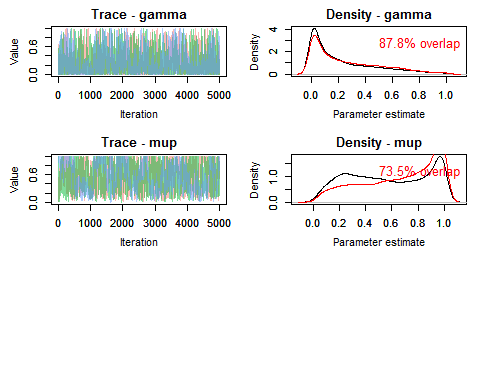

Supplement: Supplementary file 2 — Additional file 2: Output of the model. R-Markdown Word document containing R code, summary of the models, Gelman and rubin’s statistics, trace plots and prior vs posterior density plots. [file 13567_2021_926_MOESM2_ESM.docx]
